# Designing Microbe–Semiconductor Interfaces for Semibiological Photosynthesis

**DOI:** 10.1021/acs.chemrev.5c00900

**Published:** 2026-04-03

**Authors:** Wentao Song, Glenn Quek, Marion I. M. Short, Wanrong Li, Erwin Reisner, Bin Liu

**Affiliations:** † Department of Chemical and Biomolecular Engineering, 37580 National University of Singapore, 4 Engineering Drive 4, Singapore 117585, Singapore; ‡ Yusuf Hamied Department of Chemistry, 2152 University of Cambridge, Cambridge CB2 1EW, U.K.

## Abstract

Integrating semiconductors with microorganisms is attracting significant attention as a sustainable platform for solar-to-chemical conversion. This semibiological design combines the excellent light harvesting ability of semiconductor materials with intracellular biocatalytic pathways to enable efficient solar energy conversion into complex products with high selectivity. However, the effectiveness of this interdisciplinary biohybrid approach relies on a complex interfacial biotic–abiotic interaction, and it remains challenging to construct efficient and stable microbe–semiconductor systems for practical applications. In this review, we provide a systematic overview of the fundamental mechanisms behind microbe–semiconductor systems with an emphasis on interfacial electron transfer and highlight recent advancements in the assembly of biohybrids for solar-driven biosynthesis using nonphotosynthetic bacteria. First, we provide a comprehensive introduction of semibiological photosynthesis with an emphasis on extracellular electron transfer at the biotic–abiotic interfaces. Then, we discuss the engineering of biohybrid interfaces, the characterization of microbe–semiconductor interfacial electron transfer, and their deployment in solar-to-chemical conversion. We conclude by exploring the challenges in developing and optimizing biotic–abiotic interfaces as well as providing an outlook for potential future innovations. This review therefore presents the basic principles and provides guidance for the development of semibiological photosynthetic systems.

## Graphical abstract

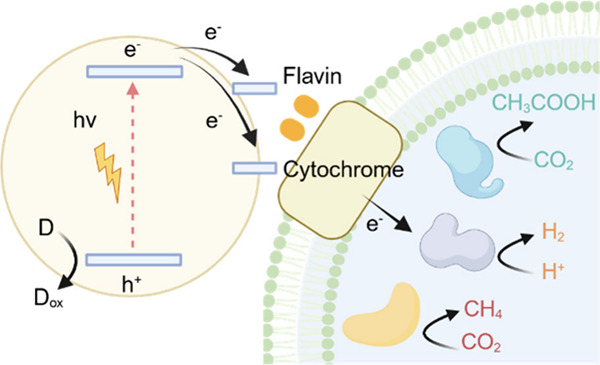

## 1 Introduction

The global transition toward sustainable energy and chemical manufacturing has created a pressing need for technologies capable of efficiently capturing solar energy and storing it in chemical bonds to produce fuels and chemicals. Natural photosynthesis has long served as a model process for this photocatalytic reaction, but its low solar-to-biomass conversion efficiency (typically 1–2%) and limited product scope in the form of sugars have motivated the development of artificial and hybrid alternatives.^1^ Artificial photosynthetic systems based on synthetic materials offer significantly higher efficiencies in light harvesting.^2–4^ For example, the highest efficiency of tandem solar cell-electrolysis systems for solar-to-hydrogen has exceeded 30%.^5^ Despite these advances, purely synthetic devices are generally poor at performing complex chemical transformations. Moreover, most semiconductor photocatalysts require the incorporation of cocatalysts to carry out controlled reactions and typically produce only simple molecules. For example, the reduction of CO_2_ commonly yields only C_1_ products such as CO and HCOOH, while achieving limited selectivity and long-term stability for reactions exceeding two-electron reductions.^6,7^

In contrast, biological organisms possess intricate metabolic processes comprised of enzymatic networks that can transform simple molecules such as CO_2_, N_2_, and H_2_O into complex chemicals with high selectivity. Their ability to self-replicate and self-repair further supports autonomous long-term operational stability and thus provides a blueprint for a circular catalytic system. The complementary strengths of synthetic light absorbers and biological networks have spurred interest in integrating efficient semiconductors with the metabolic flexibility of microbes.^8^ The resulting microbe–semiconductor biohybrid systems couple photoactive materials with living microbial systems to achieve solar-driven biosynthesis, but critical to their function and design is efficient and controlled interfacial electron transfer.^9^ The objective is not to directly mimic natural photosynthesis but to overcome its inherent limitations by synergistically integrating abiotic and biotic components that could be modularly tuned. Furthermore, their performance can be significantly enhanced through strategic material design and microbial genetic engineering, offering broad opportunities for optimization.

The concept of semibiological photosynthesis has evolved as a bridge between purely abiotic photocatalytic systems and biological metabolism. In this framework, microbe–semiconductor biohybrids stand out due to their ability to channel solar energy into biologically catalyzed redox reactions, enabling not only hydrogen evolution, carbon fixation to simple products, but also nitrogen reduction and the biosynthesis of value-added organic chemicals.^10^ Central to their function is interfacial electron transfer from the semiconductor to microbial redox pathways for biosynthesis.

Two main strategies have been developed to interface semiconductors with microbes for semibiological photosynthesis. The whole-cell photosensitization strategy involves attaching semiconductor nanoparticles to the microbial surface or incorporating them within the cell.^11–14^ Upon illumination, these particles inject electrons directly into microbial redox pathways, enabling light-driven metabolism without the need for external circuitry. In contrast, in the photoelectrochemical (PEC) approach for semibiological photosynthesis, biocatalysts are coupled to illuminated semiconductor electrodes within a bioelectrochemical system.^15,16^ Photogenerated electrons are transferred to the cells via direct or mediated electron transfer pathways, enabling redox reactions, while maintaining spatial separation between light absorption and catalysis. Notably, while electron injections into reductive pathways (e.g., CO_2_ or N_2_ fixation) is the primary topic for current studies and the key focus of this review, oxidative coupling to microbial metabolism (i.e., electron extraction) is also an attractive yet underexplored possibility.

In this review, we provide a comprehensive overview of the design of microbe–semiconductor interfaces in biohybrids for solar-driven biosynthesis (Figure 1). Distinct from existing reviews on semiartificial photosynthesis that primarily emphasize material platforms or reaction outputs,^17–19^ we center our discussion on the fundamental biotic–abiotic interactions that govern interfacial electron transfer across the microbe–semiconductor interface as well as characterization techniques. By systematically analyzing how microbial electron transfer pathways couple with semiconductor properties and interfacial architectures, we aim to understand the underlying principles that achieve efficient interfacial electron transfer and high solar-to-chemical efficiency in semibiological photosynthesis using nonphotosynthetic bacteria.

**Figure 1:**
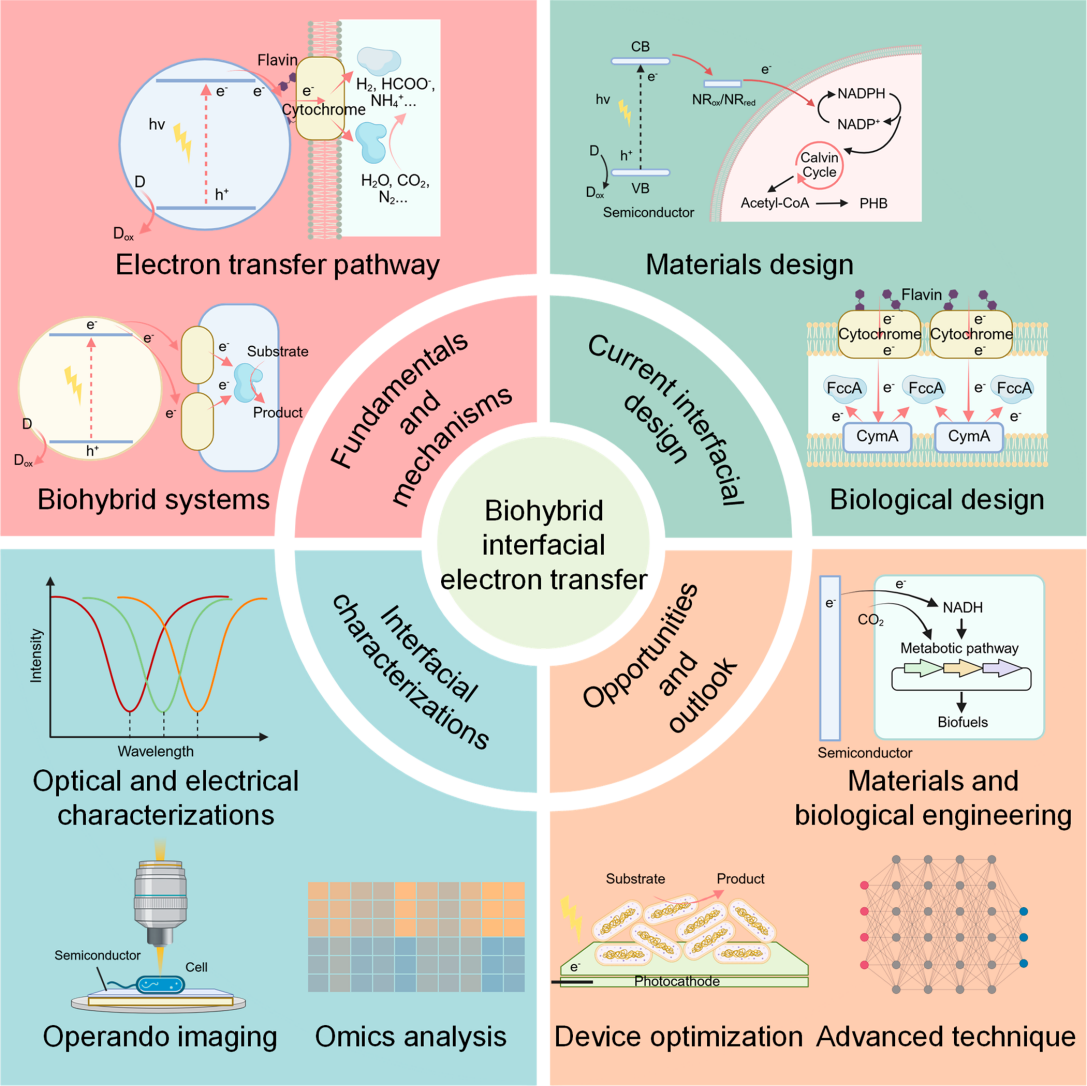
Schematic illustration of unravelling microbe–semiconductor interfacial electron transfer for efficient solar-driven biosynthesis: fundamentals and mechanisms, current design of microbe–semiconductor biohybrid interfaces, characterizations of biohybrid interfacial electron transfer, and future opportunities and outlook.

We begin by examining the fundamental concepts in this field, drawing comparisons among natural photosynthesis, artificial photocatalysis, and biohybrid approaches (section 2). We then delve into the core principles of interfacial electron transfer (section 3), covering extracellular electron transfer (EET) mechanisms, semiconductor material selection, and bidirectional electron transfer pathways across biohybrid interfaces. We explore state-of-the-art interfacial design strategies for assembling microbe–semiconductor hybrids (section 4), including both material design (e.g., engineering conductive layers and surface modification) and biological engineering (e.g., electron transfer pathway engineering and metabolic rewiring). We then highlight emerging characterization techniques such as optical characterizations, photoelectrochemical characterizations, operando imaging, and omics analyses (section 5). We review practical applications of these systems (section 6), ranging from fundamental mechanistic studies to the solar-driven production of hydrogen-, ammonia-, and carbon-based fuels. Finally, we outline current challenges and future directions (section 7), including opportunities in materials optimization, microbial engineering, advanced techniques, and device integration. This scope aims to provide a unified framework for understanding and designing microbe–semiconductor interfaces with the goal to provide a path to the realization of efficient, scalable, and versatile platforms for solar-to-chemical (STC) conversion.

## 2 Fundamentals of Semibiological Photosynthesis

### 2.1 Natural Photosynthesis

Natural photosynthesis is the process in which photoautotrophs, such as cyanobacteria, algae, and higher plants, store solar energy in chemical bonds. During this process, CO_2_ and water are converted into O_2_ and carbohydrates, such as glucose. Photosynthesis is generally comprised of light and dark reactions using the photosynthetic proteins and Calvin cycle, respectively.

The light reactions harvest photons using chlorophyll and carotenoid pigments present in the biological light absorbers photosystem II (PSII) and photosystem I (PSI) (Figure 2a).^17,20^ PSII generates electron–hole pairs upon light irradiation, using the holes to oxidize water to O_2_ and the excited electrons to reduce the terminal electron acceptor plastoquinone B to plastohydroquinone B. The electrons in the reduced plastohydroquinone are transferred via cytochrome *b*_6_ *f* and plastocyanin (PC) to the holes generated by light irradiation of PSI. The excited electrons in PSI reduce nicotinamide adenine dinucleotide phosphate (NADP^+^) to reduced nicotinamide adenine dinucleotide phosphate (NADPH) via the electron transport chain, completing the Z-scheme electron transfer chain.^21,22^ The overall process of charge dislocation across PSII and PSI generates a proton gradient, which drives the synthesis of adenosine triphosphate (ATP), a biological energy carrier, by ATP synthase using adenosine diphosphate (ADP). Hence, the light reactions catalyze the transfer of electrons from water, NADP^+^ and ADP to produce NADPH and generate ATP.^21^

**Figure 2:**
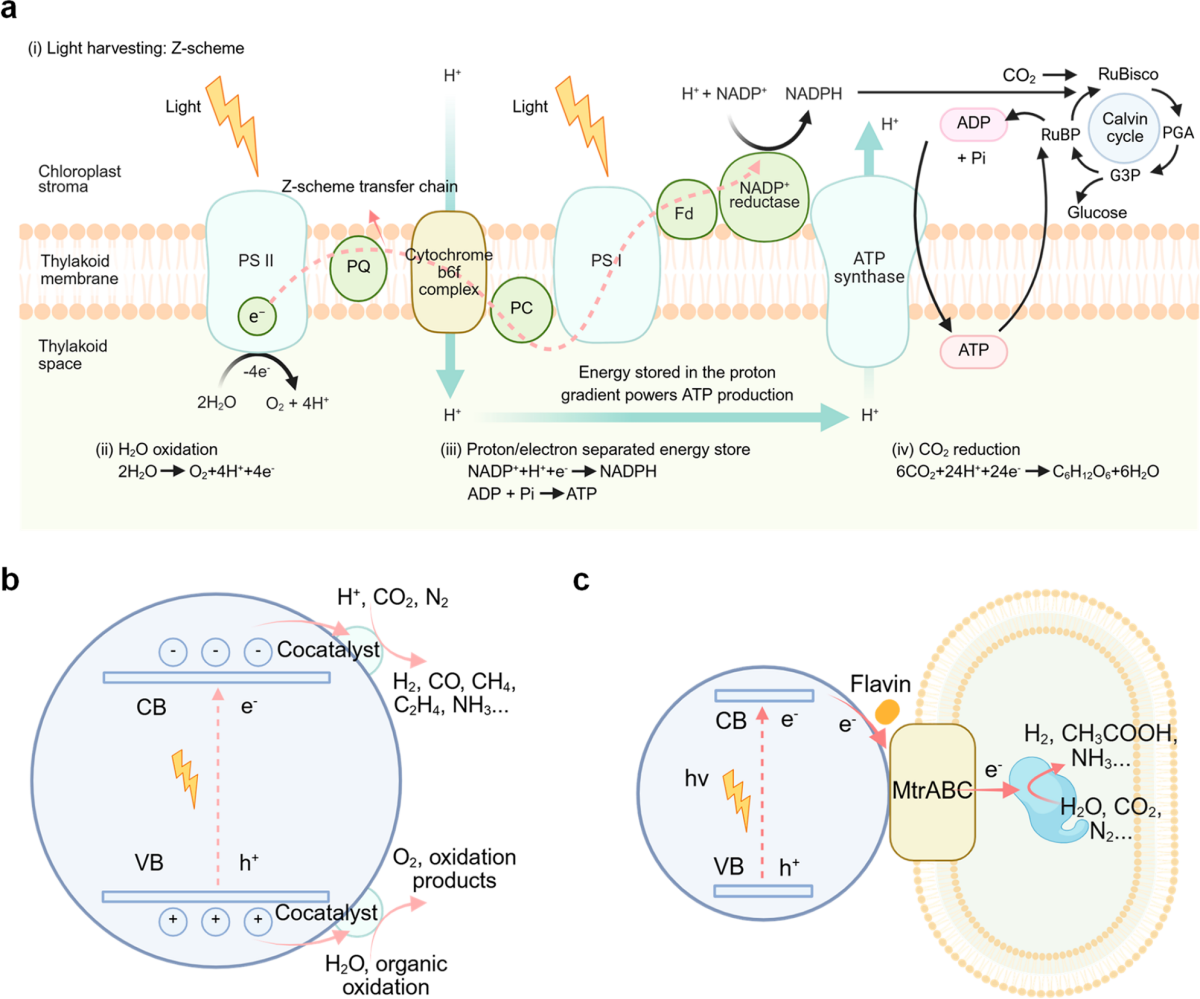
Scheme of natural photosynthesis, artificial photosynthesis, and semibiological photosynthesis. (a) Schematic representation of natural photosynthesis processes in thylakoid membranes. PS, photosystem; Fd, ferredoxin; PQ, plastoquinone; PC, plastocyanin; Pi, inorganic phosphate; RuBisco, ribulose-1,5-bisphosphate carboxylase/oxygenase; PGA, 3-phosphoglycerate; G3P, glyceraldehyde 3-phosphate; RuBP, ribulose 1,5-bisphosphate. (b) Scheme of artificial photosynthesis. (c) Schematic illustration of semibiological photosynthesis. Reproduced with permission from ref 21. Copyright 2023 Springer Nature.

The dark reactions then use the NADPH and ATP generated in the light reactions to reduce atmospheric CO_2_ to carbohydrates via the Calvin cycle.^22^ During the Calvin cycle, CO_2_ and water react with the carbon acceptor ribulose-1,5-bisphosate to produce two molecules of 3-phosphoglycerate, which can subsequently be reduced by using NADPH and ATP to produce glyceraldehyde-3-phosphate. Glyceraldehyde-3-phosphate is a key metabolite which can be used for the synthesis of carbohydrates such as glucose. The ribulose-1,5-bisphosphate is then regenerated to close the Calvin cycle. The reaction of CO_2_ with ribulose-1,5-bisphosphate is catalyzed by the ribulose-1,5-bisphophate carboxylase/oxygenase (Rubisco) enzyme. Rubisco is a powerful enzyme that can operate on atmospheric CO_2_, but it displays slow kinetics and low selectivity,^23^ often reacting with O_2_ rather than CO_2_.^24^

Natural photosynthesis is thus a powerful process for converting abundant and renewable feedstocks (air and water) into multicarbon products like glucose powered by solar energy.^17^ Photosynthetic microorganisms such as cyanobacteria and algae have been engineered to enable a wide range of solar-to-fuel conversions, including the production of hydrogen gas, ethanol, ethylene, and butanol.^25–28^ However, the overall solar-to-biomass efficiency of natural photosynthesis is only between 0.2 to 4.2%.^1,4^ One reason for this low efficiency is that biological light absorbers can utilize only about 47% of sunlight, as they are unable to absorb green, ultraviolet (UV), and infrared (IR) light.^29^ Product titers and conversion efficiencies remain constrained by the inherent limitations of photosynthetic energy capture and distribution, including competition between growth and production pathways.^30^ Moreover, energy is lost during oxygenation and photorespiration, and dissipated as heat.^31^ However, it provides inspiration for artificial, semiartificial and semibiological photosynthesis systems that aim to surpass nature’s constraints by integrating synthetic materials with biological components.^17,32,33^ The detailed introduction and comparison of natural photosynthesis, artificial photosynthesis, and semibiological photosynthesis are listed in Table 1.

**Table 1:** Introduction and Comparison of Natural Photosynthesis, Artificial Photosynthesis, and Semibiological Photosynthesis

category	natural photosynthesis	artificial photosynthesis	semibiological photosynthesis
definition	light-driven CO_2_ and H_2_O conversion into carbohydrates	mimicking photosynthes is mimicking natural photosynthesis using inorganic/organic semiconductors and dyes	integration of microbes with artificial materials for light-driven chemical conversion
energy source	sunlight	sunlight (photocatalysts, photoelectrodes)	sunlight
reaction systems	plants, algae, photosynthetic bacteria	semiconductors photocatalysts, photoelectrodes, molecular catalysts, etc.	hybrid systems integrating microbes with semiconductors/photoelectrodes
electron transfer pathways	biological electron transfertransport chains	charge separation and transfer via semiconductor junctions	electron transfer between abiotic and biotic components
reaction rates	low	high	moderate
main products	carbohydrates (e.g.,glucose) and biomass	H_2_, CO, CH_3_OH, CH_4_..., etc	H_2_, CH_4_, alcohols, C_2+_ products, etc
oxidation reaction	water oxidation producing O_2_	water oxidation or organic oxidation	water oxidation or organic oxidation
advantages	naturally evolved with high selectivity and long-term stability	diverse materials platform with tunable reaction pathways and products	combines biological selectivity with efficient light harvesting of artificial materials
disadvantages	low solar-to-chemical conversion efficiency	limited stability and selectivity	complex system design and challenges in scalability
typical examples	photosynthesis in green plants and cyanobacteria	TiO_2_, C_3_N_4_, and CdS for water splitting, CO_2_ reduction, and N_2_ fixation	microbe–semiconductor biohybrids (the focus of this review)

### 2.2 Artificial Photosynthesis

In artificial photosynthesis, synthetic light-harvesting semiconductor materials and catalysts are used to convert solar energy into chemical energy.^34–36^ The band structures of typical organic and inorganic semiconductor nanomaterials as well as PSI and PSII are summarized to illustrate the thermodynamic redox potentials (Figure 3).^37,38^ Inorganic semiconductor nanomaterials typically display superior electrical properties and higher stability compared to organic semiconductors, owing to their rigid and well-defined electronic structure. Since the electronic properties of a material are intrinsically linked to charge carrier mobility, inorganic semiconductors enable more rapid electron separation and transfer, thereby resulting in improved overall performance. In contrast, organic compounds offer great flexibility in molecular structure design, which allows for precise bandgap tailoring. The key design strategies in molecular structure engineering involve altering the backbone structure and introducing functional side chains to optimize the surface property and improve solution processability. Such easily tunable structural and optical properties as well as good biocompatibility also make organic semiconductors attractive candidates for integration with microbes to construct semibiological photosynthetic systems.

**Figure 3:**
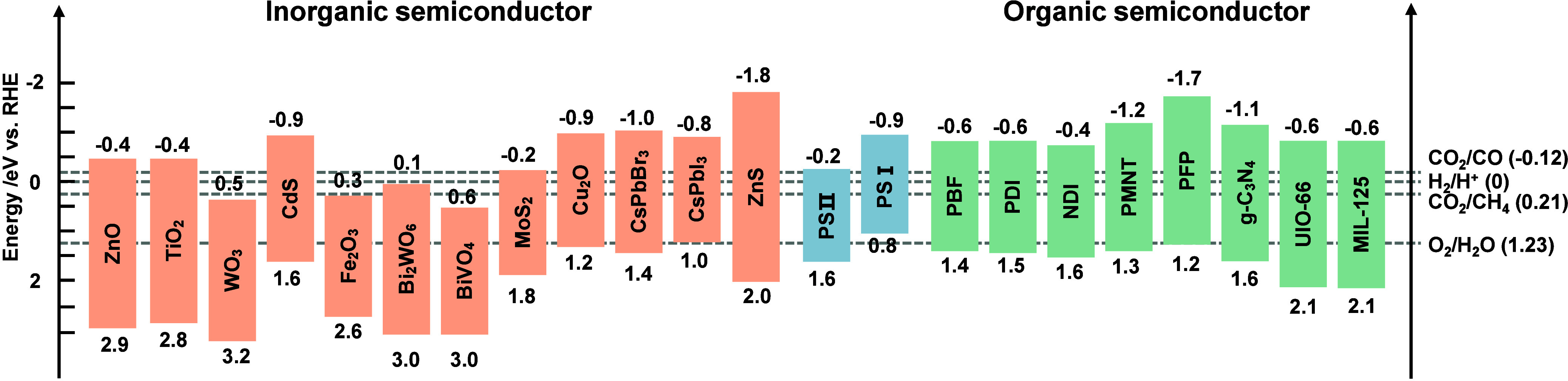
Energy band edges of typical inorganic and organic semiconductors. Reproduced with permission from^37^. Copyright 2025 Elsevier.

Generally, the bandgap determines the ability of a semiconductor material to absorb solar light and generate photoexcited electrons, both of which are crucial for photocatalysis. However, there is a trade-off between the light absorption range and the relevant redox potential. Semiconductors with narrow bandgaps can exhibit outstanding photon absorption abilities, leading to a broader utilization of the solar spectrum. Nevertheless, they possess relatively lower redox potentials to drive endergonic reactions. Conversely, a wide bandgap of more than 3.0 eV provides a greater redox driving force, yet the accessible range of light falls within the UV region.^39^

Photocatalytic reduction of small molecules using semiconductor-based catalysts generally proceeds through three fundamental steps (Figure 2b).^40^ First, when incident light with energy equal to or greater than the semiconductor’s bandgap is absorbed, electrons are excited from the valence band (VB) maximum to the conduction band (CB) minimum, generating electron–hole pairs.^37^ These photoexcited charge carriers then migrate to the semiconductor surface, where they participate in redox reactions that are typically aided by a cocatalyst. At the surface, the photogenerated electrons reduce adsorbed substrates such as H^+^, CO_2_, or N_2_, while the holes drive oxidation reactions, typically involving water or other abundant substrates to yield value-added products.^41^ For the overall process to be thermodynamically favorable, the CB minimum must lie at a potential that is more negative than that of the relevant reduction half-reaction, and the VB maximum must lie at a potential that is more positive than that of the corresponding oxidation half-reaction. In addition to desirable band alignment, fast reaction kinetics also requires appropriately engineered active sites to promote substrate activation and enable fast interfacial charge transfer.^42^

Currently, many strategies such as constructing bioinspired Z-scheme heterostructures (Figure 4a), loading single atom cocatalysts, and directly interfacing metal complex catalysts on semiconductors have been carried out to improve charge transfer and optimize catalytic selectivity for efficient artificial photosynthesis.^43–46^ For example, we have recently synthesized various Z-scheme heterostructures to optimize interfacial electron transfer and regulate catalytic active centers for efficient and selective CO_2_ photoreduction.^47–49^ Meanwhile, we have also conducted in-depth investigations into the underlying mechanism of Z-scheme formation in the artificially synthesized photocatalysts.^47,48^

**Figure 4:**
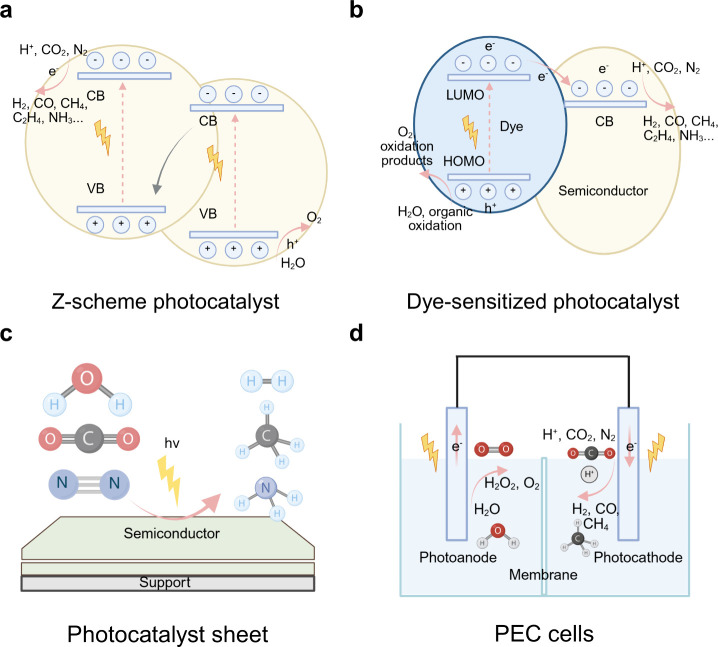
Fundamental mechanisms of typical photocatalytic systems and PEC cells. (a) Scheme of the Z-scheme photocatalyst. (b) Scheme of dye-sensitized photocatalyst. (c) Schematic illustration of the photocatalyst sheet. (d) Schematic illustration of PEC cells.

Dye-sensitized semiconductors (Figure 4b) allow the use of molecular dyes to tune visible light absorption in combination with wide-band semiconductors to improve photocatalytic performance, thus providing benefits to combine advantages of both homogeneous and heterogeneous photocatalysis.^50^ In fact, when aiming at anchoring molecular catalysts on semiconductor surfaces or immobilizing molecular catalysts onto PEC electrodes, many different aspects should be considered to improve the activity, stability, and selectivity, such as an anchoring group which allows the chemisorption of the molecular structure and a linker which ensures effective interfacial connection and electron transfer.^45,51,52^ These key interfacial designs between molecular catalysts and semiconductors/electrodes also offer us some useful ideas to build efficient biohybrid systems.^53–55^

Artificial photosynthesis systems are typically implemented in two main configurations. One approach involves dispersing light-absorbing semiconductor photocatalysts or constructing photocatalyst sheets (Figure 4c) in a photoreactor, while the other employs PEC cells,^56^ in which catalysts are immobilized on electrodes (Figure 4d).^57–60^ Generally, PEC cells exhibit higher STC conversion efficiency because PEC configurations employing separate photoanodes and photocathodes offer unique benefits for superior charge separation and catalytic reactions.^61^ However, from a cost-performance standpoint, photocatalytic systems are generally more favorable, as PEC systems often require solar-to-hydrogen conversion efficiencies approaching 25% to be economically competitive, whereas photocatalytic systems could be viable at efficiencies as low as 5–10%.^57,62^ Scale up attempts have been made to demonstrate actual applications of photocatalysts. Nishiyama et al. have designed a 100 m^2^ array of panel reactors based on a modified aluminum-doped strontium titanate particulate photocatalyst to operate for several months with autonomous recovery of H_2_.^63^ Photocatalyst sheets consisting of a metal complex catalyst immobilized on a Z-scheme semiconductor system for selective CO_2_ reduction to formate has recently been reported.^64^ Noble-metal-free carbon nitride/nickel phosphide (CN_*x*_/Ni_2_P) photocatalyst sheets are being developed for photoreforming of plastic, biomass, mixed waste into H_2_ and organic molecules.^65^

Besides, artificial leaves integrate the photoanode directly with the photocathode through a simple engineering configuration to directly drive water splitting or CO_2_ reduction without the use of wires.^66,67^ For instance, we have built perovskite-BiVO_4_ artificial leaves to achieve unassisted artificial photosynthesis to convert CO_2_ into syngas or multicarbon fuels.^68,69^ The leaf-shaped PEC device bridges the weight gap between traditional solar fuel approaches, with activity per gram comparable to photocatalytic suspensions and plant leaves.^68^ Scale-up of artificial leaf arrays known as an artificial tree is already approaching m^2^ scale, which has shown great promise for practical applications.^3,70–72^

Nowadays, a variety of photocatalytic reactions can be achieved by selecting a specific catalyst for the reaction of interest. A typical example is the light-driven overall water splitting to generate H_2_.^73,74^ Although research in solar water splitting has been ongoing for decades,^75^ several challenges remain unresolved to allow for commercial use. For example, the water oxidation half-reaction is kinetically sluggish and therefore requires a high overpotential, making overall water splitting difficult. Compared to electrocatalytic systems, photocatalytic systems currently have lower efficiency and higher cost, enabling it less attractive for industrial application.^76^

Beyond the prototypical water-splitting reaction to produce H_2_, artificial photosynthesis aims to convert CO_2_ into value-added products, offering a pathway to close the anthropogenic carbon cycle and enable carbon capture and utilization.^77^ However, CO_2_ is a thermodynamically stable molecule, and its activation, particularly the one-electron reduction to CO_2^•^_^–^ at −1.9 V vs SHE, is highly energy-intensive.^78^ The efficient reduction process therefore needs to proceed through controlled sequences of proton- and electron-transfer steps, and only a limited number of simple products are currently accessible. Moreover, the low solubility of CO_2_ in water and its competition with proton reduction to hydrogen further compromise the selectivity and overall efficiency. At present, photocatalytic CO_2_ reduction predominantly yields CO and formate with good selectivity, while the selective formation of multicarbon products such as ethanol or ethylene remains challenging and limits the economic viability of this approach.^46,79^

Artificial photosynthesis is also being explored as a sustainable route for nitrogen reduction. Unlike the conventional Haber-Bosch process, which requires high temperatures and pressures and emits substantial CO_2_ due to its dependence on hydrogen from fossil fuels,^80^ photocatalytic nitrogen fixation aims to produce ammonia under ambient conditions using only solar energy, air and water.^81,82^ This approach offers significant environmental benefits but remains at an early stage of development. Current systems exhibit very low solar-to-ammonia conversion efficiencies, typically below 1%.^83–85^ Furthermore, progress is hindered by technical challenges, including the accurate quantification of ammonia and the need to eliminate potential experimental artifacts.^86,87^

### 2.3 Microbial (Electro)­synthesis

Microbial synthesis refers to the use of microbes as cell factories to convert renewable feedstock, such as CO_2_ and biomass-derived substrates into target products through native or engineered metabolic pathways.^88^ Over the past decades, metabolic engineering has greatly expanded the scope of microbial synthesis, enabling the production of valuable alcohols, organic acids, polymers, and even complex pharmaceuticals. The advantages of microbial synthesis mainly include mild operating conditions and an inherent metabolic specificity. However, conventional microbial synthesis is limited by the energy demands of cellular metabolism and the unfavorable efficiency of converting substrates into reduced products.^89^

To address these challenges, microbial electrosynthesis (MES) has been developed as an approach that couples microbial metabolism with electrochemical energy input (Figure 5).^90^ In MES, microbes are interfaced with a cathode that delivers electrons for the intracellular metabolism. For example, the acetogenic bacteria such as *Sporomusa ovata* (*S. ovata*) and *Moorella thermoacetica* (*M. thermoacetica*) have been commonly used for CO_2_ fixation and chemical synthesis. This process effectively bypasses the need for organic feedstocks, using CO_2_ as the sole carbon source and electrical energy as the reducing power. Various valuable products have been achieved from MES, including acetate, succinate, butanol, and longer-chain compounds, which are important building blocks for fuels and chemicals.^91,92^ Yang et al. have employed electricity to reduce flavin secreted by *S. oneidensis* at a standard redox potential of −0.32 V vs NHE^93^ for a 15.5-fold increase in the inward current output.^94^ As reported by us, it is also feasible to apply electricity to *S. oneidensis*-pNDI biohybrids to continuously synthesize succinate from fumarate.^95^ Other conventional redox reactions participating in biosynthetic processes include the reduction of microbial surface proteins (e.g., outer membrane cytochromes, approximately at −0.15 V vs Ag/AgCl) and the redox cycling of other energy carriers.^37^

**Figure 5:**
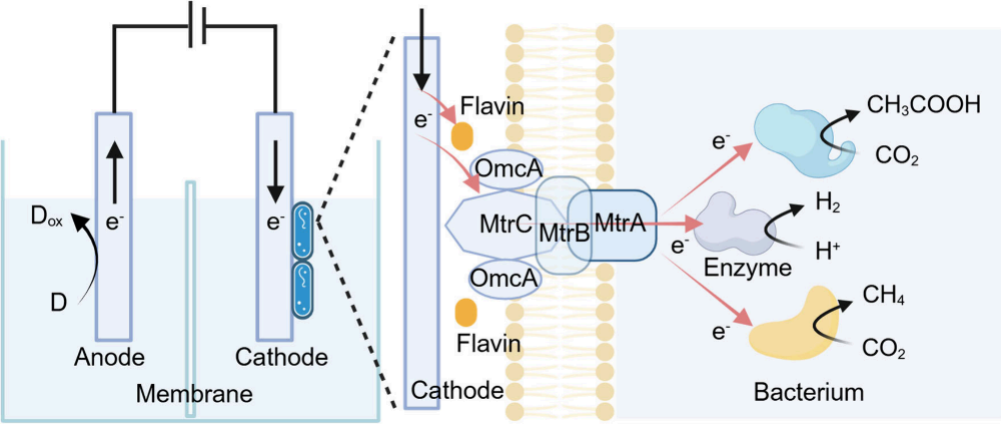
Microbial electrosynthesis: biotic–abiotic hybrid systems for inward EET process toward microbial electrosynthesis.

Engineering electrodes with enhanced conductivity, surface area, and biocompatibility has proven critical for improving electron transfer efficiency.^96,97^ For example, we have reported a three-dimensional (3D) electrode scaffold to couple the intracellular metabolism of *Geobacter sulfurreducens* (*G. sulfurreducens*) with extracellular redox transformations.^96^ The large population of *G. sulfurreducens* within this 3D electrode scaffold produces a benchmark current density of 3 mA cm^–2^ arising from microbial metabolism. At the same time, advances in genetic engineering of acetogens are expanding their product spectrum beyond simple C_1_ and C_2_ compounds toward higher-value chemicals.^98^

Notably, the MES system provides a good platform to probe the biotic–abiotic interfacial electron transfer, helping us understand how microbes interact with abiotic electrode surfaces.^99^ For example, some key electrochemical performance metrics offer real-time quantitative characterizations of electron fluxes between microbes and electrodes.^100^ Current density (mA cm^–2^) reflects the electron transfer rate per unit electron area and Faradaic efficiency (%) quantifies the ratio of injecting electrons contributing to yielding a specific product.^101^ Electrochemical charge transfer resistance can unravel the electron transfer dynamics at the biotic–abiotic interfaces.^102^ These electrochemical approaches enable detailed analysis of microbial redox pathways, narrowing the gap between electrochemical results and metabolic activity at the biohybrid interfaces.^103^ In summary, the microbial electrosynthesis represents an innovative extension that directly couples microbial metabolism with energy conversion and chemical synthesis.^104,105^ These useful strategies of optimizing electrode materials and integrating synthetic biology as well as interfacial characterization techniques can also provide us with insightful directions for semibiological photosynthesis,^106^ which is the key focus of this review.

### 2.4 Microbe–Semiconductor Biohybrids for Semibiological Photosynthesis

Semibiological photosynthesis is an emerging approach to overcome the limitations of natural and artificial photosynthesis (Figure 2c).^107^ Semiconductors can be tuned to optimize the absorption of the solar spectrum,^4^ and are generally more stable than PSII, which requires repair approximately every 30 min due to photodamage.^108^ In addition, semiconductors can enable photoreforming processes by oxidizing waste organic materials, such as biomass and plastics, as electron donors to produce value-added chemicals rather than O_2_.^109–112^ The photoexcited electrons generated by irradiation of the semiconductor will be transferred to microorganisms which catalyze the reduction of CO_2_, N_2_ or water to chemicals.

Microbes are powerful and efficient biocatalysts because they employ confined enzyme cascades to drive chemically complex reactions to generate metabolites, which can be used as biofuels and chemicals. For example, microbes can reduce CO_2_ to produce multicarbon products with high selectivity and have the potential for long-term operation due to their inherent ability to repair and replicate. This also means that the chemical production efficiency is often lower than 100% since some of the carbon and electron flux in the metabolic pathway is directed toward growth and repair. Nevertheless, microbes offer many opportunities for catalyzing the CO_2_ reaction, such as acetogenic bacteria which reduce CO_2_ to acetate via the acetyl-CoA pathway. Due to the low thermodynamic operating limit of this pathway, the acetyl-CoA pathway is the most energy efficient biological CO_2_ fixation pathway.^113–116^ Furthermore, synthetic biology can also be employed to target non-native higher value products.^117^

The integrated semibiological photosynthesis systems reported in the literature typically include four different configurations (Figure 6): dispersed colloidal semiconductor nanoparticle–microbe biohybrid system, semiconductor photocatalyst sheet–microbe biohybrid system, microbe–semiconductor PEC biohybrid system, and photovoltaic (PV) device powered electrochemical (EC)–microbe biohybrid system. In the case of colloidal semiconductor photocatalytic systems, semiconductor nanoparticles harvest light and generate electron–hole pairs. The hole will be quenched by a sacrificial electron donor, commonly cysteine, or water oxidation, and the excited electron will be transferred to the microbe via direct electron transfer (DET) or to a mediator such as hydrogen versus mediated electron transfer (MET). For example, Sakimoto et al. used CdS nanoparticles to directly interface with acetogenic *M. thermoacetica* to catalyze the reduction of CO_2_ to acetate.^118^ A similar strategy was employed by Ye et al. to combine CdS photocatalytic colloidal system with methanogenic bacteria *Methanosarcina barkeri* (*M. barkeri*) to catalyze the conversion of CO_2_ to methane.^119^ By precipitation of the light absorber on the surface of the bacteria, the photoexcited electrons were transferred from the light absorber to the membrane-bound electron transport cytochrome proteins or hydrogenase without the participation of diffusional mediators.

**Figure 6:**
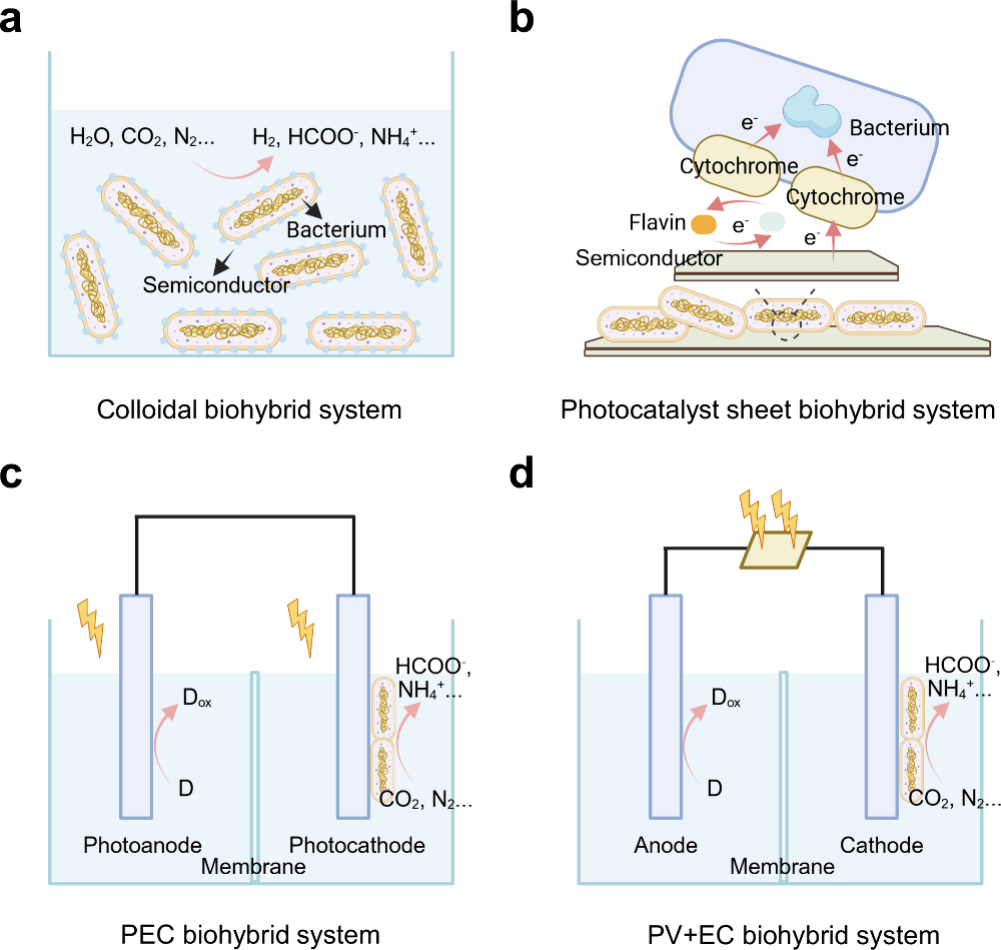
Schematic illustration of integrated microbe–semiconductor biohybrid systems. (a) Dispersed colloidal semiconductor–microbe biohybrid system. (b) Semiconductor photocatalyst sheet–microbe biohybrid system. (c) Microbe–semiconductor PEC biohybrid system. (d) Microbe–semiconductor “PV+EC” biohybrid system.

In both systems, cysteine was used as the sacrificial electron donor, which limited the longevity of the system since the reaction would cease once the sacrificial electron donor was used up. This issue was addressed in follow-up work by Sakimoto et al., who coupled an existing biohybrid with a manganese phthalocyanine/titania photocatalyst that oxidized water and reduced cystine, regenerating the sacrificial cysteine electron donor (Figure 7a).^120,121^ Note that Göbbels et al. have reported that cysteine can also be metabolized to acetate using a CdS–*M. thermoacetica* biohybrid, and this metabolic pathway occurs independently of light and CdS.^122^ Therefore, further investigation is needed to clarify the carbon source of acetate production, and the use of cysteine should be avoided in such semibiological photosynthesis experiments.

**Figure 7:**
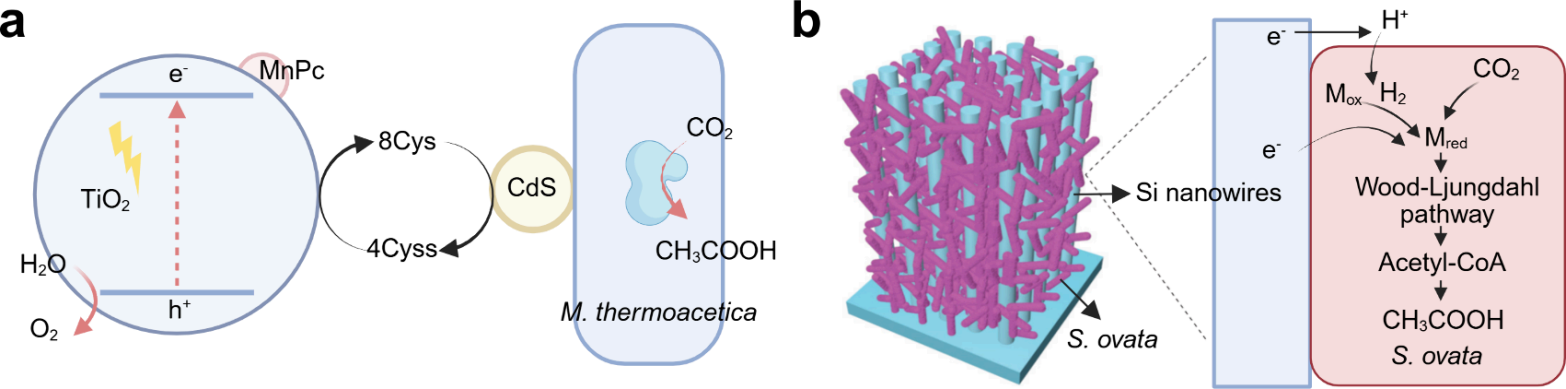
Typical microbe–semiconductor biohybrid systems using colloidal and PEC systems. (a) Scheme of the CdS–*M. thermoacetica* + TiO_2_–MnPc colloidal system. (b) Schematic illustration of the Si nanowire–*S. ovata* PEC system and microbial metabolic pathway. Reproduced with permission from ref 120. Copyright 2016 American Chemical Society. Reproduced with permission from^128^. Copyright 2020 Elsevier.

Another configuration is the use of PEC devices (Figure 6c) or PV powered EC devices (Figure 6d) in microbe–semiconductor biohybrid systems.^123,124^ In this case, the oxidation and reduction reactions are carried out on separate electrodes in separate compartments, but the overall electron transfer chain remains the same, i.e., extracting electrons from water or an abundantly available biomass-derived alcohols with a light absorber to transfer reducing power to microbes which catalyze the reduction of CO_2_.^125^ For example, Nichols et al. used a p-type indium phosphide photocathode to catalyze the H_2_ evolution reaction, with *M. barkeri* in the catholyte using H_2_ and CO_2_ to produce CH_4_, while the O_2_ production from water oxidation occurs at photoanode.^126^ Another typical PEC example is the use of silicon as a light absorber and scaffold to support the growth of *S. ovata* for CO_2_-to-acetate conversion (Figure 7b).^127,128^ Higher value products can be reached by genetically engineering the bacteria, for example *Cupriavidus necator* (*C. necator*) (a.k.a. *Ralstonia eutropha*, *R. eutropha*) has been engineered to produce a range of products such as 2-propanol, isobutanol, poly­(3-hydroxybutyrate), 3-methyl-1-butanol from CO_2_ and H_2_.^129^

Apart from these integrated biohybrid systems, separate biohybrid systems can also be utilized in semibiological photosynthesis (Figure 8): “photocatalysis + fermenter” biohybrid system, “photocatalyst sheet + fermenter” biohybrid system, “PEC + fermenter” biohybrid system, and “PV + EC + fermenter” biohybrid system. By decoupling the (photo)­electrochemical reactions from microbial synthesis, these separate systems avoid direct microbe–semiconductor interactions, which can improve operational stability and minimize biological stress. For example, we reported a coupled abiotic–biotic system by integrating a photocatalytic system with a fermenter, which connected a photocatalytic CO_2_-to-syngas (a mixture of CO_2_, H_2_, and CO) reaction with evolved syngas-fermenting *Clostridium ljungdahlii* (*C. ljungdahlii*) to achieve CO_2_ conversion into valuable C_2+_ products.^130^ In this system, a cobalt terpyridine derivative molecular catalyst immobilized on titania was used to produce syngas as the energy and carbon source for *C. ljungdahlii* toward the synthesis of acetate and ethanol. Besides, the “PV+EC” system coupled with a fermenter has also been designed. Haas et al. employed a modular system whereby a PV device was connected to an electrolyzer to generate syngas from water and CO_2_. The syngas was fed into a fermenter and converted to acetate and ethanol with *Clostridium autoethanogenum*. The acetate and ethanol was then converted to butyrate and hexanoate by *Clostridium kluyveri*.^131^ This is a demonstration of a modular multistep device where each component is connected in flow and can operate under optimal conditions. Notably, this research is supported by Evonik and Siemens, which plan to produce high-value chemicals from CO_2_ and eco-electricity.

**Figure 8:**
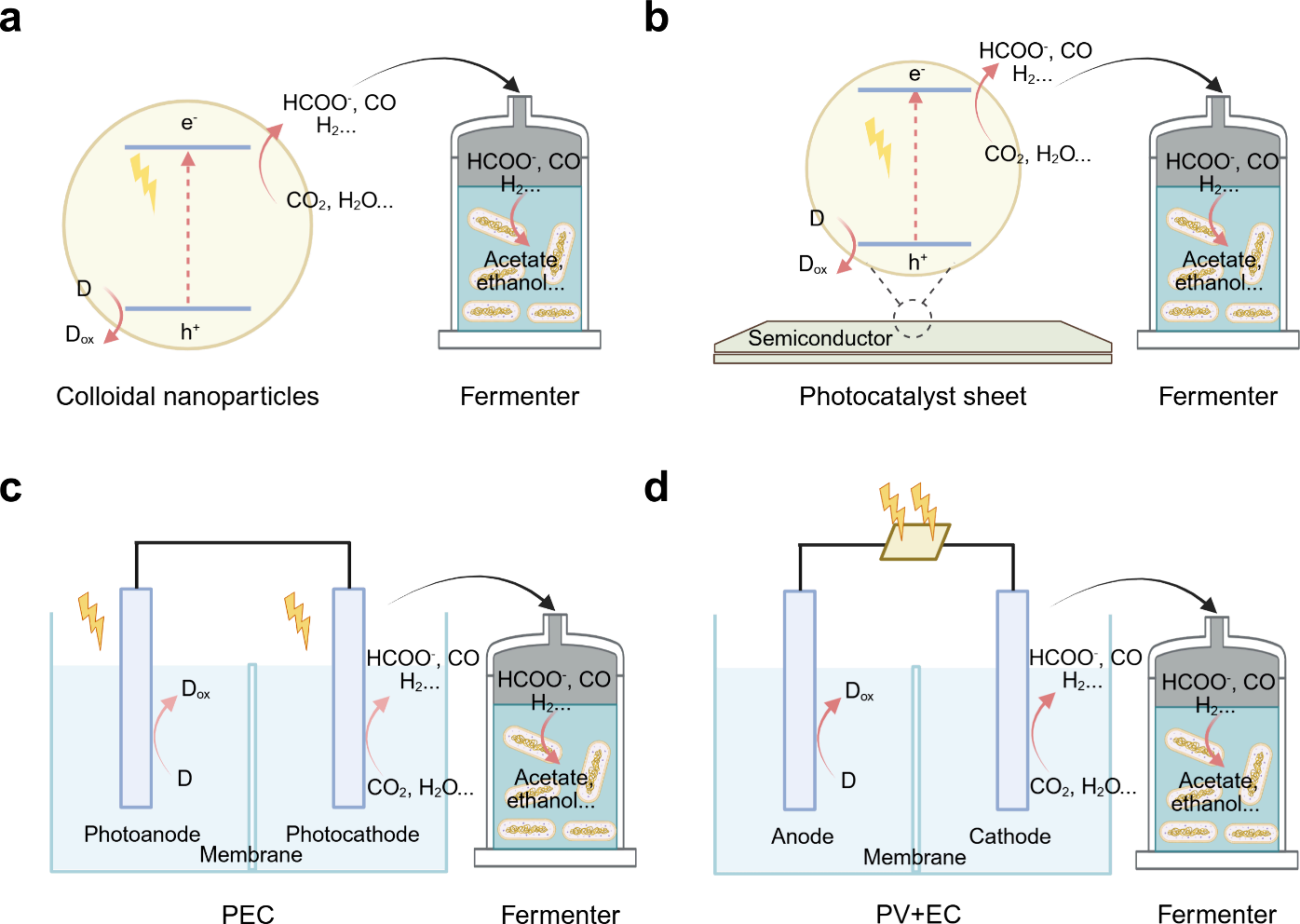
Schematic illustrations of separate biohybrid systems: (a) “Photocatalysis + fermenter” biohybrid system. (b) “Photocatalyst sheet + fermenter” biohybrid system. (c) “PEC + fermenter” biohybrid system. (d) “PV + EC + fermenter” biohybrid system.

Considering the complexity and expense of separate biohybrid systems, integrating the photocatalytic systems and PEC devices with microbes is a feasible and economic way to achieve semibiological photosynthesis for STC conversion. Therefore, integrated PEC biohybrid systems and photocatalytic biohybrid systems have been extensively employed to demonstrate the concept of microbe–semiconductor biohybrids for solar-driven biosynthesis. Such integrated biohybrid systems further require us to focus on the design of microbe–semiconductor biohybrid interfaces.^132^

## 3 Fundamentals of Microbe–Semiconductor Interfacial Electron Transfer

### 3.1 Typical Microbes and Extracellular Electron Transfer Pathways

Electroactive microorganisms (EAMs), recognized for their ability to exchange electrons with external materials, are the foundational biological modules in the design of a solar-driven biosynthetic system. The modes of EET have been deciphered and classified into DET and MET pathways.^92^ Specifically, the DET process is established via outer membrane proteins to create cell-to-surface contact.^133^ It has been suggested that direct physical contact within 14 Å is required for electron tunnelling to occur effectively.^134^ Although this approach ensures a more robust electrochemical interaction at the interface, its rate is restricted by the short tunneling distance and limited surface area where a material can implement an effective physical contact. To achieve good interfacial interactions, electroactive microbes may evolve conductive surface appendages that extend several micrometers from the cell surface, enabling electrons to traverse the physical boundaries and even facilitating electron flow within microbial communities.

The MET pathway is considered complementary to promote long-range electron transport through the secretion of soluble redox mediators, such as flavin, phenazines, and quinones, which can diffuse freely for electron exchange activities. Not only do these redox shuttles interact with designated proteins but also they act as bound cofactors buried within enzyme-active sites and specific proteins, thereby activating catalytic redox reactions and increasing the electron turnover rate. From a thermodynamic perspective, the redox potential of a mediator should differ from that of the target enzyme by at least 50 mV to ensure a spontaneous reaction.^135^ Several studies have reported that artificial redox mediators, such as neutral red (NR) and metal complexes, such as ferrocene, can bind to a diverse range of redox proteins in EAMs. The introduction of synthetic and biocompatible mediators into microbial electrochemical systems has been further demonstrated to significantly enhance the system performance. Among the various electroactive bacteria, the Gram-negative and mesophilic *Shewanella oneidensis* (*S. oneidensis*) MR-1 and *G. sulfurreducens* are the most well-characterized model exoelectrogens. Besides, some acetogenic bacteria such as *S. ovata* and *M. thermoacetica* performing biochemical pathways also receive much interest. The key features of some typical bacteria in microbial synthesis are listed and compared in Table 2.

**Table 2:** Summary and Comparison of Typical Microbes in Biohybrid Systems

microbial species	type	electron transfer pathway	main products	application
*S. oneidensis*	facultative anaerobe, metal-reducing bacterium, electroactive bacterium	DET via cytochrome C and MET via flavins	electricity, H_2_, reduced metals	microbial electrosynthesis; semibiological photosynthesis; microbial fuel cells
*G. sulfurreducens*	obligate anaerobe, metal-reducing bacterium, electroactive bacterium	DET via cytochrome C and conductive pili (nanowires)	electricity, succinate	microbial electrosynthesis, microbial fuel cells
*S. ovata*	obligated anaerobe, acetogen	MET via H_2_; accepting electrons from cathodes or semiconductor	acetate from CO_2_ (Wood–Ljungdahl)	microbial electrosynthesis; semibiological photosynthesis
*M. thermoacetica*	obligate anaerobe, thermophilic acetogen	MET via H_2_; accepting electrons from cathodes or semiconductor	acetate from CO_2_ (Wood–Ljungdahl)	semibiological photosynthesis
*C. ljungdahlii*	obligate anaerobe, acetogen	MET via H_2_ or CO	acetate/ethanol from CO_2_ (Wood–Ljungdahl)	microbial electrosynthesis; semibiological photosynthesis
*A. vinelandii*	aerobe (N_2_ fixation)	MET via mediators	N_2_ fixation (Nitrogenases)	semibiological photosynthesis
*A. chroococcum*	aerobe (N_2_ fixation)	MET via mediators	N_2_ fixation (Nitrogenases)	semibiological photosynthesis
*E. coli*	facultative anaerobe	MET via mediators	electricity, H_2_ (hydrogenases), chemicals	microbial fuel cells, microbial electrosynthesis, semibiological photosynthesis

*S. oneidensis*, as a dissimilatory metal-reducing (Mtr) bacterium,^136^ is naturally endowed with an EET system (Figure 9a).^137^ This EET system comprises the concerted action of multiheme c-type cytochromes (c-Cyts) that allow electrons to relay across membranes. In essence, five proteins have been identified as being responsible for electron transfer. The c-type cytochrome A (CymA), located on the exterior of the cytoplasmic membrane, functions as a gateway for free electrons to shuttle between the quinone/quinol pool and the periplasm. The released electrons from CymA are transferred to the periplasmic redox-active proteins, such as fumarate reductase (FccA)^138^ and small tetraheme cytochromes. In the presence of exogenous electron acceptors like minerals and electrodes, intracellular electrons can be transferred from CymA into the Mtr respiratory pathway via periplasmic c-type cytochromes.^139^ The Mtr pathway is composed of the MtrA, MtrB and MtrC proteins, which is partially embedded in the outer membrane and protrudes into the extracellular space.^98^

**Figure 9:**
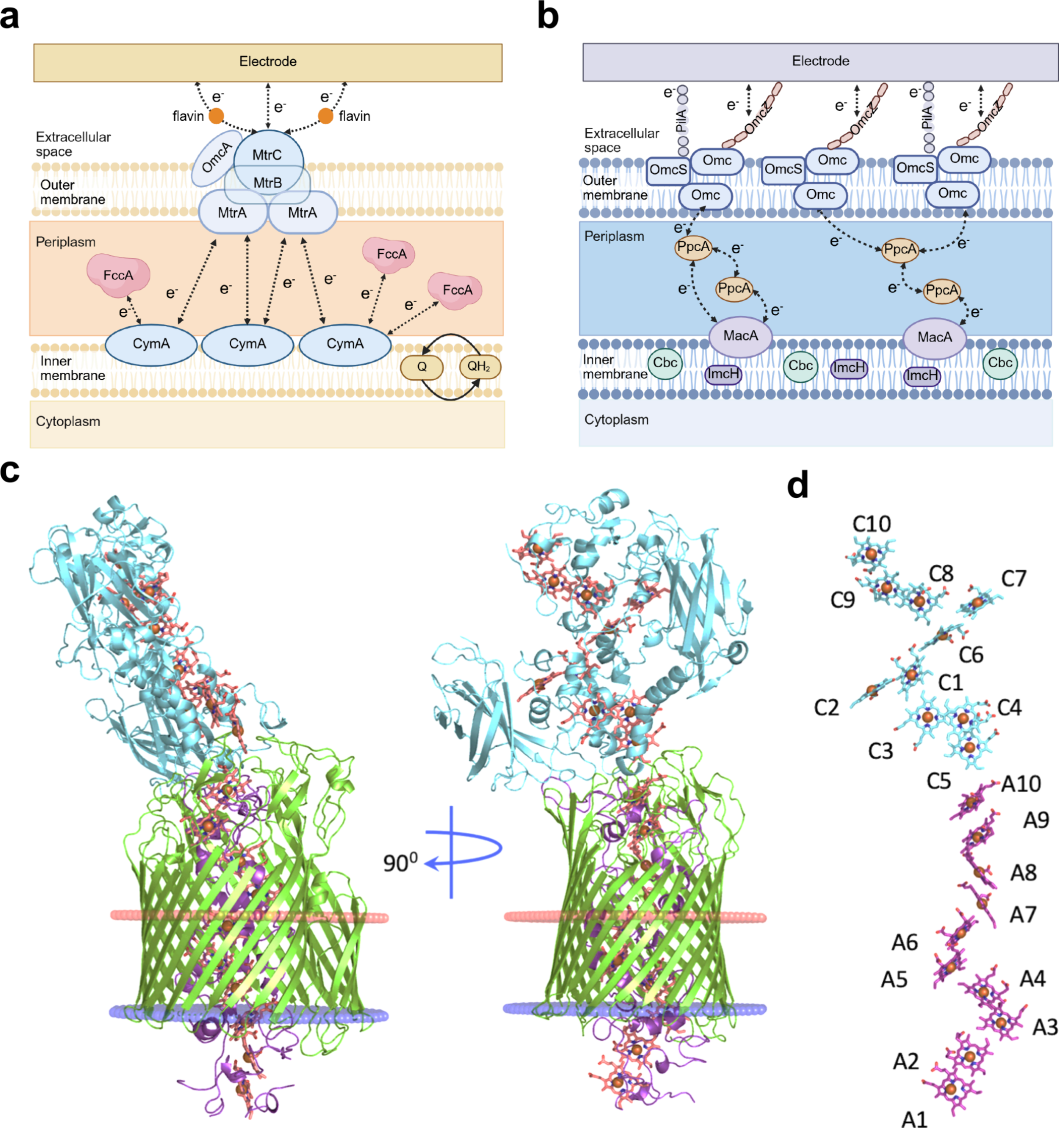
Detailed EET pathways in typical electroactive bacteria. (a) EET pathway in *S. oneidensis*. (b) EET pathway in *G. sulfurreducens*. (c) X-ray crystal structure of the Mtr complex from *S. baltica OS185*. Cartoon views of the Mtr complex rotated by 90 deg. The extracellular MtrC (blue) is connected to the surface of MtrB (green). MtrA (magenta) is located inside MtrB. The lipid bilayer positions of the Mtr complex are shown as red and blue disks, representing extracellular and periplasmic surfaces, respectively. (d) Heme chains within the Mtr complex. Hemes are numbered based on the position of the heme attachment motif within the amino acid chain and colored blue (MtrC) or magenta (MtrA). Reproduced with permission from ref 92. Copyright 2017 Springer Nature. Reproduced with permission from ref 143. Copyright 2020 Elsevier.

Outer membrane cytochrome A (OmcA), another decaheme c-type cytochrome protein discovered in most electroactive microbes, particularly in *S. oneidensis* MR-1, works in tandem with MtrC to modulate electron flow toward terminal redox species.^92^ Moreover, MtrC and OmcA, ranging in size from 80 to 90 kDa, contain extracellular flavin-binding sites that enable interaction with small-molecule redox shuttles.^93^ These redox mediators, including riboflavin (RF) and flavin mononucleotide (FMN), will bridge the spatial gap between cells and external electron acceptors, leading to enhanced efficiency of interfacial extracellular electron transfer. In the case of indirect electron transfer, flavins can also act as cofactors that are tightly coupled to OmcA and are positioned in a specific orientation relative to the surrounding protein matrix. This precise arrangement allows their reactivity and electronic properties to be fine-tuned by the local environment.^93^ Additionally, the molecular makeup and conductivities of microbial nanowires from *S. oneidensis* have attracted considerable interest due to their roles in the long-range electron transfer process. Pirbadian and co-workers first observed the formation of nanowires in *S. oneidensis* in vivo. Based on this, they discovered that the composition of these membrane extensions consists of multiheme cytochromes rather than pilin proteins.^140^ Another research study has verified the contribution made by bacterial nanowires in mediating micrometer-scale electron transfer, achieving a rate of up to 10^9^ s^–1^ under an applied bias of 100 mV.^141^

Although the Mtr complex from *S. oneidensis* cannot be crystallized,^142^ different *Shewanella* species endogenously produce homologous Mtr complexes, and the Mtr complex from *Shewanella baltica OS185* (*S. baltica OS185*) yielded a single crystal (Figure 9c).^143^ The X-ray crystal structure shows that the Mtr complex consists of two cytochromes (MtrA and MtrC) and a beta-barrel protein (MtrB). A network of 20 bis-His coordinated heme groups spans the whole complex (Figure 9d), forming an electron transfer pathway of 185 Å that evolved to overcome the physical barrier of the outer membrane while sustaining efficient interfacial electron flow. The MtrA is a decaheme c-type cytochrome located in the periplasm. It contains a chain of ten c-type hemes arranged in a linear fashion, with edge-to-edge distances between 3.9 to 6.5 Å.^143^ This feature allows fast electron tunneling along the MtrA chain and builds a conductive heme wire to bridge the periplasmic electron pool to the outer membrane. MtrB, is a beta-barrel protein spanning the outer membrane, which acts as a scaffold and insulating sheath to encapsulate and stabilize MtrA.^144^ The barrel-shaped geometry of MtrB ensures the correct positioning and protection of MtrA, providing a dedicated conduit for long-distance electron transport. MtrC is also a decaheme c-type cytochrome and anchored to the outer membrane surface.^143^ Like MtrA, MtrC also consists of a chain of ten c-type hemes for directional electron transfer. X-ray crystal structure shows that MtrC is directly connected with the terminal heme A10 of MtrA, which enables a continuous electron transfer pathway across the whole membrane.^145^ Besides, the surface-exposed hemes of MtrC can interact with insoluble electron acceptors, achieving an effective interaction between intracellular metabolism and extracellular redox processes. Overall, this structural elucidation shows that the Mtr complex is an efficient electron conduit, which not only provides a mechanistic basis for the EET process but also serves as a model system to help us engineer bioelectronic interfaces and bioinspired structures for sustainable energy conversion.

Extensive research has also been conducted to elucidate the modular electron transfer system of *G. sulfurreducens* (Figure 9b), which is involved in extracellular electron transfer. Located on the inner membrane (IM), redox-active proteins with cytochrome domains, like cytochrome bc (Cbc) complexes and inner membrane cytochrome H (ImcH), play a critical role in relaying electrons via membrane-associated cytochrome A (MacA) to the periplasm.^98^ Despite the functional similarity between these two IM proteins, previous studies have clarified that they operate in distinct electron transfer pathways; Cbc complexes will reduce electron acceptors only when the reduction potential is at or below −100 mV, whereas lmcH requires a potential above this threshold.^146^ The subsequent electron transfer from the periplasm to the cell envelope is mediated by a transmembrane module comprising periplasmic c-type cytochrome A (PpcA) homologues. Notably, the outer membrane hosts a wide array of c-type cytochromes to enhance direct electron exchange between the cell and external electron acceptors, including metal oxides and electrodes. Omc, as one of the key cytochromes, has functions analogous to those of MtrABC in *S. oneidensis*. Interestingly, *G. sulfurreducens* biofilms frequently engage in long-range electron transfer (LET) through the expression of electrically conductive microbial nanowires. These nanowires, known as e-pili, are type IV pili assembled from PilA protein subunits. It has been reported that the electronic conductivities of pilin nanofilaments can approach 5 mS cm^–1^,^147^ exhibiting characteristics similar to those of synthetic metallic nanomaterials. The underlying mechanism behind this semiconductive behavior was suggested to involve the π-π interaction between the amino acids, the biological building blocks of pili.^147^ However, the subject matter of the molecular composition and the fundamental mechanism of electron transfer in bacterial nanowires remain controversial.

Multiple studies have proposed that the OmcS filament also contributes significantly to the current generation in LET process.^148^ OmcS features seamlessly stacked multiheme cytochromes with an interheme distance of less than 3–6 Å,^149^ providing continuous redox-active sites for electron hopping between pili.^148^ This perspective was challenged by Ueki’s work, which revealed that the deletion of OmcS did not impair the electron transfer capability of *G. sulfurreducens* for thick biofilms.^146^ Liu and co-workers further demonstrated the dominant role of PilA monomers in e-pili conductivity, as evidenced by the PilA mutant strain having a more pronounced drop in electron transport performance than the ΔOmcS strain.^150^ OmcZ filament, as an alternative cytochrome-based nanowire, has been widely reported for its conductivity, almost 1000 times higher than that of OmcS.^149^ Despite the superior conductivity, the direct engagement of OmcZ in the LET appears to be limited. Nevertheless, OmcZ is more effective in promoting biofilm formation, which in turn enhances the overall system’s conductivity.

Apart from the cytochrome-dependent DET pathway observed in the model electroactive microbes, acetogenic bacteria typically rely on H_2_-mediated MET process that is tightly coupled to central metabolic pathways, particularly the Wood–Ljungdahl pathway.^151,152^ A characteristic species is *S. ovata*, known for its MET via H_2_ (Figure 10a). Abundant hydrogenases oxidize H_2_ to generate reducing equivalents,^153^ which are then fed into the Wood–Ljungdahl pathway for acetate production.^154^ Nevin et al. have suggested a DET pathway in *S. ovata*, as the cells can produce organic compounds via the direct supply of electricity.^155^ Nonetheless, more concrete evidence is required to confirm the presence of proteins responsible for DET in *S. ovata*. Concurrently, there has been evidence that *S. ovata* utilizes *Sporomusa* type ferredoxin-dependent transhydrogenase (Stn) conduits to transport electrons. The study conducted by Kumar and co-workers discovered the role of the Stn complex in the electron transfer chain of *S. ovata*. The Stn complex, composed of three subunits–StnA, StnB, and StnC, is a cytoplasmic enzyme that connects various cellular energy carriers. The typical energy carriers include NADH, NADPH, and ferredoxin.^153^ The distinctive characteristic of the Stn complex is electron bifurcation, which enables the partitioning of electron flow to drive spontaneous catabolic reactions while simultaneously conserving energy to power anabolic activities in the cells.^156^ Specifically, the catabolic reactions entail the use of StnC to facilitate electron production from the oxidation of NADPH. For anabolic metabolism, StnA and StnB serve as platforms for fixing CO_2_ into organic chemicals through the reduction of NAD^+^ and NADP^+^, utilizing reduced ferredoxin as the electron donor.^153^ This electron transfer process is further coupled with the rhodobacter nitrogen fixation (Rnf) complex and energy-converting hydrogenase (Ech) complexes, located in the cytoplasmic membrane of *S. ovata*, to facilitate the exchange of free ions across the membrane for ATP synthesis.^153^ These reactions enable cells to efficiently relay electrons, supporting both electron transfer and biosynthetic metabolism.^156^

**Figure 10:**
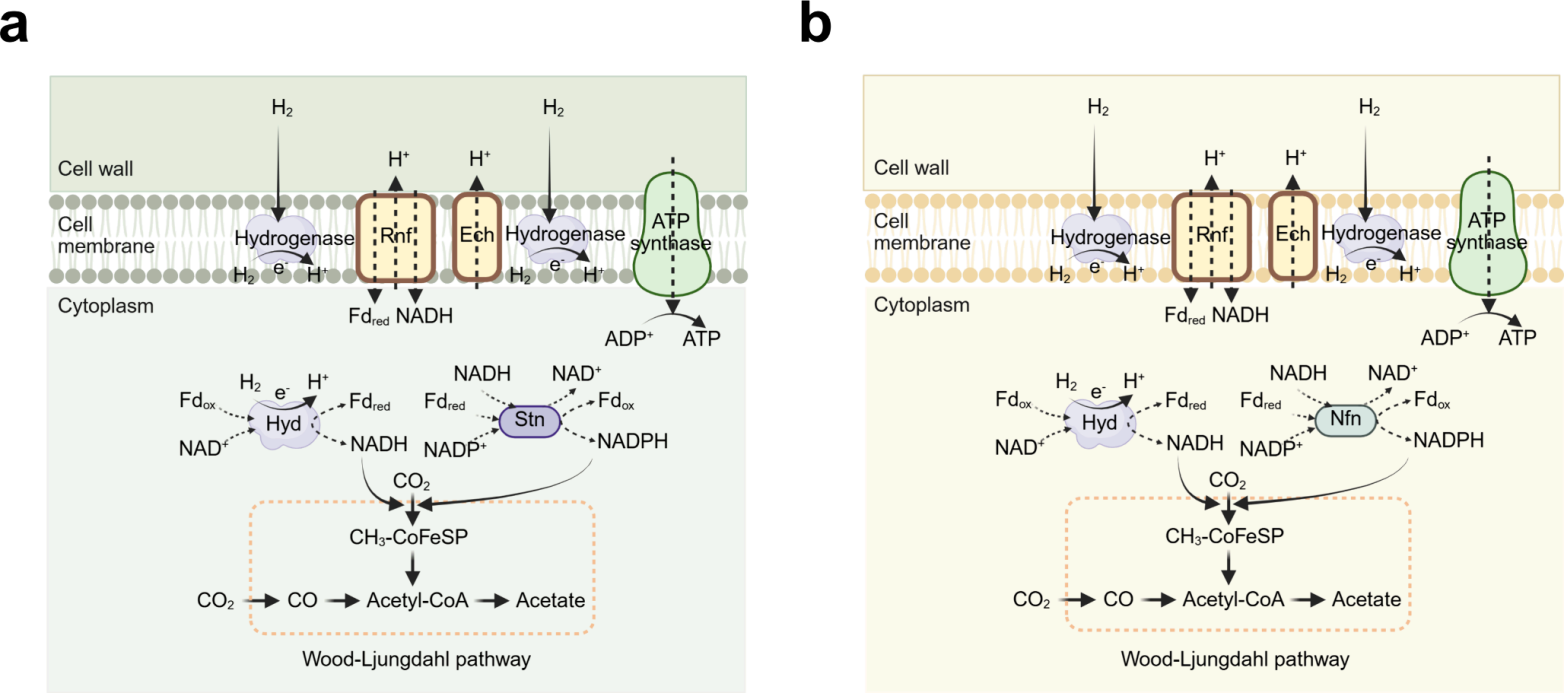
Detailed biochemical pathways in typical acetogenic bacteria. (a) Biochemical pathway in *S. ovata*. (b) Biochemical pathway in *M. thermoacetica*. Hyd, [FeFe]-hydrogenase-based electron-bifurcating complexes; Stn, *Sporomusa* type ferredoxin-dependent transhydrogenase; Nfn, NADH-dependent ferredoxin:NADP^+^ oxidoreductase; Ech, energy-converting hydrogenase; Rnf, rhodobacter nitrogen fixation complex. Reproduced with permission from ref 153. Copyright 2022 Frontiers.

*M. thermoacetica* (Figure 10b) also displays electron transfer mechanisms similar to those of *S. ovata*,^157^ centered around the Wood–Ljungdahl pathway and near-unity selectivity for acetate production; yet they differ in the redox protein complexes employed.^158,159^ *M. thermoacetica* relies primarily on the electron-bifurcating NADH-dependent ferredoxin:NADP^+^ oxidoreductase (Nfn) complex to balance NADH, NADPH, and ferredoxin pools, which favor a H_2_-mediated electron supply. In semibiological biohybrid systems, electrons from semiconductors or electrodes are first converted to H_2_, which then serves as a diffusive mediator for CO_2_ fixation.

In short, through these unique metabolic activities in different bacteria, various valuable chemicals and fuels can be obtained with high selectivity, such as hydrogen, acetate, and methane. Notably, even though a variety of electroactive and acetogenic bacteria have been investigated and applied in semibiological photosynthesis, the solar-driven biosynthetic system remains in the early stage of development. Further research efforts can be made to characterize and optimize biohybrid systems to yield more valuable chemicals and biofuels.

### 3.2 Bidirectional Pathways at the Biohybrid Interfaces

Bidirectional electron transfer capability is an intrinsic property of specific electroactive microbes that enables metabolic flexibility for multifunctional applications. These bacteria can alternate between electron donation and uptake by adjusting the direction of electron flow, either inward for biosynthesis or outward for bioelectricity, depending on the local environment. Generally, outward electron transfer involves the intracellular oxidation of organic substrates, releasing electrons that are subsequently transferred to an external electron-accepting electrode or semiconductor. This process can generate electricity in bioelectrochemical systems known as microbial fuel cells (MFCs) (Figure 11a).^160^ Organic substrates, including lactate, glucose, acetate, lactose, xylose, and certain organic acids, serve as energy sources for microbes to maintain basic cellular activities. When the direction of electron flow is reversed, moving from an exogenous electron donorinto the cell, the injected electrons can catalyze metabolic reduction to facilitate CO_2_ bioreduction or synthesize different commodity chemicals (Figure 11b), such as acetate and other renewable biofuels. Generally, inward electron transport is not thermodynamically spontaneous; therefore, it needs a more negative potential to create a driving force for biosynthesis. Since the bidirectional electron transfer holds great potential in expanding the functionality and versatility of biosynthetic systems, a comprehensive understanding of microbial species and their metabolic pathway mechanisms is essential to optimize system performance.

**Figure 11:**
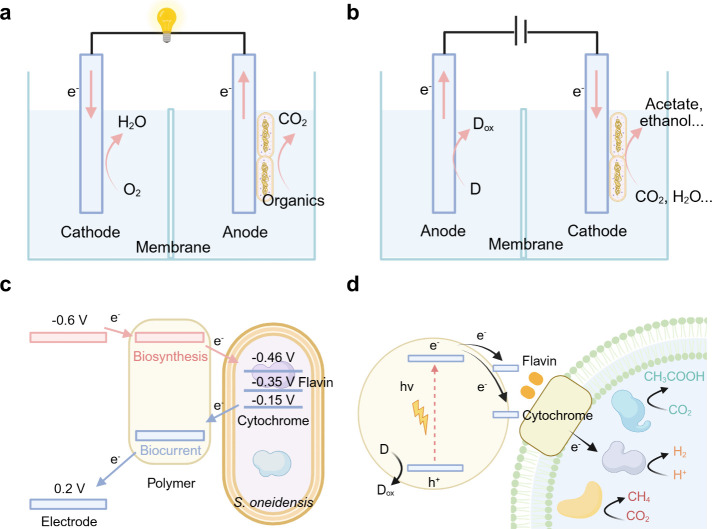
Bidirectional electron transfer for microbial fuel cells (biocurrent) and microbial electrosynthesis (biosynthesis). (a) Outward EET for microbial fuel cells. (b) Inward EET for microbial electrosynthesis. (c) Scheme of bidirectional electron transfer in biohybrid systems for biosynthesis and biocurrent. The left side shows the applied potentials from the electrode, while the right side indicates representative redox potentials of EET processes in *S. oneidensis*. All potentials are versus Ag/AgCl. The potentials at −0.46 and −0.35 V correspond to flavin-mediated MET process, whereas −0.15 V represents cytochrome-dependent DET process. Red arrows indicate inward EET for microbial electrosynthesis, while blue arrows denote outward EET for electricity generation. (d) Scheme of biotic–abiotic biohybrid system for inward EET toward semibiological photosynthesis. Reproduced with permission from ref 161. Copyright 2023 Wiley-VCH.

*S. oneidensis* and *G. sulfurreducens* are known to possess the ability to perform bidirectional electron transfer, owing to their reversible electron conduits. A bioelectrochemical system was developed to demonstrate the bidirectional electron transfer capability of *S. oneidensis* by integrating conjugated polyelectrolytes with the cells (Figure 11c). At an applied positive potential of 0.2 V (vs Ag/AgCl), the biohybrid system produced bioelectricity (blue arrows), and when a negative potential of −0.6 V (vs Ag/AgCl) was applied, it was able to perform electrosynthesis of fumarate from succinate (red arrows).^161^ Differing from the outward EET pathway, the inward route relies heavily on outer membrane proteins, as the knockout of both MtrC and OmcA inhibited approximately 80% of electron uptake in *S. oneidensis*.^162^ When electrons are harvested from the outer membrane, a fraction of them will proceed in the reverse direction of the previously discussed Mtr pathway to the quinol pool for the production of NADH from NAD^+^. This consequently creates a proton motive force to synthesize ATP, therefore powering the reductive tricarboxylic acid (rTCA) cycle.

Building upon the intrinsic metabolic pathways of *S. oneidensis*, scientists have employed synthetic biology strategies to enhance its electron transfer capabilities. The overexpression of MtrC proteins in *S. oneidensis* cells has been found to boost the electroactivity of the species, resulting in an additional 35% increase in MFC current output compared to the wild-type strain.^163^ Moreover, various genetic modification techniques have been introduced to endow microbes with non-native capabilities such as carbon fixation. Earlier studies have presented genetically modified *S. oneidensis* that can perform nitrogen and carbon fixation. For instance, the microbial electrochemical system, featuring the *S. oneidensis*, has demonstrated effectiveness in converting nitrate to ammonia with an overall selectivity of 82.5% and a maximum ammonia yield of 24.3 μg h^–1^.^164^ In the work by Shi and co-workers, *S. oneidensis* genetically modified with reductive glycine (rGly) pathway was coupled with CdS nanoparticles to enable both solar-to-acetate conversion and autotropic cell growth, unlocking new opportunities for developing novel solar-activated biosynthetic applications.^165^ Meanwhile, research by Yang et al. constructed a bidirectional electron transfer system based on *G. sulfurreducens* biofilms and explored the underlying mechanisms by investigating the roles of individual redox proteins.^166^ This metabolic study of *G. sulfurreducens* has provided valuable insights for MFC applications, particularly due to its potential in electricity generation. More studies have examined their metabolic pathways, with a special focus on the types of substrates available and how they are transformed during the process. The work by Speers et al. has elucidated the energy metabolism of *G. sulfurreducens* by showing the sequential conversion steps of energy sources,^167^ such as lactate, acetate, and formate, to acetyl-CoA. Acetyl-CoA, as a metabolic intermediate, later enters the tricarboxylic acid (TCA) cycle to generate both energy and electrons. On this basis, Bond et al. highlighted the potential of *G. sulfurreducens* in MFC applications by emphasizing its ability to fully oxidize acetate and convert the chemical energy into a biocurrent of 65 mA m^–2^.^168^ Nevin and co-workers further employed a systems engineering approach to optimize the electrochemical cell design, thus achieving maximum current and power densities of 4.56 A m^–2^ and 1.88 W m^–2^, respectively.^169^

### 3.3 Integrating Microbe–Semiconductor Biohybrid Systems

Semibiological photosynthesis systems typically integrate nonphotosynthetic microbes with semiconductor materials to impart light-harvesting capabilities. To construct a light-driven biohybrid system, the primary design principle should focus on establishing a seamless interface between microbes and semiconductors to optimize electron transfer efficiency. This section, therefore, highlights the essential material selection criteria that ensure interfacial compatibility and facilitate smooth interactions between biotic and abiotic entities. A critical criterion is the energy level alignment of the redox potentials at the microbe–semiconductor interfaces (Figure 11d).^37,170,171^ In semibiological photosynthesis, the conduction band potential of the candidate semiconductor must be positioned at a more negative level than the thermodynamic potential of the microbial entities to drive the biologically relevant redox reactions.^37^

One of the representative examples is CdS, which has a bandgap of 2.4 eV and a conduction band potential of −1.0 V vs RHE. This bandgap corresponds to a maximum excitable wavelength of 514 nm, indicating that the photocatalyst can absorb light ranging from the violet to green region of the visible light spectrum. For example, in the CdS–*M. thermoacetica* biohybrid system,^118^ photoexcited electrons generated from CdS possess sufficient energy to activate the metabolic reactions of *M. thermoacetica*, including the regeneration of NAD^+^/NADH and NADP^+^/NADPH (standard redox potential at approximately −0.32 V vs NHE) and the conversion of CO_2_ to acetate (standard redox potential for HCO_3_^–^/acetate of −0.28 V vs NHE).^90^

A wide range of semiconductors have already been used to integrate different microbes to build semibiological biohybrid systems for STC conversion. Among these semiconductor photocatalysts, due to the simplicity of the synthesis method,^37^ hybrid systems that incorporate cadmium-based nanoparticles, especially CdS and CdSe, have been developed for various microbes, including CdS–*S. oneidensis*, CdS–*E. coli*, CdS–*G. sulfurreducens*, CdS–*S. ovata*, and CdS–*R. palustris*.^172^ Similarly, apart from inorganic semiconductors, organic compounds with adjustable molecular structure and good biocompatibility have also been utilized in biohybrid systems to promote interfacial interactions and facilitate electron injection. For instance, Yu et al. designed threonine deaminase-modified donor–acceptor conjugated polymer nanoparticles to couple with genetically engineered *E. coli*, and this biocompatible organic polymer–*E. coli* biohybrid system could enhance the threonine yield and further transform threonine to 2-oxobutyrate.^173^

In addition, other conventional redox reactions participating in biosynthetic processes include the reduction of microbial surface proteins (e.g., Omc, approximately at −0.15 V vs Ag/AgCl) and the redox cycling of other energy carriers.^37^ While for oxidative processes, the energy level alignment depends on the thermodynamic benchmark set by the valence band potential of photocatalysts or the electrode. Exemplary work by Edwards et al. has interfaced *S. oneidensis* with CdSe nanocrystalline photocatalysts to examine the feasibility of microbes donating electrons to the valence band holes of photocatalysts.^170^ CdSe, with a valence band potential of +1.0 V vs NHE,^170^ created a potential gap with OmcS of *S. oneidensis*, enabling electron migration to suppress electron–hole recombination and hence increasing system stability. In this review, we mainly focus on the recent achievements and progress of the inward EET process with a focus on interface design at the biohybrid interfaces for STC conversion.

### 3.4 Biocompatibility of Semiconductors with Microbes

One key challenge in designing high-performing microbial photosynthetic systems is balancing the material performance with biocompatibility. This requires the selection and development of semiconducting materials that not only provide efficient photoconversion but also maintain cell viability and essential metabolic functions.

Two primary sources of toxicity are the generation of reactive oxygen species (ROS) and the release of toxic metal ions, particularly from inorganic semiconductors. ROS, such as hydroxyl radicals (•OH), hydrogen peroxide (H_2_O_2_), and superoxide (^•^O_2_^–^) are highly reactive molecules that are often generated at the surface of photocatalysts in aerobic biological processes.^174^ The standard redox potentials for ROS (O_2_/^•^O_2_^–^, O_2_/H_2_O_2_, and OH^–^/^•^OH at approximately −0.33 V,^175^ 0.28 V,^176^ and 1.8 V^177^ vs NHE, respectively) have established the thermodynamic barriers for oxidative processes. Once the concentration of ROS exceeds the minimum threshold, it induces oxidative stress, disrupting cellular metabolic activities, and significantly compromising system performance. This makes semiconductors with minimal ROS generation or ROS-scavenging capabilities particularly attractive.

The second challenge concerns metal ion leaching due to photocorrosion and redox cycling of inorganic materials. While microorganisms have evolved to tolerate toxic metal ions, such as Hg^2+^, Zn^2+^, Pb^2+^, and Cu^+/2+^, the presence of high concentrations remains toxic and compromises long-term stability. It is noteworthy that certain microorganisms are capable of detoxifying heavy metal ions by transforming them into less harmful compounds.^178^ One representative example is *M. thermoactica* which has been shown to biomineralize Cd^2+^ into CdS nanoparticles when a suitable amount of sulfur is supplied.^118^ Besides, using biocompatible organic semiconductors in semibiological photosynthesis to avoid the use of metal ions is also a promising strategy. For example, Li et al. integrated a zirconium-based porphyrinic metal–organic framework of PCN-222 with engineered *E. coli* for solar-driven H_2_ and lysine production.^179^

## 4 Design of Microbe–Semiconductor Biohybrid Interfaces

The integration of microbes and semiconductors to build biohybrids has gained growing interest in recent years for converting solar energy into chemical energy.^180^ In the microbe–semiconductor biohybrid systems, establishing a robust and functional biohybrid interface that achieves effective charge transfer between microbes and semiconductors remains a challenge.^174,181^ The quality of the biohybrid interfaces critically affects the interfacial electron transfer and the overall performance of the biohybrid system.^182,183^ In this section, a detailed understanding of assembly of microbe–semiconductor interfaces and their interactions is summarized.

### 4.1 Assembly of Microbe–Semiconductor Biohybrids

Current assembly methods of microbe–semiconductor biohybrids can be divided into two different categories: in situ and ex situ assembly (Figure 12). The in situ assembly of biohybrids means the nanoparticles are bioprecipitated by the bacteria themselves,^184^ while the ex situ assembly of biohybrids mentions that the synthetic nanoparticles are directly added into the culture media of bacteria.^185^ These two methods involve different mechanisms of biohybrid formation and exhibit distinct characteristics and impacts on STC conversion.

**Figure 12:**
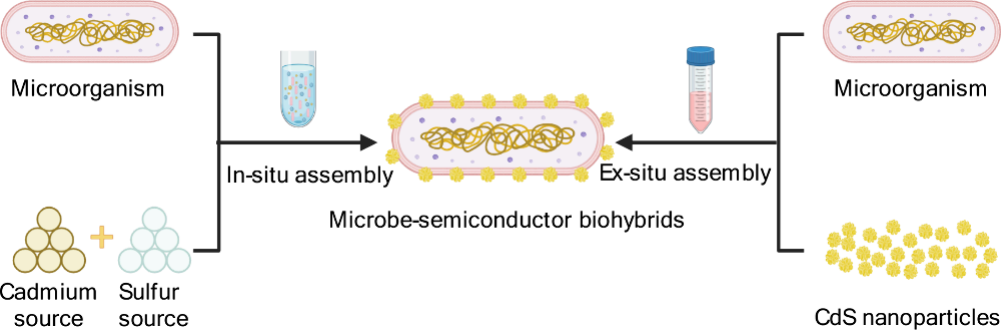
In situ assembly and ex situ assembly of microbe–semiconductor biohybrids. Cadmium source, Cd^2+^; sulfur source, inorganic S^2–^ and sulfur-containing biomolecules like cysteine.

#### 4.1.1 In Situ Assembly of Microbe–Semiconductor Biohybrids

In the in situ assembly of microbe–semiconductor biohybrids, nanoparticles are generated either around the bacterial cells or within their intracellular matrix through metabolic processes upon the introduction of precursor materials. One of the most typical examples is the bioprecipitation of CdS nanoparticles on the bacterial surface by culturing the cells in a solution containing Cd­(NO_3_)_2_and cysteine as the sulfur source. For example, as shown in Figure 13a, Han and co-workers assembled CdS nanoparticles on the surface of *S. oneidensis* to understand biotic–abiotic interfacial behavior.^186^ Under light illumination, the photoexcited electrons from CdS nanoparticles could transfer to *S. oneidensis*, achieving the significant upregulation of the H_2_ yield, ATP, and NADH/NAD^+^. A similar CdS–*S. oneidensis* biohybrid system was also built using this in situ assembly method (Figure 13b).^187^ By knocking out mutants of different proteins involved in EET pathways, we have investigated the electron transfer pathway in the CdS–*S. oneidensis* biohybrid system for H_2_ production. In another example, The anaerobic bacterium CdS–*M. thermoacetica* biohybrids enabled light-driven production of acetic acid from CO_2_ with a selectivity of 90% over several days of light–dark cycles. (Figure 13c).^118^ The use of bioprecipitated nanoparticles to fabricate photosynthetic biohybrids have indeed been extensively studied.^186–188^ Besides, some bacteria like *S. oneidensis* have been shown to biomineralize other metals like Fe^3+^, Mn^2+^ and Pd^2+^.^189–192^

**Figure 13:**
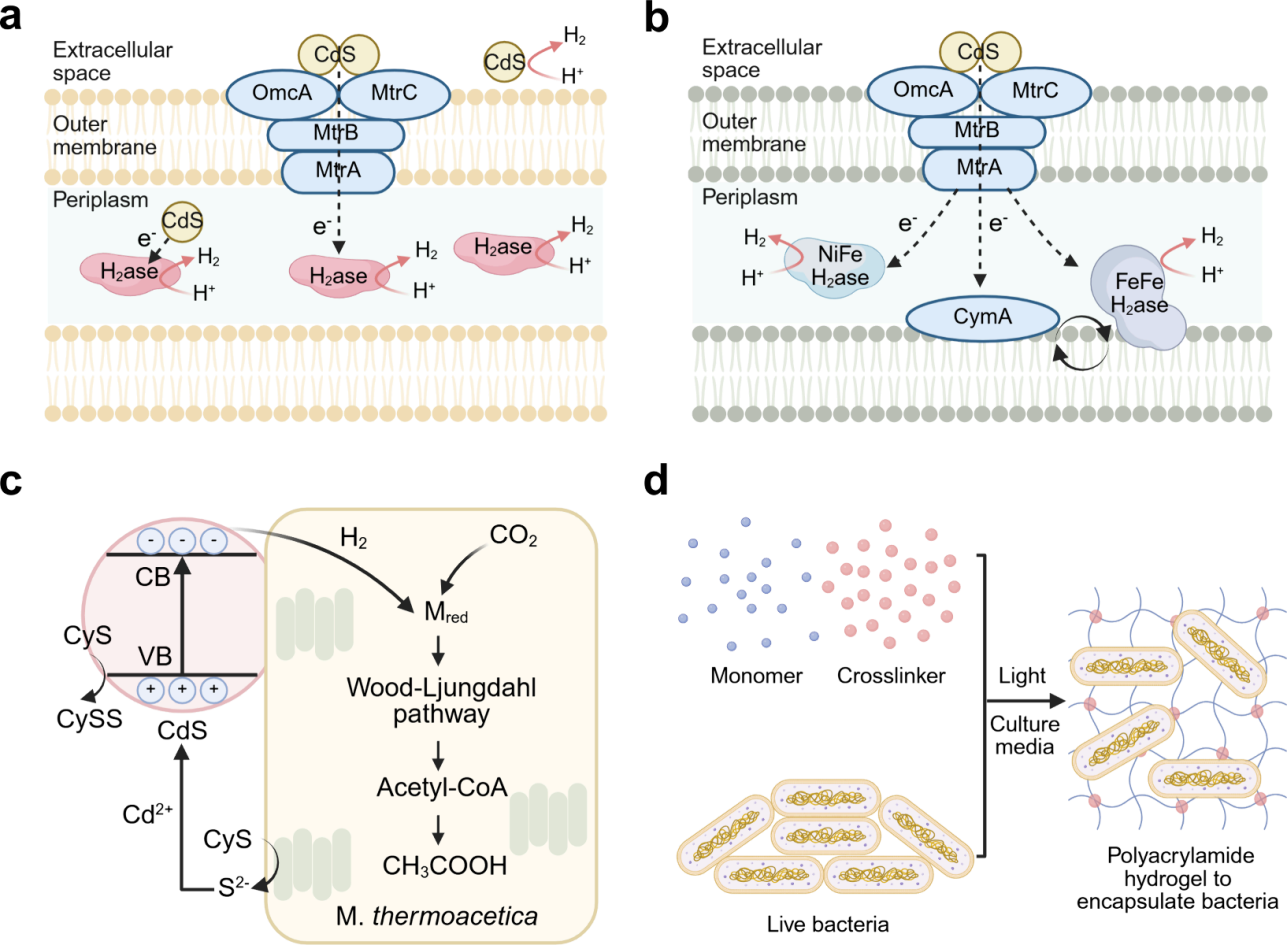
In situ bioprecipitation method to construct microbe–semiconductor biohybrids. (a) The light-driven H_2_ production by the in situ assembled CdS–*S. oneidensis* photosynthetic system. (b) The electron transfer pathway of H_2_ production in the bioprecipitated CdS–*S. oneidensis* biohybrid. (c) Self-photosensitization of CdS–*M. thermoacetica* biohybrid system for acetic acid production. (d) Bacteria-mediated photopolymerization of acrylamides. Reproduced with permission from ref 186. Copyright 2022 American Chemical Society. Reproduced with permission from ref 187. Copyright 2023 Elsevier. Reproduced with permission from ref 118. Copyright 2016 American Association for the Advancement of Science. Reproduced with permission from ref 195. Copyright 2021 Chinese Chemical Society.

Some reports have also reported that grafting functional polymers on the surfaces of live cells to enhance the performance of biohybrids using in situ polymerization methods.^193^ Niu et al. designed the cell surface-initiated controlled radical polymerization strategy to grow polymers on the surfaces of live yeast and mammalian cells, while maintaining high cell viability.^194^ The photoinduced polymerization method to prepare acrylamides via bacteria-initiated radical polymerization has also been discovered in Luria–Bertani medium under visible-light irradiation (Figure 13d).^195^ Mechanistic investigation reveals that this photoinduced polymerization is initiated by the hydroxyl radicals from converting dissolved oxygen. These in situ biosynthetic nanoparticles ensure good contact and proximity with cells, facilitating electron transfer at the biohybrid interfaces.^196^ Additionally, the in situ constructed biotic–abiotic biohybrid systems with self-regulated architectures can easily adapt to outer environments changes.^197^ However, it should be emphasized that achieving precise control over the size, morphology, and crystallinity of semiconductor nanoparticles regulated by microbes remains challenging.

#### 4.1.2 Ex Situ Assembly of Microbe–Semiconductor Biohybrids

When it comes to ex situ assembly of biohybrid systems, the semiconductor nanoparticles are first synthesized and subsequently introduced into microbial systems.^198^ In this situation, the synthetic semiconductors can be finely regulated to achieve desirable size and morphology depending on the requirements of the intended biohybrid systems.^199^ Physical adsorption, electrostatic interactions, and covalent conjugation occur in the ex situ assembly of artificial semiconductor–microbe hybrids.^37,197^ Physical adsorption usually relies on intermolecular forces and hydrogen bonds to integrate semiconductor nanoparticles and microbes.^37^ Due to its easy availability and good compatibility with many semiconductors and microbes, this approach has been widely used in the construction of biohybrid systems, such as CdTe quantum dots (QDs)–*Xanthobacter biohybrids*,^200^ silicon nanowire–*S. ovata* biohybrids.^127^ He and colleagues build a CdS–*S. ovata* biohybrid system for photocatalytic CO_2_ fixation to acetic acid via directly mixing CdS and *S. ovata* (Figure 14a).^201^ Notably, such assembly often results in weak interactions and poor contact at the microbe–semiconductor interfaces. Electrostatic interactions, arising from the attraction between oppositely charged nanoparticles and microorganisms, can facilitate a more intimate and stable connection.^202^ For example, the positively charged perylene diimide derivative (PDI) and poly­(fluorene-*co*-phenylene) (PFP) interacted intimately with negatively charged *M. thermoacetica* (Figure 14b), enabling acetate production from CO_2_ with a high efficiency of 1.6%.^203^ Moreover, these cationic side chains could intercalate into the cell membrane by hydrophobic interactions, thus facilitating efficient electron transfer to bacteria. As shown in Figure 14c, Zhou et al. developed a conjugated polymer (PyTT-tBAL-HAB) to interact electrostatically with *E. coli* for enhanced solar-driven H_2_ production.^204^ The PyTT-tBAL-HAB has a narrow bandgap (1.16 eV) and exhibits unique IR light absorption properties, ultimately achieving a quantum efficiency of 18.4% at 940 nm.

**Figure 14:**
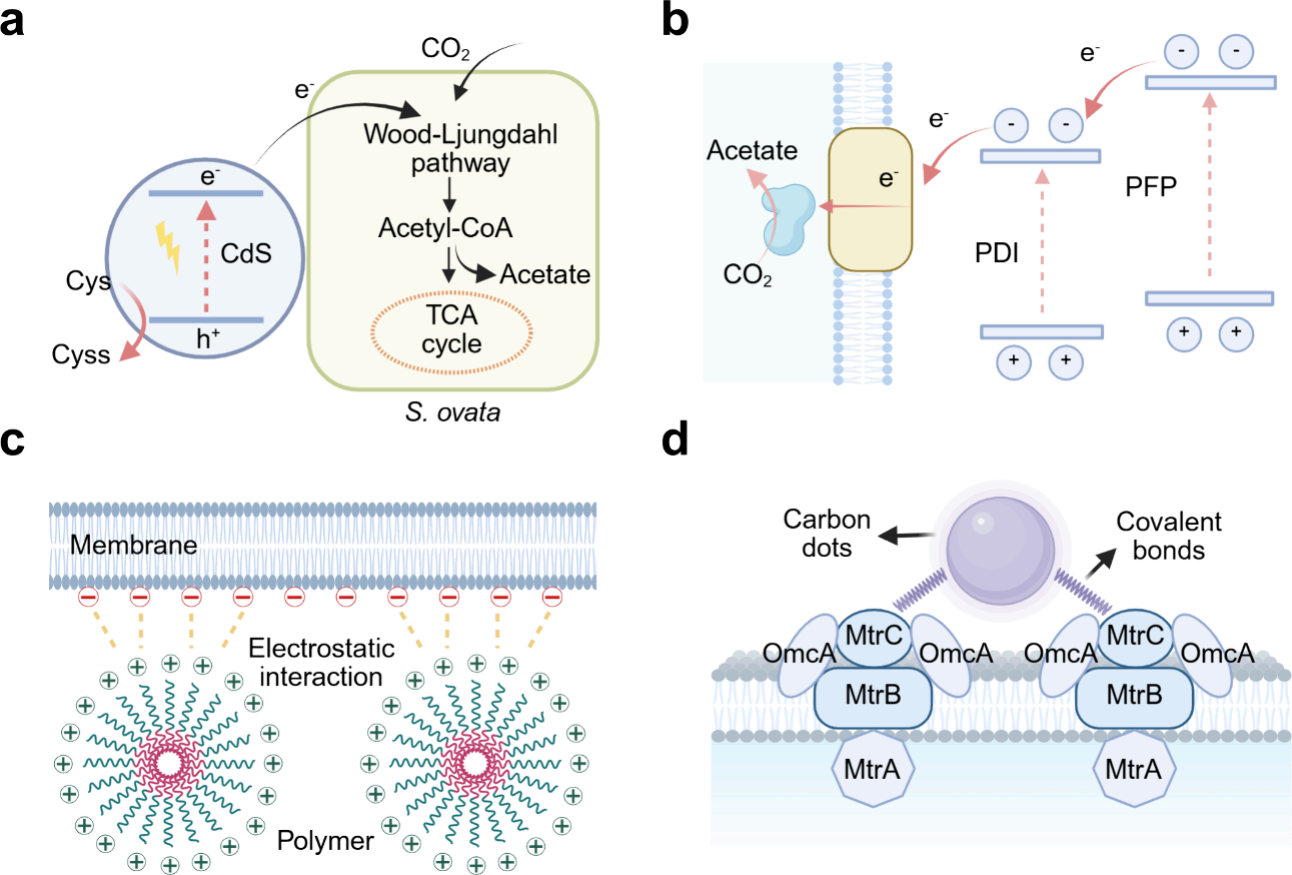
Ex situ assembly of biohybrid systems to construct biotic–abiotic biohybrids. (a) Schematic illustration of CdS–*S. ovata* biohybrids via physical adsorption. (b) The electrostatic assembly of PDI/PFP–*M. thermoacetica* biohybrid via for CO_2_-to-acetate production. (c) The electrostatic interactions between conjugated polymer (PyTT-tBAL-HAB) and *E. coli*. (d) Covalently grafting CDs onto a *S. oneidensis* membrane via the copper-catalyzed azide–alkyne click reaction. Reproduced with permission from ref 201. Copyright 2022 American Chemical Society. Reproduced with permission from ref 203. Copyright 2020 Wiley-VCH. Reproduced with permission from ref 204. Copyright 2025 Wiley-VCH. Reproduced with permission from ref 206. Copyright 2025 Elsevier.

Even though these noncovalent connections have demonstrated great success in the assembly of microbe–semiconductor biohybrids, integrating microbes and semiconductors via covalent coupling to form stable chemical bonds are more favorable for biohybrid interfacial electron transfer.^197^ These chemical reactions rely on cross-linking between functional groups on the microbial surface and semiconductor surfaces,^205^ representing the unique advantage of high specificity to connect microbes and semiconductors. For example, Zhu and colleagues reported a judicious work of covalently grafting carbon dots (CDs) on the surface of bacterial cell membrane via the copper-catalyzed azide–alkyne click reaction (Figure 14d).^206^ Under light irradiation, this covalent interaction could enable effective electron transfer at the CDs–*S. oneidensis* interface, and this biohybrids exhibit a 7.3-fold enhancement of H_2_ production. This work highlights a promising strategy of employing covalent connections to construct efficient microbe–semiconductor biohybrid photosynthetic systems. Meanwhile, we have designed a photosensitized-MtrC biohybrid by coupling graphitic nitrogen doped carbon dots (g-N-CDs) with the outer-membrane decaheme protein, MtrC from *S. oneidensis*.^207^ The g-N-CDs were surface-functionalized with a maleimide derivative via either carbodiimide chemistry or acyl chloride activation, followed by coupling with a cysteine residue of Y657CMtrC mutant at the surface of *S. oneidensis*. Photophysical characterizations showed that ultrafast electron transfer showed up from g-N-CDs to MtrC proteins, suggesting a promising strategy to drive photoinduced transmembrane charge transfer in *S. oneidensis*.

Together, building intimate and effective interfaces between semiconductors and microbes or proteins is of great importance for achieving efficient STC conversion.^208^ The assembly and interactions at the microbe–semiconductor interfaces are influenced by many different factors.^209^ The detailed understanding of interfacial charge transfer dynamics needs a mechanistic and time-resolved approach,^210^ which deserves further investigation to advance and accelerate the development of biotic–abiotic biohybrid systems.^211^

### 4.2 Materials Design

Engineering biohybrid interfaces can be divided into two directions: materials design and biological design.^106^ Materials design refers to the modification of the semiconductor surface and structure for enhanced interfacial electron transfer. In this section, we will highlight some key strategies to improve the performance of biotic–abiotic hybrid systems, including adding redox mediators, engineering conductive layers, and structural modification.^212,213^

#### 4.2.1 Adding Redox Mediators

In microbe–semiconductor hybrid systems, engineering a low-resistance interface is essential for achieving efficient STC conversion. Adding redox mediators is a straightforward and feasible way to enhance the electronic interactions between semiconductors and microbial cells. Some soluble electron shuttles such as methyl viologen (MV)^214^ and anthraquinone-2,6-disulfonate (AQDS)^215^ have been widely used to enhance electron transport from semiconductors to microorganisms. As shown in Figure 15a, biocompatible organic semiconductor polymer dots (Pdots) were integrated with *R. eutropha* H16 (RH16) to achieve efficient CO_2_ utilization by adding NR. The NR, functioning as the electron shuttle, could effectively transfer photogenerated electrons from Pdots into RH16, facilitating the Calvin cycle to convert CO_2_ into poly-3-hydroxybutyrate (PHB), which exhibited 3-fold enhancement than that of original RH16.^11^ Martins and co-workers investigated the photocatalytic H_2_ production by integrating self-precipitated CdS nanoparticles with three different microbial cells, *Desulfovibrio desulfuricans* (*D. desulfuricans*), *Citrobacter freundii*, and *S. oneidensis*.^216^ With the addition of MV to the CdS–*D. desulfuricans* biohybrid system, a higher H_2_ production rate was observed as compared to the system without MV. Similarly, Honda et al. designed a hybrid system consisting of TiO_2_, MV, and *E. coli* (Figure 15b).^217^ The MV again functioned as an electron mediator to transfer photogenerated electrons from TiO_2_ to *E. coli*, thereby enhancing biohydrogen production.

**Figure 15:**
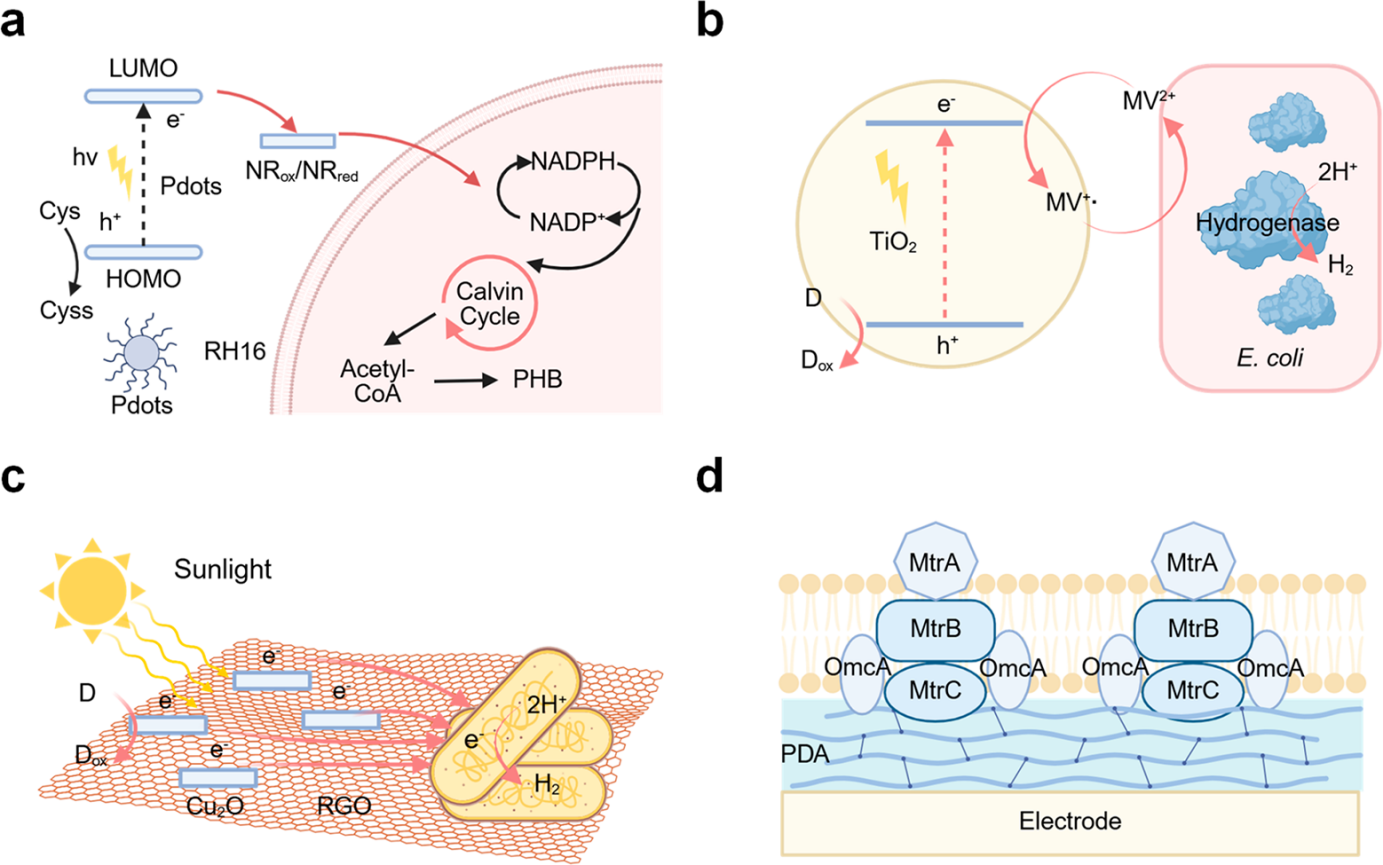
Adding redox mediators and engineering conductive layers at the biohybrid interfaces. (a) Pdots–RH16 biohybrid system for the synthesis of PHB. (b) TiO_2_/MV/recombinant *E. coli* biohybrid for H_2_ production. (c) Loading RGO as a platform in the Cu_2_O/RGO–*S. oneidensis* biohybrid system. (d) Engineering single-cell collector in the PDA–*S. oneidensis* biohybrid system. Reproduced with permission from from ref 11. Copyright 2023 American Chemical Society. Reproduced with permission from ref 217. Copyright 2016 Wiley-VCH. Reproduced with permission from ref 223. Copyright 2020 American Chemical Society. Reproduced with permission from ref 227. Copyright 2020 Springer Nature.

These examples demonstrate that adding redox mediators to biohybrid systems is a viable strategy for the design of interfacial electron transfer for sustainable energy conversion.^217^ Yet, the use of redox mediators can lead to some side reactions like electron transfer to alternative acceptors in the medium, ultimately decreasing the overall electron utilization efficiency.^218^ Besides, some redox mediators are toxic to microorganisms, posing a major challenge for long-term sustainable biohybrid systems.^197^ The additional cost of redox mediators is also worth considering in practical implementation.

#### 4.2.2 Engineering Conductive Layers

To overcome the limitations of soluble redox mediators, building solid conductive layers as intermediaries at the microbe–semiconductor interfaces has received increasing attention.^219^ The solid conductive interfaces serve to bridge the materials and biological components, enabling efficient electron transfer across the biohybrid interfaces.^220^ Different from diffusive redox mediators, the solid conductive layers provide superior electron transfer efficiency and stable interfacial combinations.^221^

One of the typical conductive layers is solid two-dimensional (2D) materials, such as graphene and black phosphorus.^222^ These 2D materials can be decorated as films, nanostructured coatings, and scaffolds in the biotic–abiotic hybrid system to offer highly conductive interfaces. For example, reduced graphene oxide (RGO), a classical 2D material, has extraordinary electronic and optoelectronic properties and has been applied for catalysis and optoelectronics.^223^ Loading RGO at the Cu_2_O–*S. oneidensis* interfaces could provide an electron-shuttling pathway and guarantee efficient electron transport from Cu_2_O to *S. oneidensis* under irradiation (Figure 15c). As a result, the Cu_2_O/RGO–*S. oneidensis* biohybrids showed an obvious enhancement of H_2_ production compared to that of Cu_2_O/RGO–*S. oneidensis* and Cu_2_O/organic electron mediator–*S. oneidensis*.

Another common strategy is to coat conductive polymers around the microbial cells to increase interfacial contact for efficient charge transfer.^224,225^ Conductive polymers like polydopamine (PDA) and polypyrrole (PPy) can be electropolymerized or photopolymerized on semiconductors or microbe surfaces.^219,224,226^ Recent work has shown the design of a single-cell collector, constructed by conductive polymers like PDA, to encapsulate *S. oneidensis* cells (Figure 15d).^227^ The in situ synthesized single-cell electron collector could generate an intimate conductive layer at the interfaces and maximize the contact area, thereby realizing a record-high interfacial electron transfer efficiency. A similar strategy is also applicable in the PDA-*Rhodobacter capsulatus* (*R. capsulatus*) biohybrid systems for H_2_ and NH_3_ synthesis.^228^ Liu et al. also coated PDA on individual *Shewanella xiamenensis* (*S. xiamenensis*) cells to facilitate EET process.^229^ In addition, inorganic nanoparticles can also act as an interfacial conductive layer. By biosynthesizing FeS nanoparticles at the interfaces, the electron uptake and metabolic rate in *Desulfovibrio vulgaris* were about 7-fold higher than those in native cells.^230^ Through incorporating NiCu alloys at the CdS–*M. barkeri* interfaces, the biohybrid systems could achieve high product selectivity for CH_4_ synthesis.^220^

Besides, constructing conductive layers at the biohybrid interfaces can also reduce the mismatches between electron generation rates and electron utilization rates.^231^ For example, the different electron generation rate of CdS (3 × 10^–11^ s) and electron utilization rate of methanogen metabolism (10^–4^ to 10^1^ s) usually lead to low CH_4_ selectivity and quantum yield.^232,233^ Hu et al. synthesized metal-free polymeric carbon nitride (CN_*x*_) decorated with cyanamide (NCN) groups and then integrated the methanogen *M. barkeri* for solar-driven CO_2_-to-CH_4_ conversion.^232^ The NCN with unique capacitance and conductive properties could govern the biotic–abiotic interfaces for electron storage and redistribution. The ^NCN^CN_*x*_–*M. barkeri* biohybrid system showed a high quantum yield of 50.3%, with a remarkable selectivity of 92.3% for CH_4_ production. This interfacial design offers a promising opportunity to build microbe–semiconductor biohybrids for microbial photosynthesis with high quantum yield and selectivity.

#### 4.2.3 Structural Modification

^10^ Optimizing the semiconductor structure and surface states not only helps maintain physical compatibility with microbe cells but also facilitates rapid and directional electron exchange at the microbe–semiconductor interfaces.

Incorporating different functional groups at the material surface is a practical way to improve cell-material interactions.^234,235^ For example, Au nanoparticles with different surface functionalities have been investigated to understand their different interactions with photosynthetic bacteria *R. capsulatus* (Figure 16a).^236^ The Au nanoparticles were decorated with four different functionalized groups: the reducing agent tetrakis­(hydroxymethyl)­phosphonium chloride (THPC), THPC combined with cysteine, mercaptopropionic acid, and cysteamine. It was found that the Au nanoparticles with cysteine in the zwitterionic form allowed the uniform distribution of Au nanoparticles within the outer membrane/periplasmic space of *R. capsulatus*, which exhibited enhanced EET and (photo)­current generation. Modifying semiconductors with the residues of proteins and amino acids is also a useful and feasible approach to optimizing the surface chemistry.^237^ As depicted in Figure 16b, integrating the membrane-anchoring protein incorporated with a TiO_2_ binding peptide on the engineered *E. coli* exhibited an 81-fold enhancement in electron transfer efficiency and a 10.8-fold increase in H_2_ evolution.^238^ Combining glutathione-modified CuInS_2_/ZnS QDs into *Azotobacter vinelandii* (*A. vinelandii*) could also promote the effective solar-driven N_2_ fixation.^239^ The strategy of surface modification enlightens the rational design of conductive mediators for the future development of biohybrid systems.

**Figure 16:**
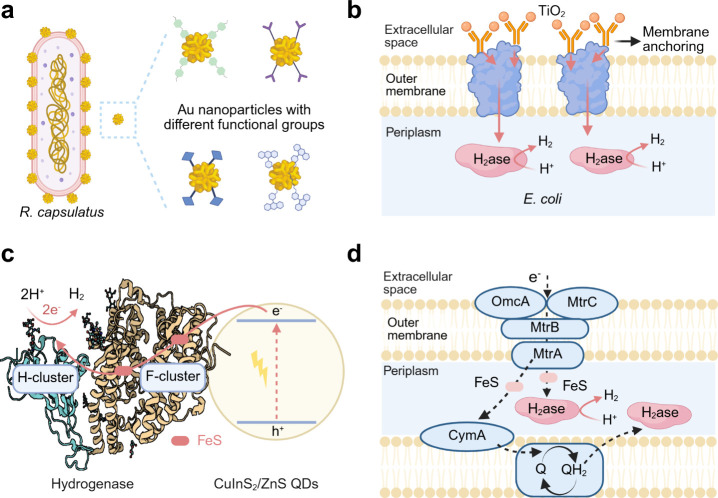
Structural modification at the biohybrid interfaces. (a) Understanding the Au nanoparticles–*R. capsulatus* interactions with four different functional groups. (b) The membrane-anchoring protein incorporated with a TiO_2_ binding peptide on the engineered *E. coli* for enhanced H_2_ production. (c) Synthesizing 7 nm CuInS_2_/ZnS QDs into the periplasm of *S. oneidensis* for H_2_ production. H-cluster, active center; F-cluster, Fe–S cluster. (d) Engineering FeS nanoparticles into the periplasm of *S. oneidensis* to enhance electron transfer. Reproduced with permission from ref 236. Copyright 2024 American Chemical Society. Reproduced with permission from ref 238. Copyright 2024 American Chemical Society. Reproduced with permission from ref 198. Copyright 2021 Wiley-VCH. Reproduced with permission from ref 243. Copyright 2024 National Academy of Sciences.

Optimization of the semiconductor’s morphology, size distribution, and architecture also has a significant influence on interfacial electron transfer.^212^ Morphologies and structures like nanowires and core–shell structures enable increased surface area and more available contact with microbial cells.^127,211^ Choi et al. developed a hierarchical 3D percolation network of Ag–Au core–shell nanowire–hydrogel composite using a simultaneous nanowelding synthesis method.^240^ Applying this unique material as a metal electrode in the microbial fuel cells eventually showed superior electron transfer and ultrahigh power density (∼330 W m^–3^).

In addition, by regulating the surface functional groups and size distributions of semiconductors in the biotic–abiotic hybrid systems,^241^ semiconductors could also enter the periplasm or cytoplasm, functioning as an intracellular photosensitizer to reduce charge transfer distance.^37^ Zhang and colleagues synthesized gold nanoclusters as the intracellular photosensitizer in the cytoplasm of *M. thermoacetica* to directly transport electrons to enzymes for CO_2_-to-CH_3_COOH reduction.^242^ Luo and co-workers successfully delivered the 7 nm CuInS_2_/ZnS QDs into the periplasm of *S. oneidensis* for enhanced H_2_ production (Figure 16c).^198^ Tu et al. also fabricated FeS nanoparticles in the periplasm to establish a direct electron transfer chain from RGO electrode to the periplasm hydrogenases for solar-driven H_2_ production (Figure 16d).^243^ In short, constructing intracellular electron generators can overcome the slow transmembrane processes and facilitate direct electron transfer to enzymes. This judicious design, with shorter electron tunneling distance and higher electron collection efficiency, sheds light on a promising approach to enhancing electron transfer dynamics in the biotic–abiotic hybrid systems.

However, although these studies demonstrate that intracellular semiconductors exhibit good biocompatibility, their presence may increase the risk of cytotoxic effects, including membrane disruption and interference with intrinsic metabolic processes.^244,245^ Besides, sacrificial agents such as cysteine or ascorbic acid are commonly added in these systems; nevertheless, endogenous reducing equivalents such as NADH/NADPH and reduced metabolites can also function as electron donors.^246^ This coexistence complicates the identification of intracellular electron transfer pathways and obscures a clear understanding of intracellular redox processes. Therefore, whether the intracellular semiconductors are favorable requires further investigation.^247^

### 4.3 Biological Design

Complementary to material modifications, biological engineering can promote the interface interactions and enhance interfacial electron transfer through genetic and synthetic approaches.^172,212,248^ Current biological engineering is mainly focused on optimizing electron transfer pathways and intracellular metabolism.

#### 4.3.1 Optimizing Electron Transfer Pathways

The sluggish electron transfer at the biotic–abiotic interfaces cannot provide sufficient electrons or reducing equivalents for intracellular metabolism, resulting in the unsatisfactory performance of chemicals synthesis.^38^ Using genetic engineering to enhance mediated and direct EET pathways at the microbe–semiconductor interfaces is a promising strategy. The overexpression of redox mediators like flavins can obviously promote indirect electron transfer at the biohybrid interfaces. Taking *S. oneidensis* as a demonstration, the low concentration of secreted flavin in the wild-type *S. oneidensis* lead to its low EET efficiency in semiartificial photosynthesis. By transferring the flavin biosynthesis pathway from *Bacillus subtilis* (*B. subtilis*) to *S. oneidensis*, the engineered *S. oneidensis* successfully expressed a 25.7-fold enhancement in secreted flavin concentration.^94^ Using this recombinant *S. oneidensis* as the model bacterium for the reaction of fumarate-to-succinate, the inward current density was ∼15.5 times higher than that of wild-type *S. oneidensis*. A similar strategy of engineering efficient riboflavin secretion pathway from *B. subtilis* and *Pseudomonas aeruginosa* (*P. aeruginosa*) to *S. oneidensis* was also carried out to facilitate flavin-mediated transmembrane electron injection (Figure 17a).^249^ Specifically, the riboflavin synthesis genes of ribA, ribD, ribE, ribH, and ribC from *B. subtilis* were translated into *S. oneidensis* to obtain the recombinant strain MC. Then, the pore protein OprF from *P. aeruginosa* and the endogenous transporter Bfe were employed to express in the recombinant strain MC to generate the recombinant strain MCO. The secreted flavins by the recombinant MC and MCO exhibited 5.8- and 7.3-fold higher than that of wild *S. oneidensis*. Also, Lin et al. used synthetic biology approaches to promote flavin biosynthesis in *S. oneidensis* and further facilitate EET processes, finally achieving a high inward current density of 18.78 A m^–2^.^250^

**Figure 17:**
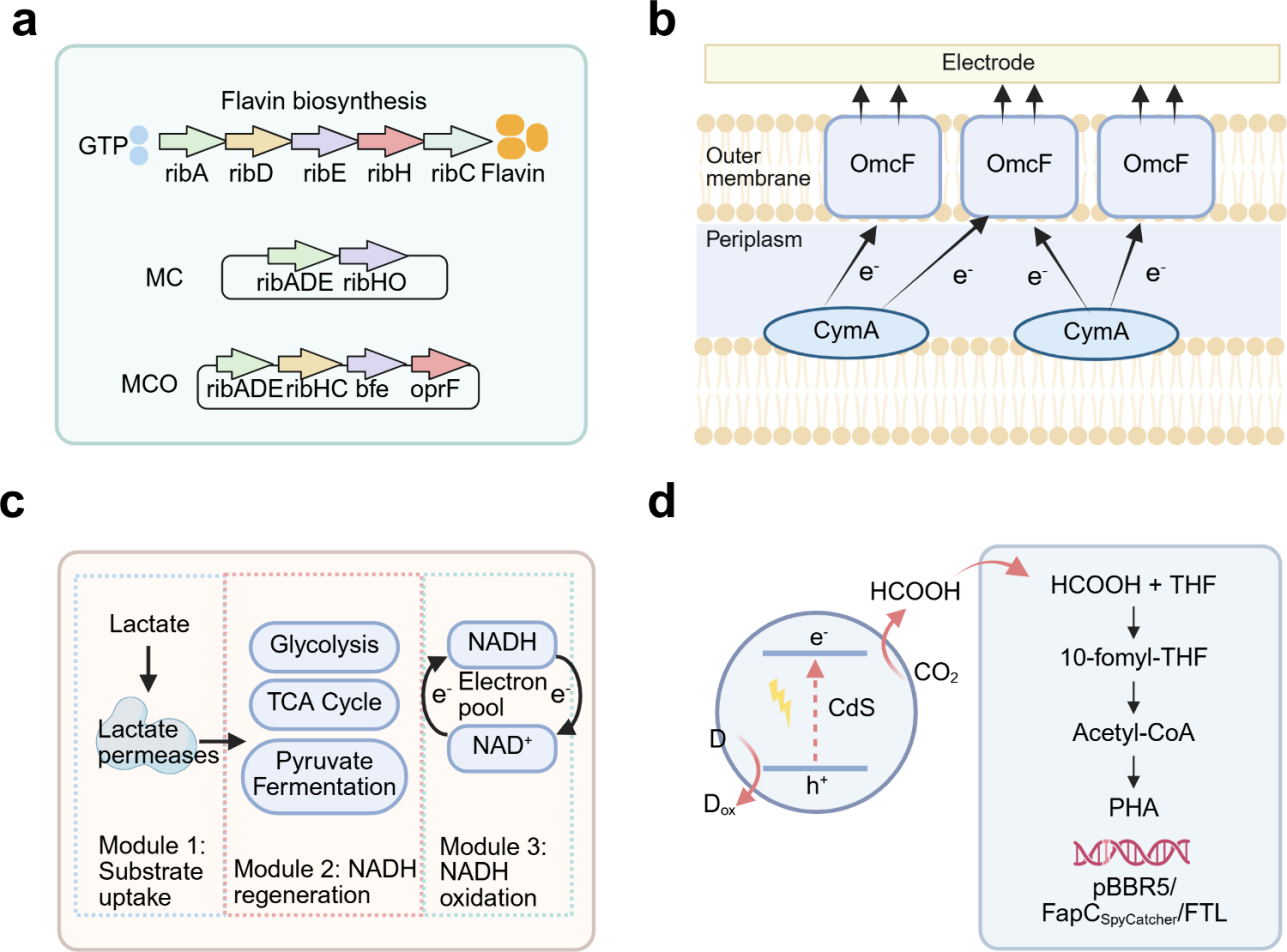
Biological designs at the biohybrid interfaces. (a) Constructing a flavin biosynthesis pathway to accelerate flavin-mediated indirect electron transfer. (b) Reconstructing OM c-Cyts OmcF conductive channel to accelerate transmembrane electron transfer. (c) Modular engineering to broaden the sources of the intracellular electron pool, enhance intracellular NADH regeneration, and promote electron release from electron pools in engineered *S. oneidensis*. (d) Living photosensitizer-mineralized biofilms for photocatalytic CO_2_ reduction into PHA. Reproduced with permission from ref 249. C opyright 2024 Wiley-VCH. Reproduced with permission from ref 256. Copyright 2023 American Chemical Society. Reproduced with permission from ref 261. Copyright 2025 Elsevier.

Despite enhanced electron transfer from the overexpressed of flavin secretion, the inefficient transmembrane transport and slow electron transfer would also limit the widespread application.^251^ Thus, designing a cytochrome C-based DET pathway seems more attractive. To improve DET in *S. oneidensis* cells, Liu et al. utilized protein engineering to screen and optimize the OM c-Cyts OmcF from *G. sulfurreducens* (Figure 17b).^249^ The engineered *S. oneidensis* with the reconstructed OM c-Cyts conductive channel could accelerate cytochrome-based transmembrane electron transport and reduce the interfacial electron transfer resistance. Similarly, the DET conduit can also be constructed into electrically inactive bacteria to endow them the ability of EET. Wu and co-workers engineered the MtrABC pathway and the inner membrane associated quinol oxidase CymA of *S. oneidensis* into *E. coli* for biological electron transfer, ultimately promoting electron utilization for CO_2_ fixation and synthesis of reductive products.^252^

With the assistance of genetic tools and synthetic biology, the DET and MET pathways can overcome slow electron transfer rate at the abiotic-biotic interface,^212^ thereby enhancing electron transfer rates and facilitating more efficient production of value-added chemicals in biohybrid systems.^253^

#### 4.3.2 Engineering Intracellular Metabolism

Apart from the engineered electron transfer pathways such as promoting redox mediator secretion and editing MtrABC pathway, another very promising way of biological engineering is to regulate the intracellular metabolic activities of these bioactive bacteria.^172^ Through redirecting metabolic fluxes, redesigning native metabolic pathways, and inserting unique synthetic modules, microbial cells can possess new possibilities to produce higher reducing power and transfer more electrons to subsequent metabolic activities.^254^ Herein, redesigning intracellular metabolism mainly discusses intracellular metabolism like overexpressing reducing equivalents NADH/NAD^+^.^255^

The injected electrons are usually stored in the form of NADH, a major intracellular electron carrier.^186^ Endeavors toward synthetic biology strategies like promoting NADH production and regulating the NADH/NAD^+^ ratio have been made to optimize the accessible intracellular electron pool for EET processes. For instance, Ding and colleagues utilized a modular engineering strategy to broaden the sources of the intracellular electron pool (module 1), enhance intracellular NADH regeneration (module 2), and promote electron release from electron pools (module 3), thereby redirecting electron flux into the electron transfer chain in *S. oneidensis* (Figure 17c).^256^ Through editing four different genes (*SO1522* encoding a lactate transporter, *gapA* encoding a glyceraldehyde-3-phosphate dehydrogenase, *mdh* encoding a malate dehydrogenase, and *ndh* encoding NADH dehydrogenase) in *S. oneidensis*, intracellular NADH levels were increased by 62%, which was beneficial for intracellular electron flow. Apart from modulating the intracellular NADH/NAD^+^ ratio, enhancing the total intracellular NAD­(H/^+^) level is also an effective way to improve the EET rate.^257^ By engineering three modules of de novo biosynthesis (module 1), salvage biosynthesis (module 2), and universal biosynthesis (module 3) in *S. oneidensis*, Li et al. successfully redirected electron flux toward the biosynthesis of intracellular NAD­(H/^+^) level. Results showed that the total NAD­(H/^+^) level of engineered *S. oneidensis* achieved a 2.1-fold enhancement than that of wild *S. oneidensis*, thereby inducing more electron transport and enhancing the EET rate.^255^

Additionally, in microbe–semiconductor biohybrid systems, biofilms grown on semiconductors or electrodes can facilitate electron uptake into microbial cells. Thus, rational design of biofilms also offers a new option to enhance interface interactions and catalytic activity.^258–260^ As shown in Figure 17d, Wang and co-workers engineered the SpyCatcher domain into the amyloid protein FapC to construct functional FapCSpyCatcher nanofibrils in *Pseudomonas fluorescens* (*P. fluorescens*), which could promote the self-assembly of extracellular amyloid biofilms and functionalize the biofilms with desired proteins.^261^ Further in situ mineralization of CdS nanoparticles on *P. fluorescens* biofilm nanofibrils, the CdS–*P. fluorescens* biohybrid not only retained efficient photocatalytic performance to produce polyhydroxyalkanoates (PHAs) but also protected cells from irradiation damage. Meanwhile, Li et al. engineered transmembrane pili conduit and incorporated CdS nanoparticles to promote electron transfer across biohybrid interfaces and biofilm matrices.^262^ The generated biofilm could maintain cellular viability, preserve hydrogenase activity, and maintain long-term stability for plastic-to-hydrogen conversion for 63 days. Moreover, the genetic biofilm with transmembrane pili-based nanowire could further enhance electron transfer for enhanced H_2_ production from 3751 μmol g^–1^ to 5862 μmol g^–1^.

Another work of increasing biofilm formation has also been reported via a modular engineering, which includes improving the component biosynthesis of extracellular polymer substrates (EPS) matrix, regulating intracellular c-di-GMP level, and constructing artificial hybrid system.^263^ First, expression of *mxdB* encoding a glycosyltransferase from *S. oneidensis* and *pilA* encoding a type IV pili assembly protein from *P. aeruginosa* facilitated the synthesis of EPS matrix. Then, three diguanylate cyclase genes from *E. coli* (*ydaM*, *ydeH*, and *yedQ*) were individually expressed in *S. oneidensis* to regulate intracellular c-di-GMP, and the results showed that *yedQ* led to the highest c-di-GMP accumulation. Finally, these three genes (*mxdB*, *pilA*, and *yedQ*) were assembled into the *S. oneidensis speF* mutant to construct an engineered strain, EnBF2, which exhibited enhanced biofilm formation and EET efficiency. By integrating carbon nanotubes with the EnBF2 biofilms, the electrical conductivity was markedly improved. Overall, these works demonstrate that biofilm engineering provides a feasible platform for biosynthesis in the microbe–semiconductor biohybrid systems.

## 5 Characterizations of Microbe–Semiconductor Interfacial Electron Transfer

As discussed in the previous sections, designing a favorable biohybrid interface is crucial for achieving efficient solar energy conversion,^106,264^ and it is thus essential to fully elucidate the interfacial interactions in biotic–abiotic hybrid systems.^200^ Currently, there are many techniques and characterizations which can provide detailed information on interfacial electron transfer between microbes and semiconductors.^265^ For example, time-resolved photoluminescence (TRPL) and transient absorption spectroscopy (TAS) can help us understand the charge transfer dynamics of biohybrid interfaces at the time scale of femtoseconds and nanoseconds.^13,266^ Photoelectrochemical characterizations like electrochemical impedance spectrum (EIS) and transient photocurrent tests can unravel the basic behaviors of photogenerated charge in the biohybrid systems.^267^ Another promising characterization is operando imaging technique, which can probe interfacial electron transfer at the single- or subcellular levels.^268,269^ In addition, genomics analysis and proteomics analysis will also contribute to exploring the genes and proteins related to EET pathways and microbial metabolism.^202,270^ In this section, we will summarize the relevant characterizations that involve biohybrid interfacial electron transfer,. These characterizations can be divided into optical characterization, photoelectrochemical characterization, operando imaging technique, and omics analysis. The combination of these characterizations and techniques can offer the comprehensive information on charge transfer dynamics at the microbe–semiconductor interfaces.^271^

### 5.1 Optical Characterization

Spectroscopic techniques are the basic characterizations to understand the charge behaviors in microbe–semiconductor biohybrid systems, such as charge separation, charge transfer, and charge recombination.^272^ In these systems, upon photon absorption, the semiconductor photocatalysts generate electrons and holes in the CB and VB, respectively. The photogenerated electrons must migrate to the semiconductor surface and subsequently transfer across the biohybrid interfaces to be utilized by microbial metabolism.^17^ By employing optical characterizations like photoluminescence (PL) spectroscopy and TAS, we can obtain the semiconductor’s photophysical properties to help us understand the complex processes of charge transfer dynamics in the biohybrid systems.

#### 5.1.1 Photoluminescence Spectroscopy

When a semiconductor captures photons that surpass the energy of its bandgap, it will excite electrons from VB to CB, thus leaving corresponding holes in the VB. The excited electrons can also relax back to a lower energy level, and then recombine with holes to emit light, which is called PL.^273^ PL analysis has been commonly used in optical research to characterize the recombination of photogenerated electrons and holes.^48^ Both the PL intensity and wavelength of emitted photons can be measured in the PL spectrum. Generally, the higher the recombination rate, the lower the corresponding photogenerated carrier separation rate. For a steady-state PL spectrum, it is generally believed that the high PL peak intensity represents a low photogenerated carrier separation efficiency.^34^ Apart from using the steady-state PL spectrum, a TRPL spectrum that measures the time dependence of the PL intensity can provide the lifetimes of charge carriers.^274,275^ The time required for the fluorescence intensity to decay to 1/*e* of the initial intensity is the PL lifetime. Hence, after the integration of semiconductors and bacteria, we can test the PL intensity and PL lifetime to investigate interfacial electron transfer processes in the microbe–semiconductor biohybrids.

For example, Luo and co-workers used PL spectroscopy to study the electron transfer behavior of CuInS_2_/ZnS QDs–*S. oneidensis* biohybrid system.^198^ In contrast to CuInS_2_/ZnS QDs, the PL intensity of QDs–*S. oneidensis* biohybrids with hydrogenase deletion decreased slightly, whereas a pronounced quenching was observed in the QDs–*S. oneidensis* biohybrids with wild type cells. This result not only indicated that the combination of semiconductor QDs and *S. oneidensis* could promote electron transfer from QDs to *S. oneidensis* but also supported the notion that the hydrogenases in *S. oneidensis* could function as an electron acceptor to suppress the recombination of electrons and holes. TRPL spectra showed that the PL lifetime of QDs–*S. oneidensis* was 62 ns, shorter than that of CuInS_2_/ZnS QDs (266 ns), further supporting that photogenerated electrons were transferred to hydrogenases to facilitate H_2_ metabolism.

Guan et al. also tested steady-state PL intensity and PL lifetime to study interfacial charge transfer dynamics in the CdTe QDs–*Xanthobacter autotrophicus* (*X. autotrophicus*) biohybrid system (Figure 18a).^200^ From Figure 18b and Figure 18c, it can be observed that pure CdTe QDs exhibit strong and stable PL with a long lifetime (49.4 ± 0.5 ns). Adding *X. autotrophicus* or cysteine individually could cause only minor quenching. Surprisingly, when combining QDs, microorganisms, and cysteine together, the PL intensity displayed a significant decline together with a shortest lifetime (4.4 ± 0.3 ns), indicating that rapid charge transfer occurred at the microbe–semiconductor interface. Notably, QD aggregation could not affect the PL properties, which demonstrated that this enhanced charge transfer arises from a gradually forming interface facilitated by the assembly of QDs, microorganisms and cysteine together. Besides, the charge transfer rate constant (*k*_ET_ = 2.1 × 10^8^ s^–1^) surpasses all other decay pathways, supporting highly efficient migration of photogenerated electrons to the *X. autotrophicus*. As shown in Figure 18d, flow cytometry confirmed that cysteine played a main role in the charge extraction from CdTe QDs to microbes. Finally, Stern–Volmer analysis showed that QDs with cysteine showed dynamic quenching (Figure 18e,f), while the biohybrid systems with cysteine exhibited combined dynamic and static quenching. This static quenching is key to the high *k*_ET_ and efficient carrier transfer at the hybrid interfaces, underpinning the high internal quantum yields of this microbe–QDs biohybrid system for efficient photocatalytic applications.

**Figure 18:**
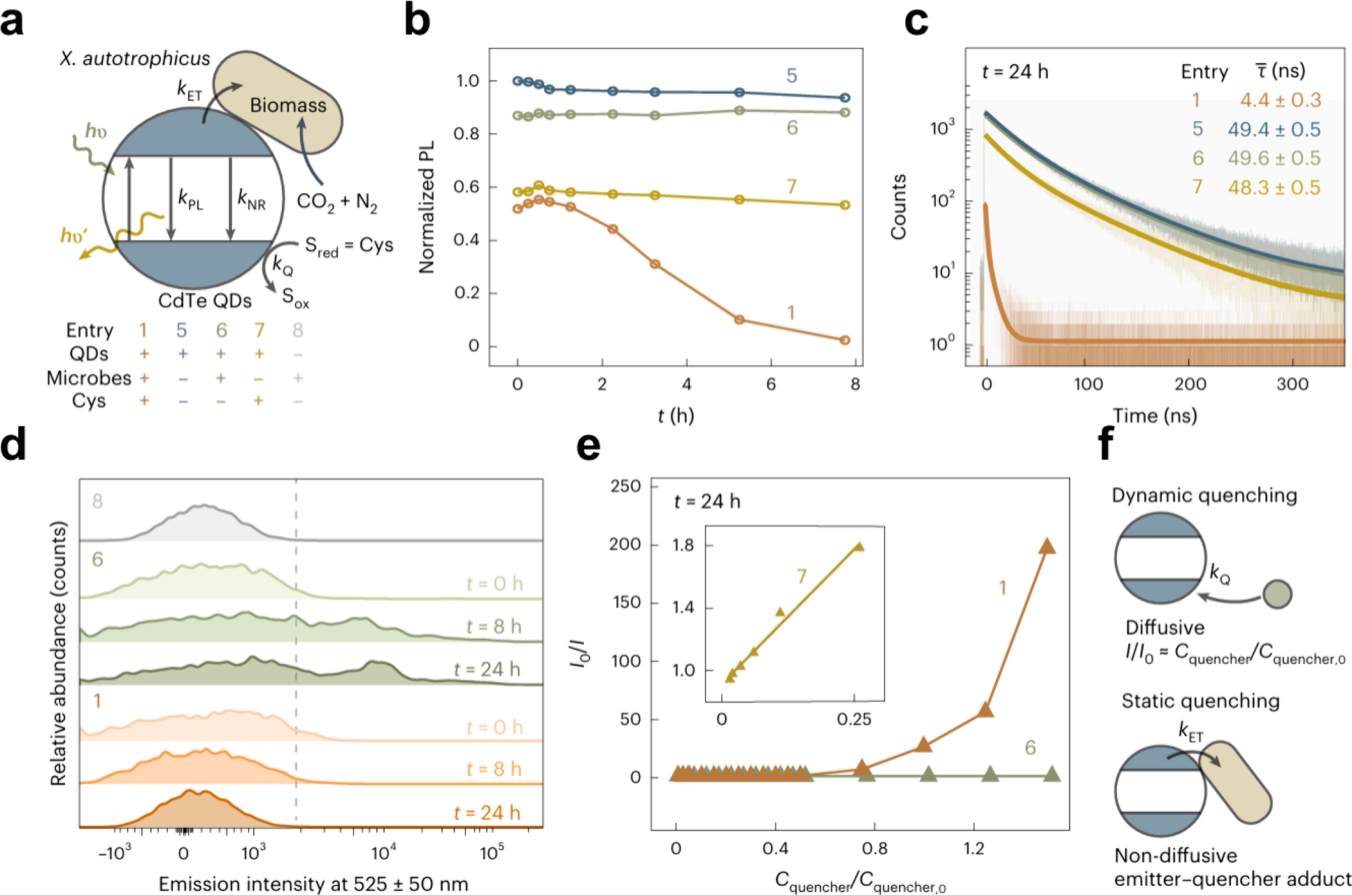
Photophysical characterizations at the microbe–QDs interface. (a) The photophysical processes in the photocatalytic hybrid (entry 1) and other controls (entries 5–8). (b) Normalized PL intensity versus time after assembly under different conditions. (c) The lifetime of photoexcited states at *t* = 24 h as determined by time-correlated single-photon counting. (d) The time-correlated population distributions of *X. autotrophicus* bound with QDs under different conditions as determined by flow-cytometry experiments. The populations on the right side of the dashed line suggest microorganisms bound with emissive QDs. (e) Stern–Volmer analysis plotting the inverse of normalized PL intensity (*I*_0_/*I*) versus the relative equivalence of quenchers (*C*_quencher_/*C*_quencher,0_) at *t* = 24 h. (f) Schematic illustration of dynamic quenching and static quenching. Reproduced with permission from from ref 200. Copyright 2022 Springer Nature.

#### 5.1.2 Transient Absorption Spectra

Apart from PL and TRPL spectra, TAS is another powerful optical technique to probe the excited-state charge dynamics of semiconductors.^273,276^ In a typical transient absorption experiment, a short laser pulse first excites a sample in the ground state to the excited states, followed by a weak probe pulse to monitor the absorption changes.^277^ The absorbance differences before and after excitation is then recorded as the signals in the TAS.^278^ By analyzing these signals in different time scales, TAS can provide deep insight into the relaxation times and charge transfer processes of semiconductors under excitation.^279^ In the biohybrid systems, TAS can also help us unlock charge dynamics at the microbe–semiconductor interfaces, providing the microscopic cognition of electron transfer at the time scale of femtoseconds or nanoseconds.

For example, Wen and colleagues utilized TAS to analyze the charge transfer process in the InP/ZnSe/ZnS QDs/*S. ovata* biohybrid systems.^13^ As shown in Figure 19a–c, compared with pristine InP/ZnSe/ZnS QDs, the QDs/*S. ovata* biohybrid system showed faster decay kinetics, attributed to the effective electron transfer from QDs to the intracellular hydrogenases in *S. ovata*. Further addition of K_3_Fe­(CN)_6_ as an electron transfer mediator, the *S. ovata*/QDs–K_3_Fe­(CN)_6_ biohybrid system exhibited faster decay dynamics than that of *S. ovata*/QDs biohybrid system, indicating that the electron transfer mediator could further facilitate electron transfer in this biotic–abiotic hybrid system. This phenomenon was further confirmed by the shortest decay time of the *S. ovata*/QDs–K_3_Fe­(CN)_6_ biohybrids (Figure 19d). Therefore, this result demonstrated that the photogenerated electrons could effectively transfer from QDs to *S. ovata* for enhanced photosynthetic metabolism.

**Figure 19:**
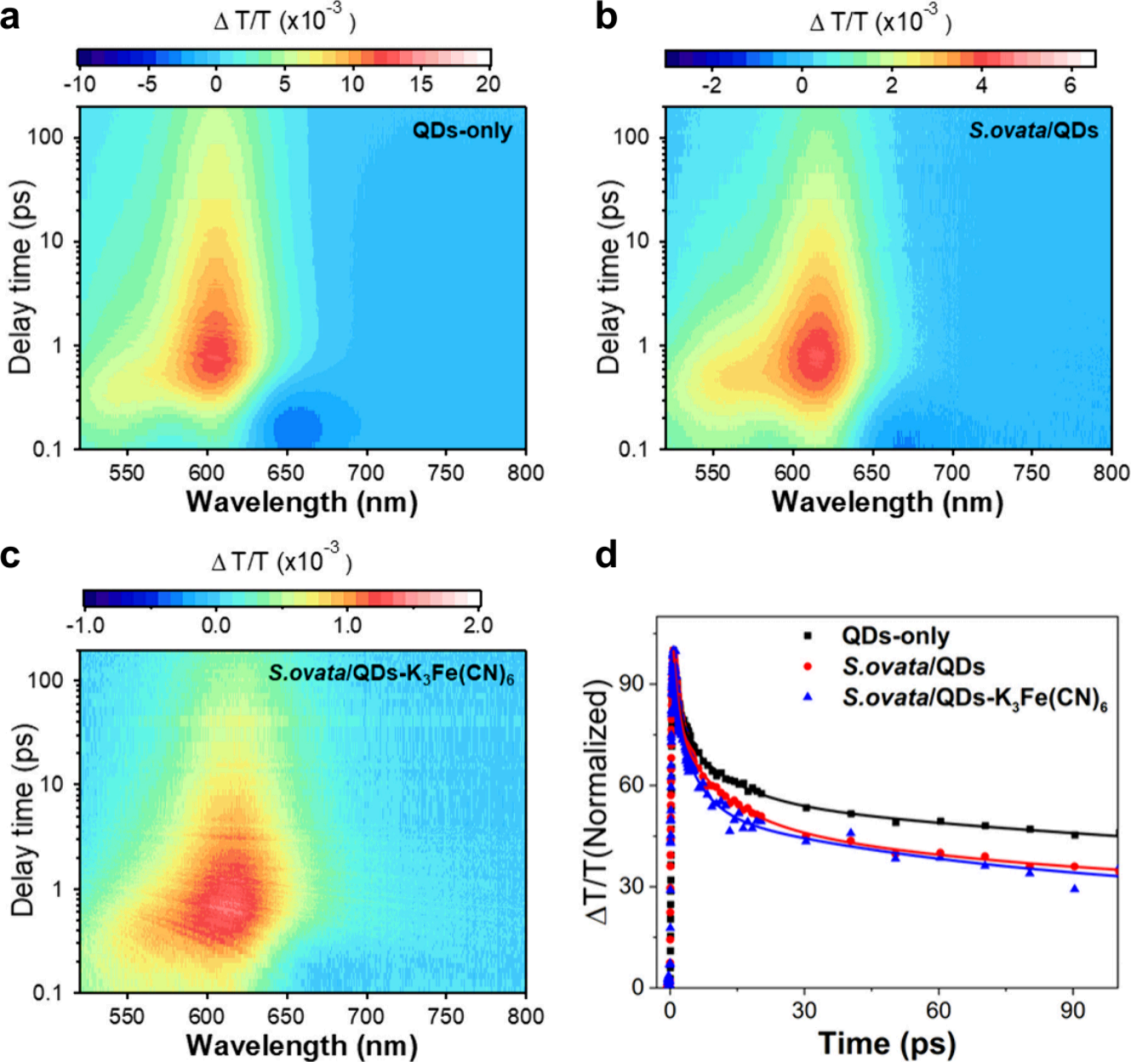
TAS of the InP QDs-only (bacteria-free) and *S. ovata*/InP QDs hybrid system with or without K_3_Fe­(CN)_6_. (a) TAS of QDs-only; (b) TAS of *S. ovata*/InP QDs without K_3_Fe­(CN)_6_. (c) TAS of *S. ovata*/InP QDs with K_3_Fe­(CN)_6_. (d) TA decay kinetics of the InP QDs-only (bacteria-free) and *S. ovata*/InP QDs hybrid system with or without K_3_Fe­(CN)_6_. Reproduced with permission from ref 13. Copyright 2022 Elsevier.

In short, PL, TRPL, and TAS are very powerful characterizations to measure the photophysical properties of microbe–semiconductor biohybrid systems, and these tests can straightforwardly help us uncover electron flow at the biotic–abiotic interfaces.^13,280^ However, some challenges also exist by using these optical techniques to understand biohybrid interfacial electron transfer. Some factors such as solvent effects, light source, and other material disturbances would affect the accuracy of optical signals.^273^

### 5.2 Photoelectrochemical Characterization

Photoelectrochemical tests like EIS and transient photocurrent response can also be carried out to measure photogenerated carrier separation and transfer abilities.^281^ EIS is a commonly used method to characterize charge transfer dynamics at the microbe–semiconductor interfaces.^282^ To be specific, EIS is an electrochemical technique that is used to study the resistance and capacitive behavior by applying different alternating current voltages. The resulting current response is measured over a wide range of frequencies (usually from MHz to mHz). Afterward, we can obtain quantitative information on solution resistance, charge transfer resistance, capacitance, and inductance from Nyquist plot or Bode plot. In the microbe–semiconductor biohybrid systems, EIS spectra can also be utilized to investigate the electron transfer dynamics at the biohybrid interfaces.

In the PEDOT-S–*Synechococcus elongatus* (*S. elongatus*) biohybrids, the increasing concentration of PEDOT-S possessed the smaller semicircle diameters, indicating that PEDOT-S, functioning as a conductive matrix, could decrease electron transfer impedance at the biohybrid interfaces (Figure 20a).^283^ Moreover, after adding K_3_[Fe­(CN)_6_] as the mediator (Figure 20b), the electron transfer impedance was further reduced with the 2 mg mL^–1^ PEDOT-S as the optimal concentration to combine with *S. elongatus*. In an NDI-based organic polymer p­(ziNDI-gT2)–*S. oneidensis* biohybrid system, EIS measurements were conducted to examine the charge transfer behaviors in this biocomposite.^284^ Both the p­(ziNDI-gT2)–*S. oneidensis* biocomposite and pure p­(ziNDI-gT2) exhibited typical semicircles of interfacial faradaic charge transfer. Further fitting results of EIS spectra revealed that electron transfer impedance was decreased from 4 to 2 Ω at the biotic–abiotic hybrid interfaces (Figure 20c), demonstrating favorable charge transfer in the p­(ziNDI-gT2)–*S. oneidensis* biocomposite. In another typical conjugated polyelectrolyte (CPE)–*S. oneidensis* biohybrid system, the EIS plot of biohybrids showed the lowest electron transfer resistance than that of CPE and *S. oneidensis*.^282^ This improved electrochemical property revealed that the assembly of organic polymers and microbes could promote electron transfer at the biohybrid interfaces.

**Figure 20:**
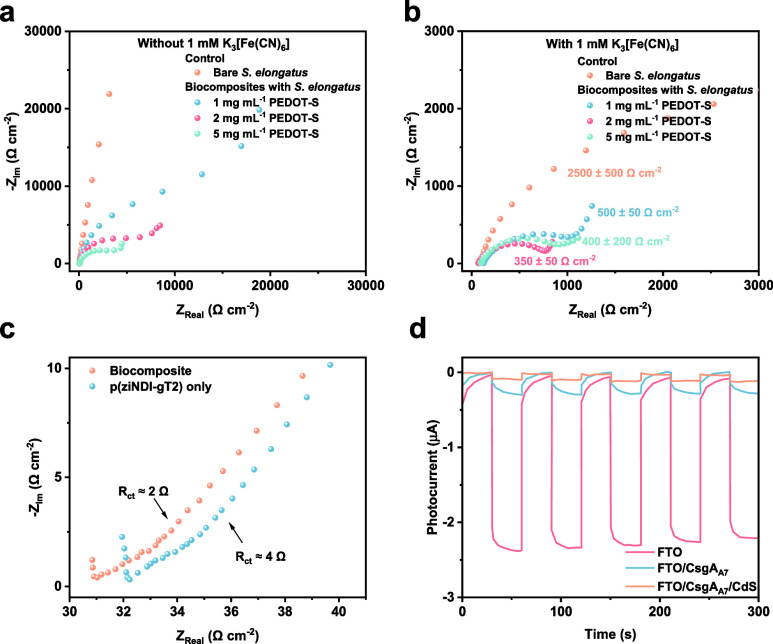
Photoelectrochemical characterizations of biohybrid systems. (a) Representative Nyquist plots for *S. elongatus* and PEDOT-S–*S. elongatus* biocomposites under mediator-less conditions. (b) Representative Nyquist plots for *S. elongatus* and PEDOT-S–*S. elongatus* biocomposites under mediator conditions. (c) Enlarged EIS spectra of the biohybrid and p­(ziNDI-gT2) with annotated values showing the charge-transfer resistance. (d) Transient photocurrent curves of FTO, FTO/CsgA_A7_, and FTO/CsgA_A7_/CdS biohybrids. FTO: fluorine doped tin oxide-conductive glass; CsgA_A7_: CsgA protein binding to A7 peptides. Reproduced with permission from ref 283. Copyright 2025 Springer Nature. Reproduced with permission from ref 284. Copyright 2022 Wiley-VCH. Reproduced with permission from ref 285. Copyright 2022 American Association for the Advancement of Science.

Transient photocurrent *i*–*t* tests can also unravel interfacial photogenerated charge transfer dynamics at the microbe–semiconductor interfaces. Wang et al. conducted the transient photocurrent test of FTO/CsgA_A7_/CdS biohybrids to understand the behavior of photoinduced electrons from CdS nanoparticles within photocatalyst-mineralized biofilms (Figure 20d).^285^ The A7 peptide is a cysteine-rich metal-binding motif that is genetically fused to the *E. coli* curli subunit CsgA protein, and the formed CsgA_A7_ nanofibers have a strong affinity toward transition metal ions for in situ mineralization of CdS nanoparticles. In contrast to FTO and FTO/CsgA_A7_, the highest photocurrent of FTO/CsgA_A7_/CdS biohybrids indicated that the biohybrids possessed superior charge separation and transfer abilities at the biohybrid interfaces. Similarly, compared to FTO and FTO-biofilms, the highest photocurrent response of CdS-mineralized biofilms supported the effective photogenerated electron transfer at CdS-biofilms interfaces.^261^

### 5.3 Operando Imaging Technique

Optimizing interfacial charge transfer is crucial for improving the efficiency of photocatalytic and photoelectrocatalytic processes.^286^ The underlying mechanism of interfacial electron transfer is very important for biotic–abiotic hybrid systems. Nonetheless, previous characterizations have investigated the charge transfer kinetics from bulk measurements. Due to the ubiquitous heterogeneity of microbes and semiconductors, it is necessary to acquire the concrete information on charge transfer at the single-particles/single-cells levels.^287^ Recently, the emerging operando imaging technique has shown great promise in measuring the local functional information such as photocurrents and PL spectra at the single-particle/single-cell level.^268,288^ Through finding the relationships between the local functional signals and the specific interfacial structure, a quantitative and detailed understanding of the biotic–abiotic hybrid systems can be realized, functioning as a foundation to guide the design of biohybrid interfaces for solar energy conversion.^37^

Fu and co-workers have developed a quantitative operando imaging technique to investigate the electron uptake mechanism of biohybrids at the single-particle and single-cell levels.^269^ Previously, the lithoautotrophic Gram-negative bacterium *C. necator* has been used in decoupled biohybrids (Figure 21a, top) that used H_2_ via DET pathway to fix CO_2_ into the PHB. Through combining different semiconductors (Cu_2_WS_4_, BiVO_4_, and CdS) and *C. necator* to build different biotic–abiotic hybrid systems, the operando imaging technique discovered that *C. necator* could directly uptake electrons from semiconductor photoelectrodes with nanoampere-level currents (Figure 21a, bottom and Figure 21b), and the interfacial electron transport was not mainly mediated by H_2_. This result indicated that *C. necator* could also be utilized as integrated biohybrids interfacing with semiconductors, although hydrogenases were very essential in yielding photoelectrochemical currents. This operando imaging approach offers a new option to unlock electron uptake mechanism at the biohybrid interfaces and enlightens more opportunities to improve the efficiency of converting solar energy into chemical energy.

**Figure 21:**
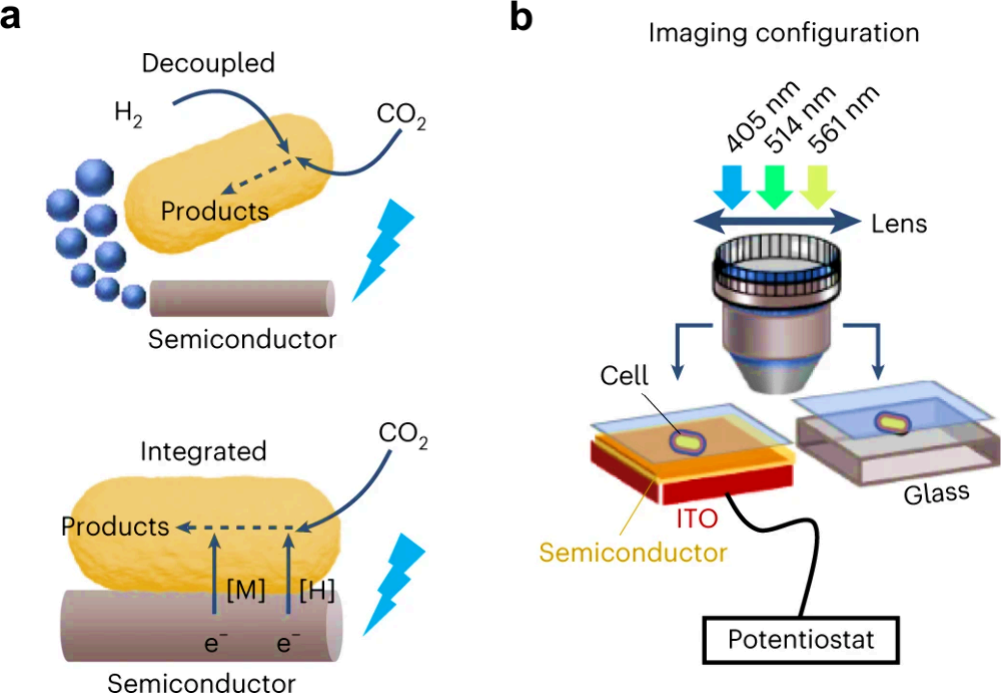
Decoupled and integrated microbe–semiconductor biohybrid systems and operando imaging technique. (a) Decoupled and integrated biohybrids for energy conversion. (top) H^+^/H_2_ as the redox mediator; (bottom) direct interfacial electron transfer. (b) Multimodal operando imaging platform: a (photoelectrochemical) microfluidic cell on an epifluorescence microscope with multiple and modulated lasers. Reproducedwith permission from ref 269. Copyright 2023 Springer Nature.

Recently, our group reported a strategy to promote electron uptake rate by constructing single-atom bridges at the biotic–abiotic interfaces, thereby enhancing STC conversion efficiency.^12^ The porous C_3_N_4_ loaded with Ru atoms (designated as C_3_N_4_/Ru) was integrated with *S. oneidensis* to build a C_3_N_4_/Ru–*S. oneidensis* biohybrid system, as shown in the atomic force microscopy (AFM) image (Figure 22a). Then, operando single-cell/single-particle photocurrent tests were performed to quantify the capacity of *S. oneidensis* to take up electrons from semiconductors. First, the photocurrent density differences between C_3_N_4_ and C_3_N_4_–*S. oneidensis* was recorded as Δi_1_ (Figure 22b), representing the ability of *S. oneidensis* receiving electrons from C_3_N_4_. Then, the photocurrent density difference (Δ*i*_2_) between C_3_N_4_/Ru and C_3_N_4_/Ru–*S. oneidensis* was also measured (Figure 22c), indicating the capacity of *S. oneidensis* to uptake electrons from C_3_N_4_/Ru nanosheets. Compared to Δ*i*_1_, Δ*i*_2_ exhibited a 3.0-fold enhancement of single-cell photocurrent density (Figure 22d), demonstrating that single atoms at the interfaces could effectively promote interfacial electron transfer from semiconductors to bacteria.

**Figure 22:**
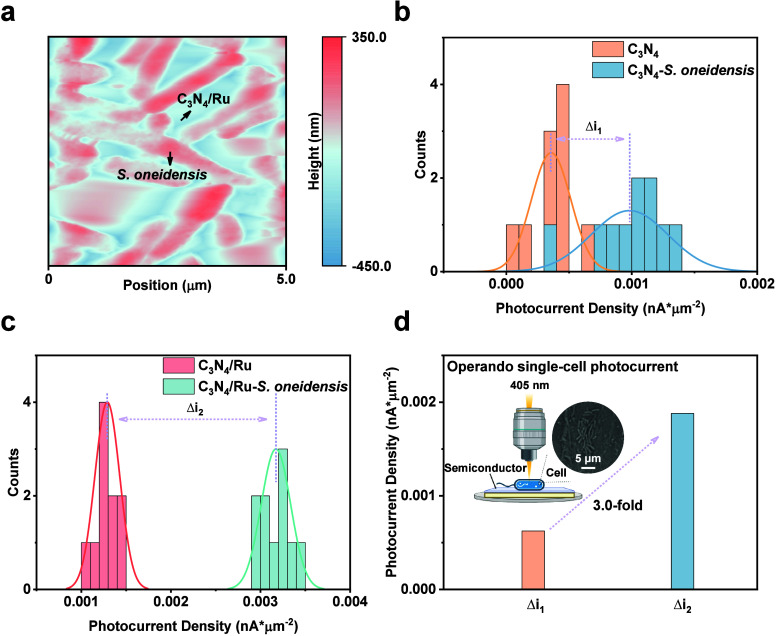
Operando single-cell interfacial electron uptake in porous C_3_N_4_/Ru–*S. oneidensis* biohybrid system. (a) AFM image of C_3_N_4_/Ru–*S. oneidensis* biohybrids. (b) Histograms of single-cell/single-particle photocurrent density of C_3_N_4_ and C_3_N_4_–*S. oneidensis* as well as the photocurrent density differences marking as Δ*i*_1_. (c) Histograms of single-cell/single-particle photocurrent density of C_3_N_4_/Ru and C_3_N_4_/Ru–*S. oneidensis* as well as photocurrent density differences marking as Δ*i*_2_ (d) The photocurrent density differences of Δ*i*_1_ and Δ*i*_2_. Inset: scheme of operando single-/subcell photocurrent technique. Reproduced with permission from ref 12. Copyright 2025 Springer Nature.

In conclusions, operando imaging technique has become one of the most powerful tools to unravel the structure–functional relationships from single-cell/single-particle levels.^289^ This operando imaging technique sheds light on promising insight into the underlying mechanism of charge transfer, while typical bulk measurements cannot achieve such in-depth understanding of electron behaviors at the biotic–abiotic biohybrid interfaces.^290^

### 5.4 Omics Analysis

To fully grasp microbial metabolism and its environmental responses, it is necessary to analyze their genes and investigate gene products, including mRNA, proteins, and metabolites.^291^ Over the past few decades, the requirements of these analyses have led to the development of various omics analyses, such as genomics, transcriptomics, proteomics, and metabolomics. The omics analysis refers to the comprehensive, high-throughput, and data-driven investigations of the cellular metabolism.^292^ These omics approaches utilize advanced statistical and computational techniques to analyze large amounts of data sets, which can offer a systematic-level study of microbial metabolism and expression.^293^

Genomic analysis involves the identification, measurement, and comparison of genomic features, encompassing the complete set of genetic information in an organism.^294,295^ However, genomics alone cannot capture the dynamic regulation of biohybrid interfacial electron transfer and metabolism in biohybrid systems; therefore, complementary transcriptomics, proteomics, and metabolomics analyses are required. Transcriptomics provides us a detailed view of gene expression changes induced by photoelectrochemical or redox stimuli, enabling the identification of responsive pathways and regulatory networks.^296^ However, transcript levels do not necessarily correlate with the protein abundance and activity. Proteomics directly probes protein expressions by identifying and quantifying specific proteins, offering insights into the functional machinery involved in electron transfer and metabolic activity. Nevertheless, it is often limited by proteome coverage and the challenges in detecting low-abundance proteins.^297^ Metabolomics generally captures the downstream metabolic consequences in a system (e.g., cells, tissues, organisms), which can reflect the result of intracellular redox balance and metabolic products.^298^ As the metabolome represents the final downstream product of gene transcription and protein activity, changes in metabolite levels are often amplified relative to changes in the transcriptome and proteome. However,interpreting metabolomic data can be complicated by metabolic redundancy and rapid metabolite turnover.^295,299^

In the field of semibiological photosynthesis, these different omics analyses can also provide us with comprehensive knowledge of genes, proteins, and metabolic activity.^201,270^ For instance, electroactive bacteria like *S. oneidensis* possess well-established EET pathways that can support both DET and MET.^300,301^ During the photosynthetic processes, by comparing the expressed abundances of relative proteins and metabolites using proteomics and metabolome, we can easily deepen the insight of external stimuli on interfacial electron transfer and microbial metabolism. Notably, a single omics technique cannot fully elucidate the complexity of microbe–semiconductor biohybrids. Integrating these omics analyses together will be effective to help us elucidate cellular metabolic activity and further unravel biohybrid interfacial electron transfer. Such multiomics approaches therefore provide comprehensive information for the rational design and optimization of semibiological photosynthesis.

Specifically, to probe the microbial metabolisms during the cofixation of CO_2_ and N_2_ in the CdTe–*X. autotrophicus* biohybrids, proteomic and metabolomic analyses have been conducted, using pure *X. autotrophicus* autotrophically grown with H_2_ as the control group.^200^ A total of 2743 proteins and 110 metabolites were clearly identified, with principal component analysis (PCA) and volcano plots revealing obvious differences between the biohybrid group and H_2_-fed groups. Results showed that the enzymes related to nitrogen fixation and the Calvin–Benson–Bassham (CBB) cycle were significantly upregulated (Figure 23a), supporting the high quantum efficiency of N_2_/CO_2_ fixation in photocatalysis. Meanwhile, the HupV protein was also upregulated in the biohybrid system, which indicates the absence of H_2_ in the biohybrid system, because HupV expression is repressed by H_2_. This result suggests a direct and nondiffusive electron transfer at the microbe–semiconductor interfaces. Meanwhile, the proteins involved in redox signaling and electron transport, such as the RegA protein and upstream complexes I and II, were also upregulated, whereas downstream complex IV and ATP synthase were significantly downregulated. These observations were consistent with lower ATP concentration, increased amino acids, and nucleotide accumulation (Figure 23b). The 4.4-fold increase in nitrogen-containing biomolecule concentrations indicated the efficient ATP use for biosynthesis. This result highlighted the potential of proteomic and metabolomic analyses in understanding the conversion processes in the microbe–semiconductor biohybrid systems.

**Figure 23:**
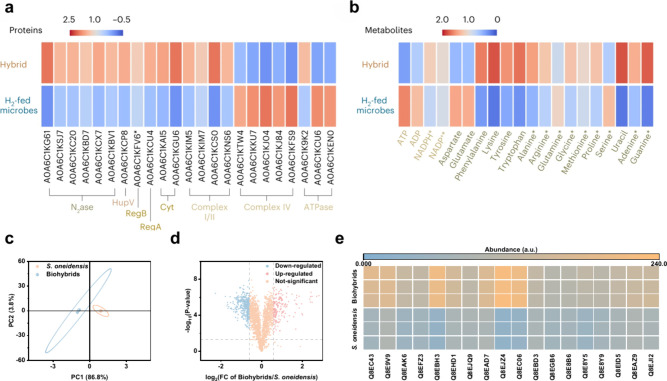
Proteomic and metabolomic analyses. CdTe–*X. autotrophicus* biohybrids: (a) Heatmaps of significantly regulated proteins; (b) heatmaps of significantly regulated metabolites. C_3_N_4_/Ru–*S. oneidensis* biohybrids: (c) PCA plot and (d) volcano plot; circles in (c) mean 95% confidence; lines in (d) represent the gating of fold change (FC) of biohybrids/*S. oneidensis* >1.5 or <0.66 and *P* < 0.05 from a two-tailed *t* test. (e) Heatmaps of significantly up-regulated proteins. Proteins and relevant Gene ID. Protein related to flavin synthesis: Q8EC43, Q8E9V9. Outer membrane: Q8EAK6, Q8EFZ3, Q8EBH3, Q8EHD1, Q8EJQ9, Q8EAD7, Q8EJZ4, Q8EC06, Q8EBD3. ATPase: Q8EGB6, Q8E8B6. Cytochrome: Q8E8Y5, Q8E8Y9. NAD­(P)H reductase: Q8EID5, Q8EAZ9. Ferredoxin: Q8EJI2. Reproduced with permission from ref 200. Copyright 2022 Springer Nature. Reproduced with permission from ref 12. Copyright 2025 Springer Nature.

In our recent work, to further explore whether single-atom bridge at the microbe–semiconductor interfaces could affect the metabolism and charge transfer pathways, we have also conducted proteomic analysis in the C_3_N_4_/Ru–*S. oneidensis* biohybrid system.^12^ 2622 proteins were identified and the differences of protein expressions in Shewanella and C_3_N_4_/Ru–*S. oneidensis* biohybrids were confirmed by PCA plot (Figure 23c). In contrast to *S. oneidensis*, the volcano plot of the biohybrids showed that 210 proteins were upregulated (Figure 23d), most of which were related to EET pathways and H_2_ metabolism, such as flavin synthesis, cytochromes, and outer membrane proteins (Figure 23e). These upregulated proteins facilitated effective electron uptake and intracellular hydrogen production in the C_3_N_4_/Ru–*S. oneidensis* biohybrid system.

To understand the underlying mechanism of enhanced N_2_ reduction in PFP/*A. Chroococcum* biohybrid systems, genetic analysis and relative protein expression were studied. Nitrogenase, the key enzyme for N_2_ fixation, consists of Fe proteins (encoded by nifH) and MoFe proteins (encoded by nifD and nifK) (Figure 24a).^202^ It could be observed that nifD and nifK were significantly upregulated by 58% and 44%, respectively, while the nifH expression stayed the same (Figure 24b). This result indicated that the photogenerated electrons from PFP mainly enhanced MoFe protein activity and further improved the N_2_ fixation efficiency of *A. Chroococcum*. Protein analysis (Figure 24c) further showed that multiple N_2_ fixation-related proteins were obviously upregulated, including different subunits of nitrogenase and other related proteins (e.g., nitrogenase cofactor biosynthesis protein and nitrogenase protein alpha chain).

**Figure 24:**
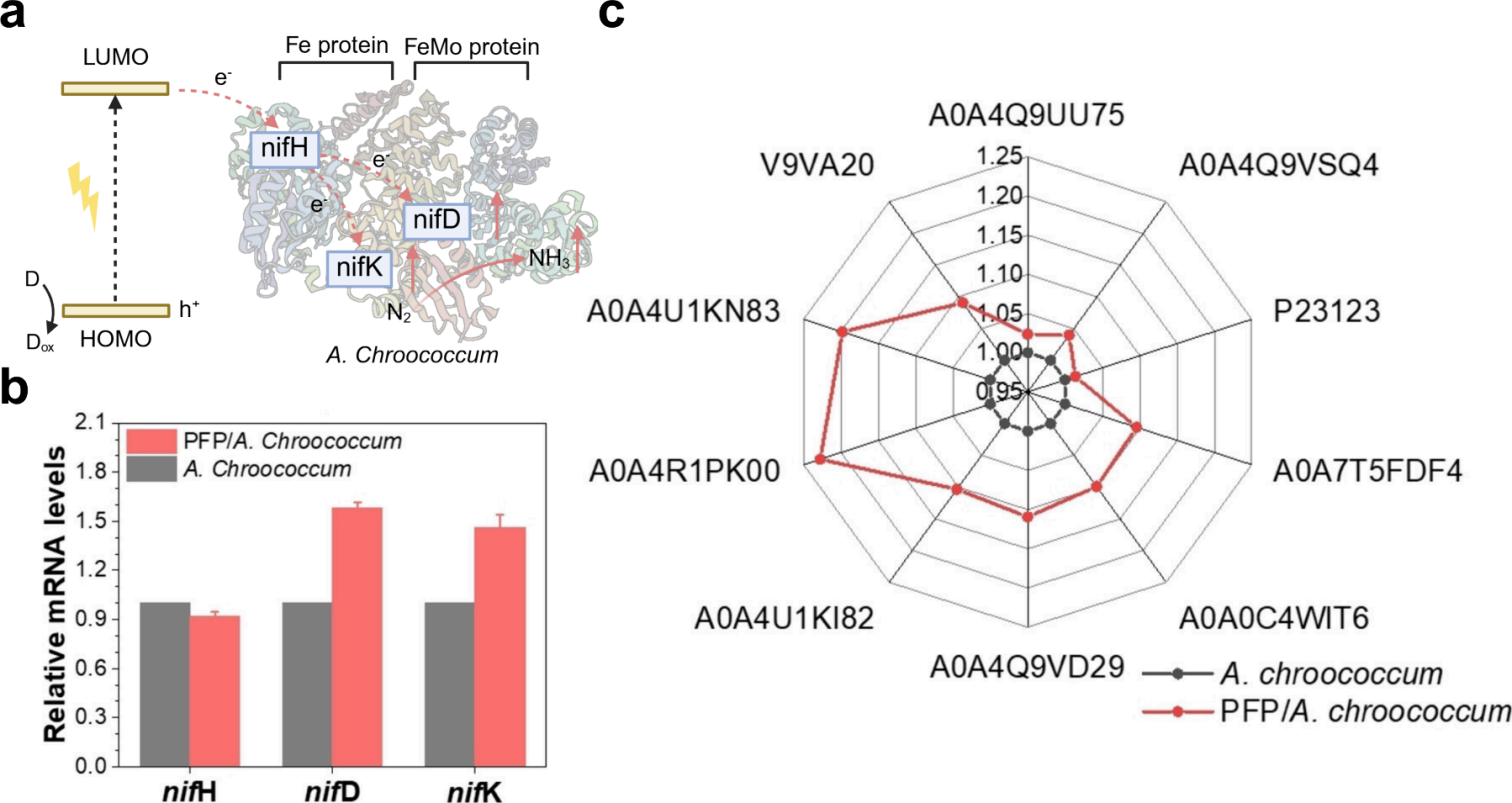
Genomic analysis of PFP/*A. Chroococcum* biohybrid system for photocatalytic N_2_ reduction. (a) Scheme of the relative nif genes in nitrogenase. (b) Relative mRNA expression level of nifH, nifD and nifK. Multiple *t* tests were conducted to analyze the data. *p < 0.05, **p < 0.01. (c) Spider plot of the expression level of proteins associated with N_2_ fixation. Reproduced with permission from ref 202. Copyright 2023 Wiley-VCH.

Omics analysis is indeed a very straightforward and comprehensive approach to gaining insightful information on biological metabolic processes and interfacial electron transfer in the microbe–semiconductor biohybrids.^37,302^ Due to the high-throughput nature of the data and the complexity of metabolic activities, it requires patience to elucidate the relationships between the data and the corresponding metabolic pathways.^303^

## 6 Application of Microbe–Semiconductor Biohybrid Systems

### 6.1 Semibiological Photosynthesis

Recent advancements have highlighted the potential of harnessing microbial metabolism for solar-driven chemical synthesis through photocatalysis and photoelectrocatalysis. Previous reviews have primarily concentrated on converting stable feedstocks such as H_2_O, CO_2_, and N_2_ into valuable products.^17,37^ In this section, we shift focus from general overviews to specific examples that illustrate how rational engineering of the microbe–semiconductor interfaces can enable efficient photoelectron transfer into target enzymes or metabolic pathways for semibiological photosynthesis. These typical semibiological photosynthesis systems for STC conversion are summarized in Table 3.

**Table 3:** Summary of Typical Semibiological Photosynthesis Systems for STC Conversion

microbes	semiconductors	reactions	interface assembly	performance	operation time	efficiency	ref
*E. coli*	TiO_2_	H_2_ production	MV mediator	H_2_: 3.6 mmol/mmol glucose in 15 h	15 h		^308^
*R. palustris*	OF/PTP	H_2_ production	MV mediator	H_2_: 624.80 nmol/30 min	2 h		^309^
*S. oneidensis*	CdS/PPy	H_2_ production	PPy coating	H_2_: 23.18 μmol 10^9^ cells^–1^ day^–1^	15 d		^311^
*S. oneidensis*	C_3_N_4_/Ru	H_2_ production	Ru atom	H_2_: 18.6 μmol g^–1^ h^–1^	80 h	8.46% at 450 nm	^12^
*E. coli*	eosin Y	H_2_ production	intracellular	H_2_: ∼0.5 μmol mL^–1^ OD_600_^–1^ in 24 h	24 h		^312^
*E. coli*	g-C_3_N_4_ QDs	H_2_ production	intracellular	H_2_: 7800 μmol g^–1^ h^–1^	50 h		^241^
*S. oneidensis*	CuInS_2_/ZnS	H_2_ production	intracellular	H_2_: 491.8 μmol in 9 h	45 h	15.02% at 475 nm	^198^
*S. ovata*	Cr_2_O_3_/Ru-SrTiO_3_: La, Rh|ITO|RuO_2_–BiVO_4_: Mo	CO_2_ reduction to acetate	photocatalyst sheet	CH_3_COOH: 9 mM in 15 h	45 h	0.70% for STC	^317^
*M. barkeri*	CdS	CO_2_ reduction to CH_4_	NiCu alloy	CH_4_: 79.38 ± 2.83 μmol g_cat_^–1^ h^–1^	15 d	12.41% for 395 nm	^220^

engineered *E. coli*	CdTe QDs	CO_2_ reduction to formate and pyruvate	intracellular	formate: 0.65 g L^–1^	8 h		^318^
				pyruvate: 0.18 g L^–1^ in 8 h			

*M. thermoacetica*	Au nanoclusters	CO_2_ reduction to acetate	itracellular	CH_3_COOH: 6.01 mmol g^–1^ in 7 d	7 d	2.86% for STC	^242^
*S. ovata*	Si nanoarrays	CO_2_ reduction to acetate	Si nanoarrays	CH_3_COOH: 6.0 g L^–1^ in 5 d	200 h	0.38% for STC	^127^

*A. chroococcum*	PFP	N_2_ fixation to ammonia	electrostatic interaction	ammonium nitrogen: 1.40 μg/10^9^ cell	48 h	0.83% for STC	^202^
				l-amino acids: 4.51 nmol/10^9^ cell			

*A. vinelandii*	COE-IC	N_2_ to ammonia	electrostatic interaction	NH_3_: 20.6 nmol mL^–1^ in 14 h	24 h		^325^
*A. vinelandii*	InP/ZnSe QDs	N_2_ to ammonia	intracellular	NH_3_: 14 × 10^7^ mol NH^4+^/mol CFU	10 h		^326^
*A. vinelandii*	NiO/PDA	N_2_ to ammonia	PDA layer	NH_3_: 1.85 μmol h^–1^/10^8^ cells	72 h		^219^
*A. vinelandii*	Cu_2_O@TiO_2_	N_2_ to ammonia	Cu_2_O@TiO_2_ nanowires	NH_3_: 1.49 × 10^–9^ mol s^–1^ cm^–2^	24 h	0.43% for STC	^185^
*C. necator*	carbon nitride nanosheets	CO_2_ reduction to PHB	self-assembly	PHB: 37.25 mg L^–1^ day^–1^	6 d	5.81% at 583.2 nm	^8^
*R. eutropha*	PBDTTT-CP/PDI-CP	CO_2_ reduction to PHB	electrostatic interaction	PHB: 107.3 mg L^–1^ OD_600_^–1^	5 d	1.14% for STC	^331^
*S. cerevisiae*	InP QDs	shikimic acid	self-assembly	shikimi: 8.5 mg L^–1^	72 h	1.58% for STC	^332^
*M. barkeri*	carbon nitrides	CO_2_ and PLA to CH_4_	self-assembly	CH_4_: 7.24 mmol g^–1^ in 24 d	120 d		^337^
*S. oneidensis*	CdS	Cr^4+^ reduction	in situ assembly	Cr^4+^ reduction: 25 mg/L in 90 min	7.5 h		^339^

#### 6.1.1 H_2_ Production

We begin by discussing the photocatalytic production of H_2_ from water using microbe–semiconductor biohybrid systems.^304^ In these systems, nonphotosynthetic bacteria (e.g., *E. coli*, *R. capsulatus*, and *S. oneidensis*) express hydrogenases ([NiFe]- and [FeFe]-types) that catalyze H_2_ production by accepting photogenerated electrons from a semiconductor. Since hydrogenases are typically located within the cytoplasm or periplasm, an effective interface must facilitate electron transfer across the bacterial membrane. A common strategy employs soluble redox mediators like flavins to shuttle electrons from the semiconductor into the cell.^305–307^ For example, in a TiO_2_/MV/*E. coli* hybrid system, the MV^2+^/MV^+^• redox couple enabled continuous electron transfer, thereby enhancing biocatalytic H_2_ production.^308^ Compared with pure *E. coli*, the TiO_2_/MV^2+^/*E. coli* hybrid system exhibited a 2.8-fold increase in H_2_ production within 15 h. However, such mediators pose challenges related to cost, stability, and biocompatibility, making them less favorable for long-term applications. Wang et al. utilized water-soluble oligofluorene (OF) and polythiophene (PTP) to integrate with *Rhodopseudomonas palustris* (*R. palustris*) for improved solar-powered H_2_ production (Figure 25a).^309^ Under irradiation, the fluorescence resonance energy transfer (FRET) between OF and PTP amplified photoelectron signals and then transported electrons to MV^2+^, which facilitated electron transfer from organic materials to *R. palustris* toward H_2_ evolution.

**Figure 25:**
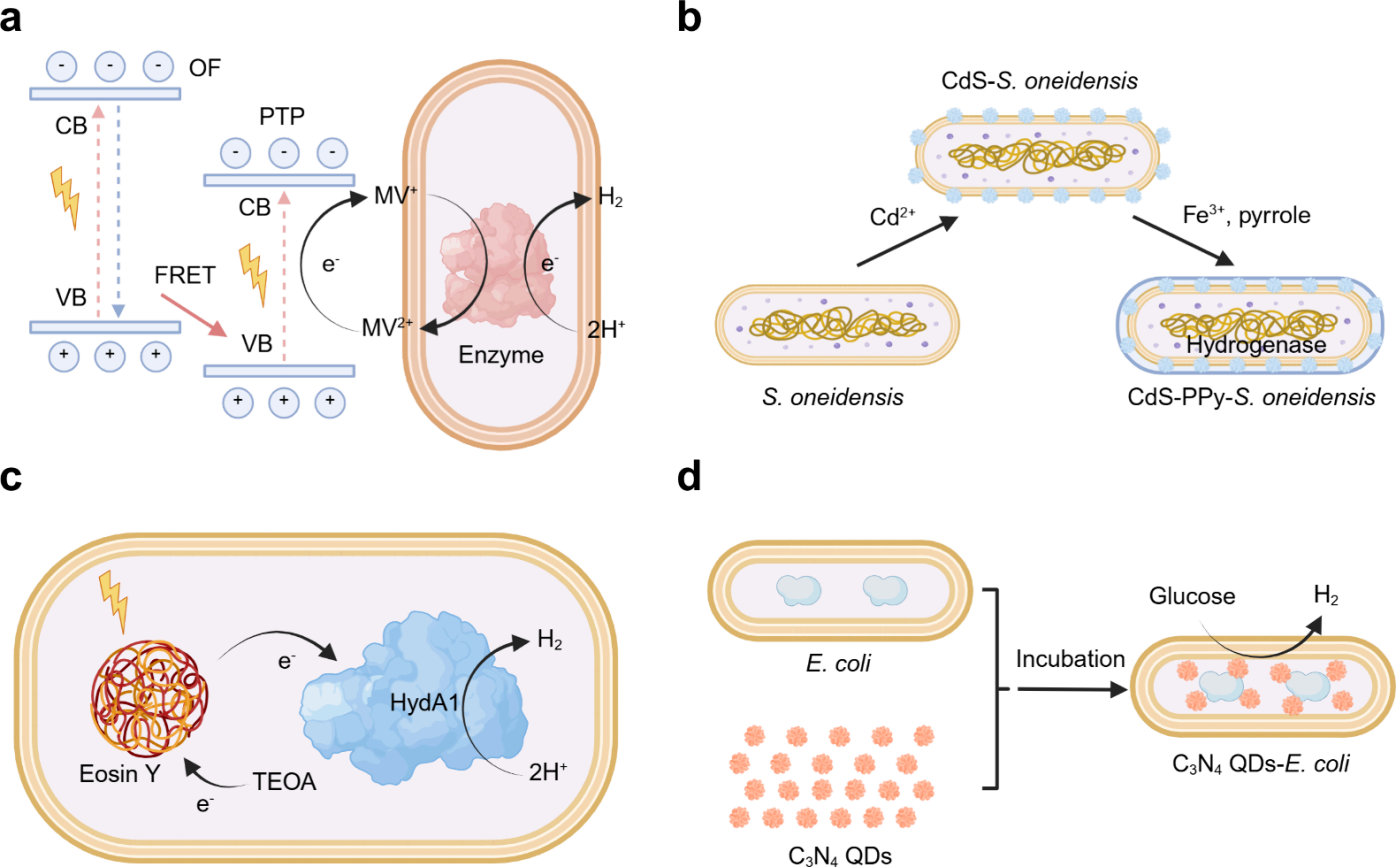
Microbe–semiconductor biohybrids for H_2_ production. (a) Schematic diagram of H_2_ production using the OF/PTP/MV^2+^/TEOA/*R. palustris* complex. (b) Scheme of CdS-PPy–*S. oneidensis* biohybrid system for H_2_ production. (c) Schematic representation of the whole-cell photocatalytic system. Upon photoexcitation, eosin Y facilitates electron transfer between TEOA and HydA1, which ultimately produces H_2_. (d) C_3_N_4_ QDs–*E. coli* biohybrid system for solar-driven H_2_ production. Reproduced with permission from ref 309. Copyright 2021Royal Society of Chemistry. Reproduced with permission from ref 311. Copyright 2024 American Chemical Society. Reproduced with permission from ref 312. Copyright 2022American Chemical Society. Reproduced with permission from ref 241. Copyright 2022 Elsevier.

A more robust approach is to employ electroactive microbes that possess transmembrane redox conduits, allowing for effective electron transfer from semiconductors. In a notable example, CdS nanoparticles were self-precipitated onto the surface of *G. sulfurreducens*, forming a close interface that facilitated electron transfer into the cell upon illumination.^310^ However, despite this improved contact, limitations remain due to slow transmembrane electron transport and limited availability of membrane-bound redox proteins, which reduce the efficiency of electron uptake. To mitigate these issues, composite conductive materials at biohybrid interfaces have been developed. For example, the combination of the conductive polymer PPy as a cell coating with biomineralized CdS nanoparticles formed a cohesive whole-cell electron-collecting layer, minimizing charge leakage and improving overall photoelectron harvesting efficiency (Figure 25b).^311^

Beyond surface-contact strategies, a more advanced approach involves internalizing semiconductor nanomaterials into microbial cells to minimize electron transport distances. For instance, Lorenzi and colleagues utilized Eosin Y as an intracellular photosensitizer to directly transfer photogenerated electrons to heterologous [FeFe] hydrogenase in engineered *E. coli* for H_2_ production (Figure 25c).^312^ Similarly, C_3_N_4_ QDs were successfully delivered into the cytoplasm of *E. coli* (Figure 25d), where hydrogenases are located, resulting in an impressive hydrogen production rate of 7.8 mmol g^–1^ h^–1^, which highlights the advantage of short-range and localized charge transfer in biohybrid systems.^241^

#### 6.1.2 CO_2_ Reduction

One of the key appeals of microbe–semiconductor biohybrids for synthetic chemistry lies in their unique ability to convert CO_2_ into multicarbon products through native carbon assimilation pathways,^115^ such as the Wood–Ljungdahl pathway and the Calvin cycle, which remains challenging for purely synthetic catalysts.^121,313^ Reported systems have successfully produced formic acid, methane, and acetate from CO_2_ under solar illumination.^314–316^

As with H_2_-producing biohybrids, efficient delivery of electrons to intracellular CO_2_-reducing enzymes remains a bottleneck, often necessitating the use of redox mediators. In many examples using acetogenic bacteria (e.g., *S. ovata*, *C. ljungdahlii*, *M. thermoacetica*), photo­(electro)­chemical H_2_ evolution by the semiconductor plays a central role in feeding electrons into the Wood–Ljungdahl pathway.^154^ We demonstrated a Z-scheme photocatalyst sheet (Cr_2_O_3_/Ru–SrTiO_3_: La, Rh|ITO|RuO_2_–BiVO_4_: Mo) integrated with *S. ovata* (Figure 26a), enabling selective acetate production from CO_2_ and H_2_O under sunlight without sacrificial electron donors.^317^ Cr_2_O_3_/Ru and RuO_2_ acted as cocatalysts for H_2_ and O_2_ evolution, respectively. The evolved H_2_ or photogenerated electrons were transported for bacterial metabolism, achieving 90% acetate selectivity and a solar-to-acetate efficiency of 0.70% ± 0.04% (Figure 26b).

**Figure 26:**
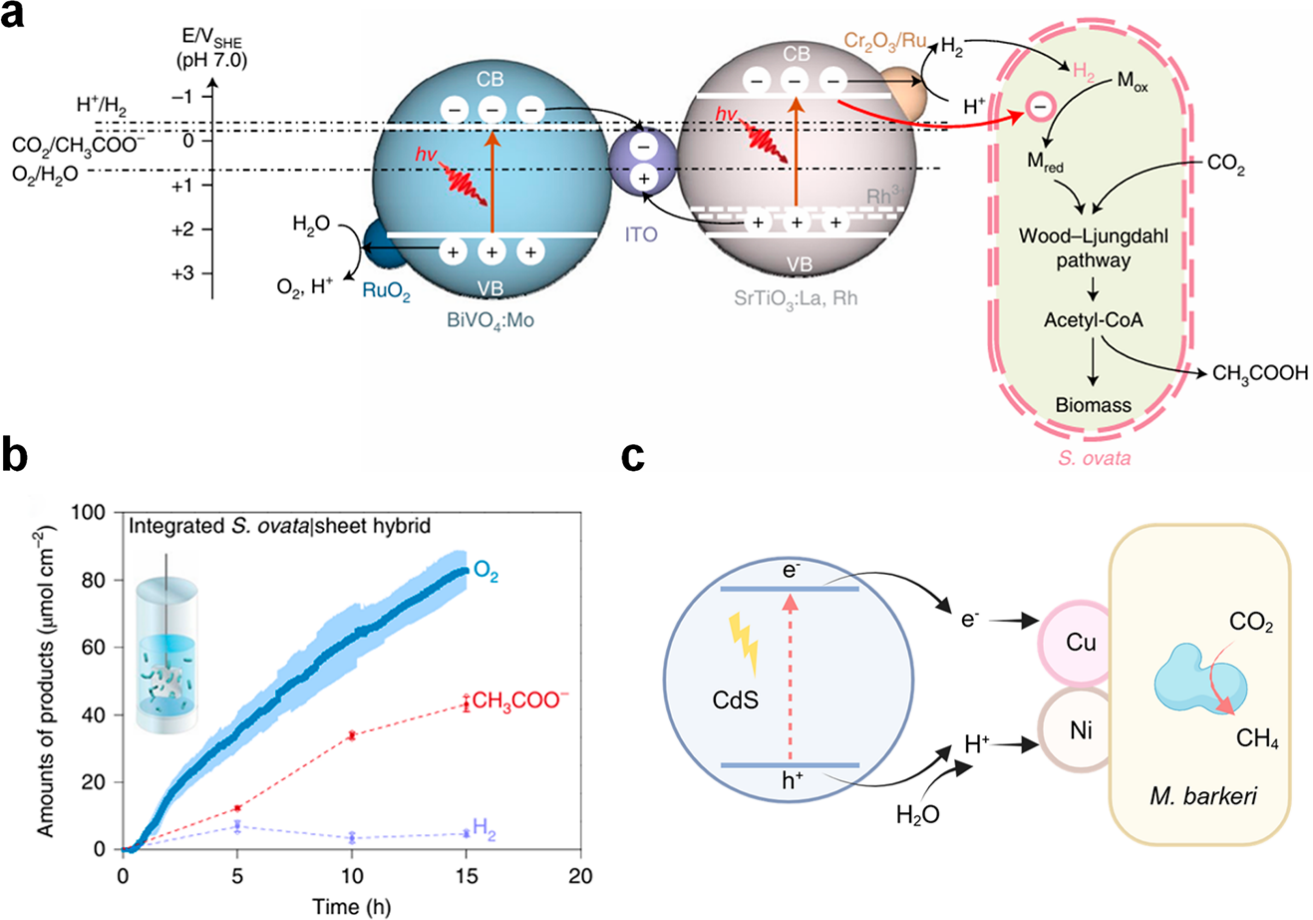
Microbe–semiconductor biohybrids for CO_2_ reduction. (a) Schematic illustration of Cr_2_O_3_/Ru-SrTiO_3_:La,Rh|ITO|RuO_2_–BiVO_4_:Mo–*S. ovata* biohybrid system for CO_2_-to-acetate photoconversion coupled with water oxidation. (b) Photosynthetic CH_3_COO^–^, O_2_, and H_2_ production over Cr_2_O_3_/Ru-SrTiO_3_:La,Rh|ITO|RuO_2_–BiVO_4_:Mo–*S. ovata* hybrids for 15 h. (c) Scheme of CdS/NiCu–*M. barkeri* biohybrids for CO_2_-to-CH_4_ photoconversion. Reproduced with permission from ref 317. Copyright 2022 Springer Nature. Reproduced with permission from ref 220. Copyright 2022 Springer Nature.

In parallel, direct electron transfer has also been explored by precipitating CdS nanoparticles onto acetogens, reportedly enabling direct photogenerated electron uptake for CO_2_-to-acetate conversion.^201^ Modifying the microbial species, such as replacing acetogens with methanogens like *M. barkeri* while maintaining the CdS interface, allows selective methane production, showcasing the modularity and tunability of biohybrid systems.^220^ Specifically, Ni and Cu were selected at the interfaces owing to their suitable work functions and binding strength for hydrogen atoms (Figure 26c). This feature enhanced the biohybrid system by promoting the hydrogen and electron flow from CdS to binary NiCu alloys and finally to *M. barkeri* for CO_2_-to-CH_4_ conversion. Remarkably, the CdS/NiCu–*M. barkeri* hybrids exhibited a high quantum yield of 12.4 ± 0.2% together with a nearly 100% selectivity of CH_4_ production. This work provided deep insight into the design of microbe–semiconductor interfaces to optimize electron transfer and mass transfer for sustainable and selective CO_2_ conversion.

To address limitations in transmembrane electron transfer, intracellular light absorbers offer a more efficient solution. For example, an intracellular CdTe QDs was combined with *E. coli* to build the microbe–semiconductor biohybrids for photocatalytic CO_2_ reduction.^318^ The photogenerated electrons from CdTe QDs could induce the enhanced production of NADH in *E. coli*, further driving CO_2_ reduction pathways for the synthesis of formate and pyruvate.

#### 6.1.3 N_2_ Fixation

Ammonia is a cornerstone of global agriculture and chemical manufacturing, but its conventional production via the Haber–Bosch process requires high temperatures and pressures, leading to significant energy consumption and greenhouse gas emissions.^319^ As a more sustainable alternative, photocatalytic N_2_ reduction has garnered increasing attention. However, current systems suffer from low efficiency and poor selectivity.^320,321^ In contrast, nitrogen-fixing microorganisms efficiently convert atmospheric N_2_ into NH_3_ using nitrogenase enzymes, a key process in the natural nitrogen cycle.^322^ Integrating these biological pathways with light-harvesting materials offers a promising strategy to improve both the efficiency and selectivity of artificial nitrogen fixation under ambient conditions.^323,324^

One representative approach involves directly interfacing semiconducting materials with the surface of nitrogen-fixing bacteria. For example, a solar-driven biohybrid consisting of *A. chroococcum* and a cationic conjugated polymer, PFP, has been shown to significantly enhance nitrogenase activity.^202^ PFP binds electrostatically to the negatively charged bacterial surface, enabling close contact between the synthetic polymer and the cell. Upon illumination, photoinduced electron transfer from PFP to bacterial redox proteins occurs, achieving efficient NH_3_ production. In another configuration, semiconductors have been integrated into bacterial membranes (Figure 27a).^325^ Specifically, a light-harvesting conjugated oligoelectrolyte (COE-IC) was incorporated into the membrane of *A. vinelandii* (Figure 27b). COE-IC, designed with an acceptor–donor–acceptor structure and broad visible-light absorption, facilitated the generation of photoelectrons with suitable potentials for thermodynamically favorable electron transfer to Fd/Fld and ultimately to intracellular nitrogenase upon illumination (Figure 27c).^325^ This membrane-integrated biohybrid showed a 2.4-fold increase in ammonia production along with enhanced biomass and amino acid output, demonstrating the promise of membrane-localized organic photocatalysts.

**Figure 27:**
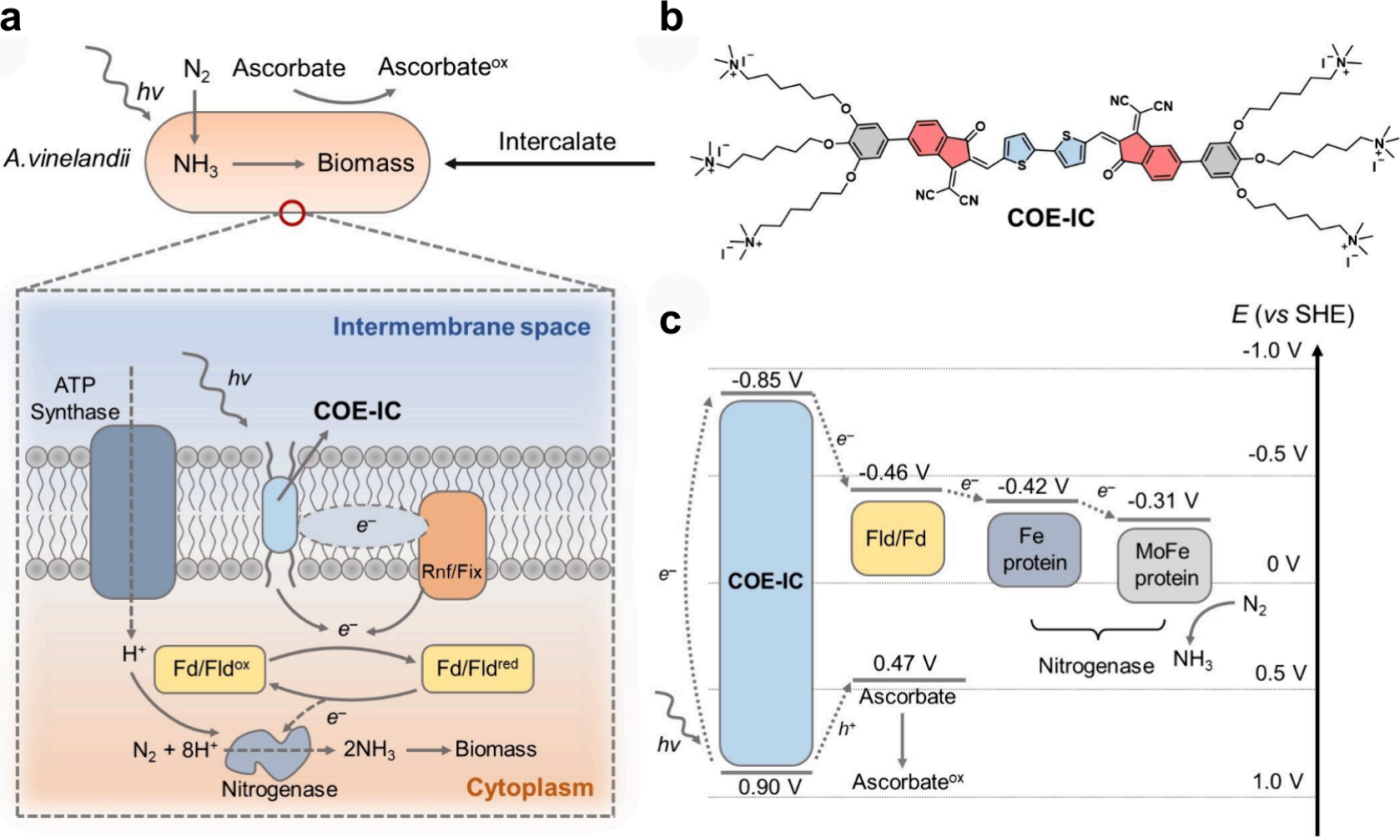
Microbe–semiconductor biohybrids for N_2_ fixation. (a) Schematic diagram and redox potentials of the COE-IC–*A. vinelandii* biohybrid system. (b) Molecular structure of the COE-IC. (c) Scheme of electron transfer in the COE-IC–*A. vinelandii* biohybrid system. Fd/Fld, ferredoxin/flavodoxin; Rnf/Fix, rhodobacter nitrogen fixation enzyme complexes. Reproduced with permission from ref 325. Copyright 2023 Wiley-VCH.

Beyond surface interfacing, another popular strategy is to localize semiconducting nanoparticles intracellularly, bringing them into close proximity with nitrogenases. A notable example involves *A. vinelandii* cultured with InP/ZnSe QDs, which are internalized during the bacterial log phase.^326^ These QDs act as intracellular photocatalysts, transferring photoexcited electrons directly to the MoFe protein of nitrogenase, resulting in a more than 5-fold enhancement in ammonia production under illumination.

An alternative approach employs a coculture strategy inspired by natural microbial consortia. For example, a cascade system for CO_2_ and N_2_ reduction using biohybrids composed of silicon nanowires, *S. ovata*, and *R. palustris*.^327^ In this system, Si nanowires serve as a light-harvesting scaffold. Upon illumination, photogenerated electrons are transferred to *S. ovata* to produce acetate, which is then consumed by *R. palustris* for the NH_3_ biosynthesis from N_2_. Driven by a photovoltaic device, the system achieved solar-to-chemical efficiencies of 1.78% for acetate, 0.51% for nitrogenous biomass, and 0.08% for ammonia production.

In the first demonstration of a PEC microbial biohybrid for N_2_ fixation, *A. vinelandii* was interfaced with an electrode composed of PDA-coated nickel oxide nanosheets (NiO@PDA).^219^ Here, NiO functioned as the light-harvesting component, while the PDA layer provided a biocompatible and conductive interface that promoted robust bacterial adhesion and efficient charge transfer across the abiotic–biotic junction. This rational design enabled a record-high ammonia production rate of 1.85 μmol h^–1^ per 10^8^ cells (4.14 μmol h^–1^ cm^–2^) under illumination. A recent example is to employ Cu_2_O@TiO_2_ NWs to combine with *A. vinelandii* for photoelectrochemical N_2_ fixation.^185^ The core–shell Cu_2_O@TiO_2_ NWs as the photocathode could absorb sunlight and generated photoelectrons to *A. vinelandii*, which served as a biocatalyst for convert N_2_ into NH_3_, and the optimized biohybrids achieved an remarkable NH_3_ production yield of (1.49 ± 0.05) × 10^–9^ mol s^–1^ cm^–2^.

#### 6.1.4 Chemical Synthesis

As we mentioned before, one of the main advantages of biotic–abiotic biohybrid systems is to synthesize complex molecules with high specificity.^173,328^ Herein, we will highlight some typical examples of microbe–semiconductor biohybrids for the synthesis of higher value products, such as PHB and shikimic acid.^329^ Engineering a direct interfacial connection between semiconductors and bacteria is an effective approach to facilitating electron transfer. Wang et al. employed the in situ bioprecipitated method to load CdS nanoparticles on the surface of *R. palustris*.^330^ The photogenerated electrons from CdS nanoparticles provided additional reducing equivalents for *R. palustris* to enable the production of carotenoids and PHB from CO_2_. The carbon nitride nanosheets (CNNS)–*C. necator* biohybrids has also been constructed for the synthesis of PHB (Figure 28a).^8^ The CNNS could form an encapsulation structure to envelope bacterial cells and enhance the contact area at the biohybrid interfaces. Remarkably, this biohybrid system could realize a PHB production rate of 37.25 ± 0.9 mg L^–1^ d^–1^. Mechanistic investigations showed that the CNNS could facilitate the synthesis of H_2_ and other reductive equivalents, ultimately driving the Calvin cycle to convert CO_2_ into PHB.

**Figure 28:**
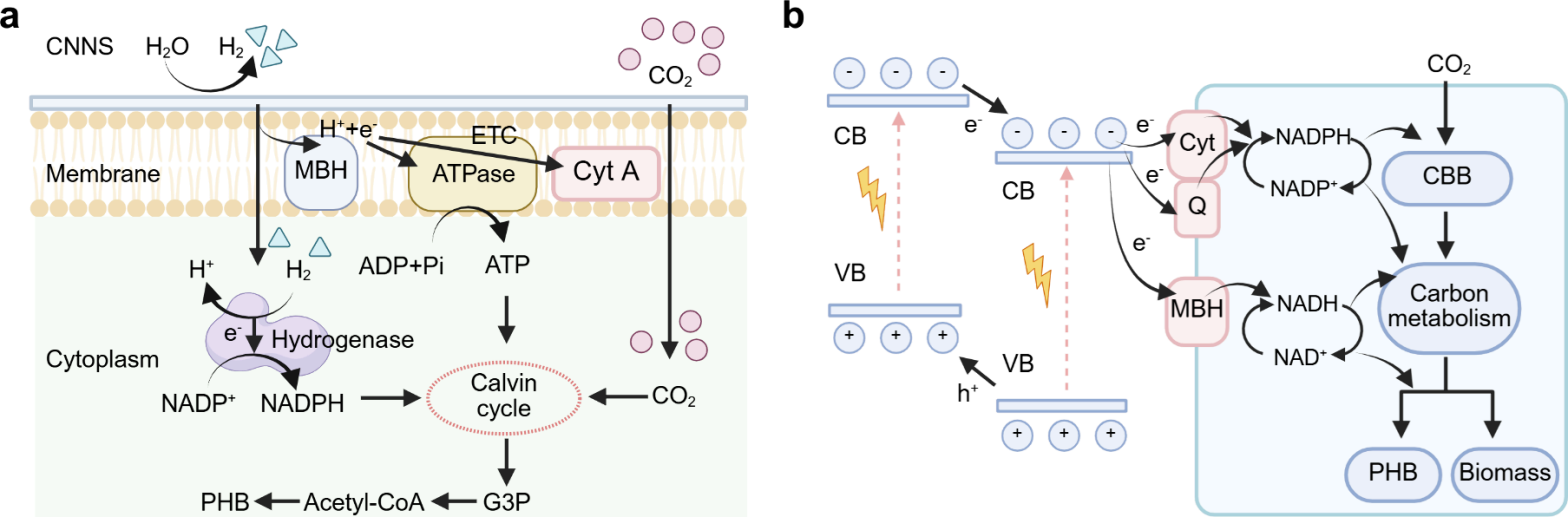
Microbe–semiconductor biohybrids for chemical synthesis. (a) Schematic illustration of CO_2_ conversion and electron transfer in the CNNS–*C. necator* biohybrid system. (b) Scheme of CO_2_ photoreduction to PHB in the organic semiconductor–*C. necator* hybrid system. MBH, membrane-bound hydrogenase; Cyt, cytochrome; Q, ubiquinone, or menaquinone. Reproduced with permission from ref 8. Copyright 2025 American Chemical Society. Reproduced with permission from ref 331. Copyright 2025 American Chemical Society.

A well-defined contact between the semiconductor and the bacterial cell membrane with a strong binding affinity and good biocompatibility is required to facilitate interfacial charge transfer. Zhang et al. utilized different organic semiconductors to combine with *C. necator* to achieve highly efficient photocatalytic CO_2_ reduction to PHB (Figure 28b).^331^ The zwitterionic side chains of the organic semiconductors could generate quadrupoles with phosphatidyl choline on the bacterial cell membrane, facilitating interfacial connection between organic semiconductors and *C. necator*. As a result, the biohybrid system realized a high production yield of 107.3 mg L^–1^ OD_600_^–1^ and a superior quantum efficiency of 1.14% for CO_2_-to-PHB. This work enlightens a meticulous design for constructing microbe–organic semiconductor biohybrids for the synthesis of high-value-added chemicals. Guo et al. engineered yeast *Saccharomyces cerevisiae* (*S. cerevisiae*) to suppress the pathway for biologically regenerating NADH so that the carbon flux could be directed to the synthesis of shikimic acid.^332^ In this microbe–material biohybrid system, InP nanoparticles, as the light absorber to reduce NAD^+^ to regenerate NADH abiotically, were also functionalized with polyphenol moieties to promote interfacial contact with *S. cerevisiae*.

Additionally, microbe–semiconductor biohybrids have also shown great potential for microplastics degradation and water remediation.^333–336^ A notable demonstration is the biohybrid system of carbon dot-functionalized polymeric carbon nitrides (CDPCN)–*M. barkeri* for the conversion of poly­(lactic acid) (PLA).^337^ The CDPCN–*M. barkeri* biohybrid system could successfully achieve the photodegradation of PLA to CH_4_ and the photoreduction of CO_2_ to CH_4_, which sheds light on a unique opportunity for the simultaneous use of microplastics and CO_2_. As for the remediation of wastewater, microbe–semiconductor biohybrids like CdS–based biohybrids have also been commonly used.^338^ Zhang et al. designed CdS–*S. oneidensis* biohybrids to achieve the complete removal of 25 mg/L Cr­(VI) within 90 min under light illumination.^339^ Du and colleagues also constructed CdS–*S. oneidensis* biohybrids to realize efficient decolorization of azo dye wastewater.^340^

Microbe–semiconductor biohybrid systems have demonstrated great potential to synthesize some polymers and chemicals.^341^ However, the natural metabolic activities of these bacteria produce only a very limited number of chemicals and relatively low-value products. In the future, further endowed by genetic engineering and metabolic engineering, the biotic–abiotic biohybrid systems will have greater advantages.^342,343^

### 6.2 Current Limitations of Microbe–Semiconductor Biohybrids

Despite recent advances in integrating microbes with light-harvesting semiconductors for solar-to-chemical conversion, significant limitations still exist that hinder the broader deployment and scalability of these biohybrid systems.

A fundamental limitation is the incomplete understanding of electron transfer mechanisms at the microbe–semiconductor interfaces.^344^ While charge flow is often inferred from overall chemical outputs, direct evidence of how and where electrons are transferred via outer membrane cytochromes, redox shuttles, or direct conduction pathways remains limited. This hampers rational design and optimization, as key molecular and interfacial processes are still largely speculative.^345^

Even when electron delivery to microbial cells is successful, the intrinsic kinetics of biological processes remain sluggish, particularly those involving multielectron transformations.^346–348^ For example, the turnover rates of nitrogenases are very low, creating a rate mismatch between photogenerated charge carriers and biological electron utilization.^347^ While certain hydrogenases can exhibit high intrinsic turnover frequencies, the effective catalytic rates of enzymes involved in semibiological photosynthesis are often limited by nonideal operating environments, including suboptimal pH, temperature, electron and mass transport limitations.^349^ Moreover, excess photogenerated electrons that are not effectively utilized can lead to cytotoxic effects and accelerate semiconductor degradation.^350^

In the current biohybrid systems, another major challenge is the inefficient utilization of the solar spectrum. Many semiconductors employed in biohybrids absorb only in the UV or a narrow range of the visible spectrum, leaving a large portion of incident sunlight unexploited.^247^ Light penetration is also limited in turbid culture media, and the inherently low optical cross sections of biohybrids further restrict photon-to-chemical conversion efficiency. Besides, light intensity must be carefully controlled, as many microbes are sensitive to high illumination levels, particularly in the UV range. This imposes an upper limit on usable light intensity, setting a ceiling on the system’s scalability and real-world applicability.

Additionally, the long-term stability and efficiency of biohybrids also remain challenged by the cytotoxicity of semiconductor components, since inorganic materials sometimes contain heavy metals.^37^ Photoactive nanoparticles such as CdS can generate ROS, disrupt cellular membranes, and impair microbial metabolism due to the leaching of heavy metal ions and the formation of photoinduced oxidative holes. For instance, in an InP/ZnSe QDs–*A. vinelandii* biohybrid system for nitrogen fixation, high external QD concentrations led to ROS-mediated membrane damage and nitrogenase inactivation, resulting in a sharp decline in NH_3_ production.^326^ In contrast, controlled intracellular QD loading enhances cell viability and function, highlighting the critical importance of precise nanoparticle localization and dosage for achieving a stable biohybrid performance. In addition to impairing the catalytic activity of the biological component, semiconductor materials in biohybrids can undergo photodegradation or bleaching over time, leading to diminished light-harvesting efficiency and compromising long-term continuous operation. The reliance on additional redox mediators, such as MV, can further introduce cytotoxic effects and pose additional challenges to system stability. Furthermore, the potential leaching of these materials raises significant concerns regarding environmental safety.

## 7 Opportunities and Outlooks

Alleviating dependence on fossil fuels and achieving efficient utilization of solar energy are essential for the sustainable development of human society.^351^ In the past decade, the field of microbe–semiconductor biohybrid systems for solar-to-chemical conversion has witnessed remarkable progress and notable milestones.^172^ The combination of semiconductors with strong light absorption capability and microorganism with high metabolic specificity holds great promise for highly efficient and selective solar-driven biocatalysis.^352,353^ However, practical applications are still hindered by the relatively low solar energy conversion efficiency and poor stability of current biohybrid systems. Therefore, several promising research directions should be further explored to advance microbe–semiconductor biohybrid systems and accelerate the development of semibiological photosynthesis.

### 7.1 Materials Engineering

The key to high solar energy conversion efficiency in the biotic–abiotic hybrid systems is to promote effective electron transfer at the biohybrid interfaces.^354^ Currently, the reported materials in microbe–semiconductor biohybrid systems are largely based on QDs and organic semiconductor polymers.^37,208^ Undoubtedly, these semiconductors are good light harvesters and can convert absorbed photons into photogenerated electrons for the subsequent utilization of intracellular metabolic activity. Nonetheless, most of these materials lack meticulous designs when combining with microorganisms to build biotic–abiotic biohybrids, resulting in sluggish interfacial electron transfer and energy losses.^33^ These unfavorable interfacial contacts and slow electron uptake at the microbe–semiconductor interfaces eventually lead to low solar-to-chemical conversion efficiency. Some achievements in the field of materials science and chemistry offer great promise for optimizing these semiconductor materials to develop microbe–semiconductor biohybrids.

Over the past several years, single atom catalysis has obtained intensive interest due to their maximized atomic utilization and high metal loading efficiency, enabling remarkable success in the enhancement of catalytic performance.^355,356^ The loading of single atoms on semiconductors can not only regulate the electronic structure and light absorption capability but also optimize surface valence and modulate active sites.^357,358^ It is expected that the single atoms at the material–microorganism interface will also affect interfacial interactions and electron transfer dynamics (Figure 29a).^12,359^ This enhanced electron injection from semiconductors to microbes shows great potential for high solar energy conversion efficiency.

**Figure 29:**
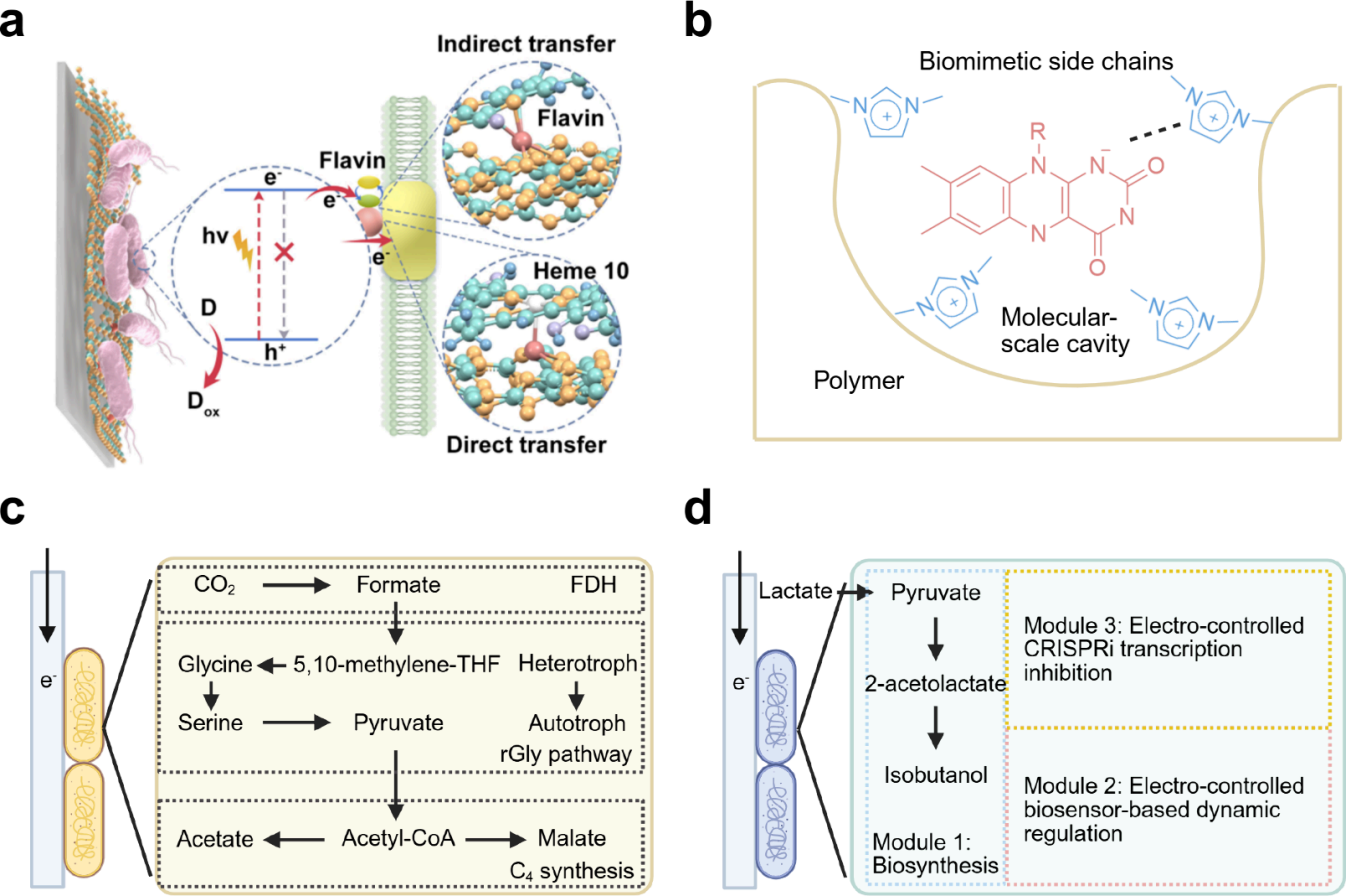
Future opportunities and outlooks for materials engineering and biological engineering. (a) Constructing single-atom at microbe–semiconductor interfaces to enhance charge transfer via cytochrome C-based DET and flavin-mediated MET pathways. (b) Engineering biomimetic side chains at the biohybrid interfaces. (c) Engineered strains with the reductive glycine pathway and the C_2+_ product synthesis module for CO_2_ reduction to malate. (d) Modular design for electro-controlled directional distribution of reducing equivalents to boost isobutanol biosynthesis in microbial electro-fermentation of *S. oneidensis*. Reproduced with permission from ref 12. Copyright 2025 Springer Nature. Reproduced with permission from ref 134. Copyright 2025 American Chemical Society. Reproduced with permission from ref 372. Copyright 2025 American Chemical Society. Reproduced with permission from ref 370. Copyright 2025 Elsevier.

Another promising strategy is constructing a bioinspired interface to realize fast electron transfer at the biohybrid interfaces.^325^ To date, most of the materials employed in biohybrid systems for sustainable energy conversion are not biocompatible, which can be detrimental to cell membrane integrity and intracellular nutrient transport.^360^ Generally, electoactive microbes like *S. oneidensis* behave differently on these nonbiocompatible materials or electrodes in comparison to native substrates.^361^ Although reported semiconductor materials can promote interfacial attachment and enhance total electron transfer, it is still challenging to maximize electron injection on a per cell basis.^362^ Therefore, searching for innovative materials to establish a biocompatible interface for mediating electron transfer is a promising approach.^360,362^ For instance, inspired by the resemblance of a histidine residue present in enzyme active sites, conductive polymers containing imidazolium have been designed to attach at the electrode–*S. oneidensis* interfaces for improved current generation (Figure 29b).^134^ It was observed that the biomimetic conductive polymers mediated two-electron transfer at the polymer–flavin interfaces, mimicking the mechanism of natural flavoenzymes. In the absence of such methylimidazolium functionalized polymers, the flavins could only mediate one-electron transfer. As a result, the biomimetic interface successfully enhanced of per-cell electron uptake by electroactive microbes from the electrode.

### 7.2 Biological Engineering

To promote effective electron transfer and improve the performance of solar-to-chemical conversion, microbial engineering strategies like optimizing EET pathways and modulating intracellular metabolic processes have been widely performed.^363,364^ Looking ahead, some biological engineering approaches, combining synthetic biology, systems biology, and metabolic engineering, provide a great potential to overcome current limitations and establish next-generation microbe–semiconductor biohybrids for highly efficient solar energy conversion.^98,300,365–367^

Recent gene editing tools have focused on either redesigning EET pathways or intracellular metabolic flow,^368^ which still leads to unsatisfactory performance of converting solar energy into chemical energy. Besides, current microorganisms can only utilize injected electrons to produce low-value products, such as H_2_, CH_4_, and acetic acid.^186,232,317,337,369^ These traditional microorganisms can be further engineered to produce high-value target chemicals and fuels with high efficiency.^370^ The combination of engineered electron transfer pathways, intracellular electron transport, and the final metabolic enzymes holds great promise to build an efficient microbial chemical factory.^212^ For example, an integrated bioelectrochemical process to convert CO_2_ to higher alcohols using *C. necator* was developed, in which *R. eutropha* could use the formate from electrocatalytic CO_2_ reduction in fermentation medium to produce isobutanol and 3-methyl-1-butano as the target fuels.^371^ In another example, Li et al. achieved direct bioelectrochemical CO_2_ reduction to C_4_ products of malate in engineered *S. oneidensis* (Figure 29c).^372^ First, the rGly pathway module (plasmid I) was introduced into wild-type *S. oneidensis* MR-1 strains to improve the capability of converting CO_2_ into biomass. Then, the introduction of a malate synthesis module (plasmid II) could enable the successful synthesis of CO_2_ to malate, achieving a high production concentration of 1.18 mmol L^–1^. Yu and co-workers reported a modular design of assembling the electro-controlled distribution system of reducing equivalents and isobutanol biosynthesis pathway in *S. oneidensis* (Figure 29d).^370^ By engineering the isobutanol biosynthesis pathway (module 1), implementing electro-controlled dynamic regulation of NADH/NAD^+^ ratio (module 2), and introducing an electro-controlled CRISPRi transcription inhibition system (module 3), the engineered *S. oneidensis* achieved an isobutanol production of 1320 ± 107 mg/L with a theoretical yield of 94.9%. This represents 10.8- and 10.4-fold enhancement, respectively, compared with the wild-type strain.

Additionally, maintaining microbial viability is equally important for the long-term solar-driven biocatalytic processes in microbe–semiconductor biohybrid systems.^37^ In actual photocatalytic processes, photogenerated charges usually lead to the formation of ROS, which negatively impact microbial survival and activity.^242,373^ To address this challenge, genetic engineering can also be employed to develop microbial strains with enhanced tolerance to oxidative stress, enabling their sustained function under harsh photosynthetic and photoelectrochemical conditions.^37,374^

### 7.3 Advanced Techniques

Even though substantial progress has been made in understanding electron transfer pathways and intracellular metabolic activity, some critical challenges like the underlying mechanisms of dynamic mass transfer and electron transfer should be further addressed.^375^ Both semiconductor materials and microbes evolve with time in biohybrid systems during actual photosynthetic reactions. Such time-resolved behavior would affect the semiconductor distribution, interfacial connections, and microbial viability, resulting in temporary differences of electron transfer and mass transfer as well as the final chemicals metabolism.^290^ Hence, advancing operando and real-time observation to unlock interfacial mass transfer and electron transfer is of great need for the in-depth understanding of biohybrid performance.^37,376^

To be specific, from the perspective of synthetic materials, it remains to be investigated whether semiconductor size-dependent and distribution-dependent electron transfer behaviors occur at microbe–semiconductor interfaces.^236^ In general, different semiconductor sizes have different specific areas and surface energies that can further influence interfacial connection and attachment of bacteria at the interfaces. The locations of semiconductors at the interfaces or within the periplasm possess different electron transfer pathways. Current results confirm that the semiconductors in the periplasm seem more favorable for enhanced metabolic activity.^198^ Considering the ubiquitous heterogeneity of a single bacterium, the total performance of biohybrid photosynthesis lacks enough reliability to support this conclusion. Therefore, more attention should be paid to the behavior of single cells and single organelles.

Besides, advanced techniques developed in bioelectronics and nanobiotechnology can also offer valuable inspiration for semibiological photosynthesis systems.^377,378^ For example, electrical signal transduction involves how external electrical stimuli and interfacial potentials modulate membrane properties, ion transport, and cellular responses.^379,380^ Biochemical signal transduction, such as redox-sensitive regulatory proteins and transcriptional responses, play key roles in how microbe senses and responds to changes in their redox environment.^381^ Although these signals cannot directly help us understand microbe–semiconductor interfacial electron transfer, these processes and techniques will also provide some valuable information about interfacial changes and cellular responses upon light irradiation, which enable more precise control over interfacial changes, membrane decoration, and long-term stability in semibiological photosynthesis system.^382^

Additionally, computational techniques such as density functional theory (DFT) calculations and molecular dynamics (MD) simulations can also provide new insights into interfacial electron transfer processes.^223,383^ Besides, due to the complexity of biohybrid systems, machine learning has the unique advantages of solving multiple-parameter challenges.^384^ Through the input of different parameters such as bacteria numbers, electron transfer impendence, electron injection, and metabolic pathways, we can obtain comprehensive information to optimize interfacial contact, electron transfer, and metabolic processes, guiding the future design of microbe–semiconductor biohybrid systems.

### 7.4 Device Optimization

Device optimization is a critical frontier in advancing the practical application of semiartificial photosynthesis.^16^ While significant progress has been made at the material and biological levels, integrating these components into efficient, scalable, and robust devices remains a key challenge. One promising direction is to design tandem PEC devices or reactions to achieve the synthesis of complex chemicals. Nowadays, the products from both artificial photosynthesis and semiartificial photosynthesis are confined to C_1_ and C_2_ products (e.g., HCOOH, CH_3_COOH), and expanding these simple fuels to more high-value molecules by developing cascade photosynthetic reactions or PEC devices is a promising research direction.^385^

Another major challenge in photocatalytic systems is their reliance on sacrificial electron donors to drive oxidation reactions.^122^ The commonly used sacrificial electron donors in microbe–semiconductor biohybrid systems undermine overall sustainability, incur additional costs, and complicate downstream processing, thereby precluding closed-loop operation.^37^ Besides, water is also used as the electron donor in some reports, but this approach is not fully generalizable since microbes capable of CO_2_ reduction reactions are often strictly anaerobic.^31^ A more practical strategy is therefore to utilize oxidizable waste organic materials, such as pretreated waste plastics and biomass.^386^ Moreover, the oxidation of such organic waste streams or upgrading the biomass-derived chemicals can simultaneously yield value-added products such as formic acid and pentanal.^387,388^

For instance, a recent advancement in CO_2_-fixing biohybrid PEC systems is the development of a bias-free device that utilizes red light (740 nm) to simultaneously drive CO_2_-to-acetate conversion and glycerol oxidation (Figure 30).^389^ This system integrates a p-type silicon nanowire (SiNW) photocathode interfaced with *S. ovata* and a Pt–Au-loaded n-type SiNW photoanode. Under low-intensity illumination (20 mW cm^–2^), the bacteria harness photogenerated reducing equivalents to convert CO_2_ into acetate, while the photoanode oxidizes glycerol, a major byproduct of biodiesel production, into valuable chemicals. The device generates a photovoltage of 0.85 V and achieves a current density of approximately 1.2 mA cm^–2^, maintaining a high Faradaic efficiency of ∼80% for both anodic and cathodic processes. This work highlights a promising strategy for sustainable CO_2_ valorization and waste-to-chemical conversion under mild conditions.

**Figure 30:**
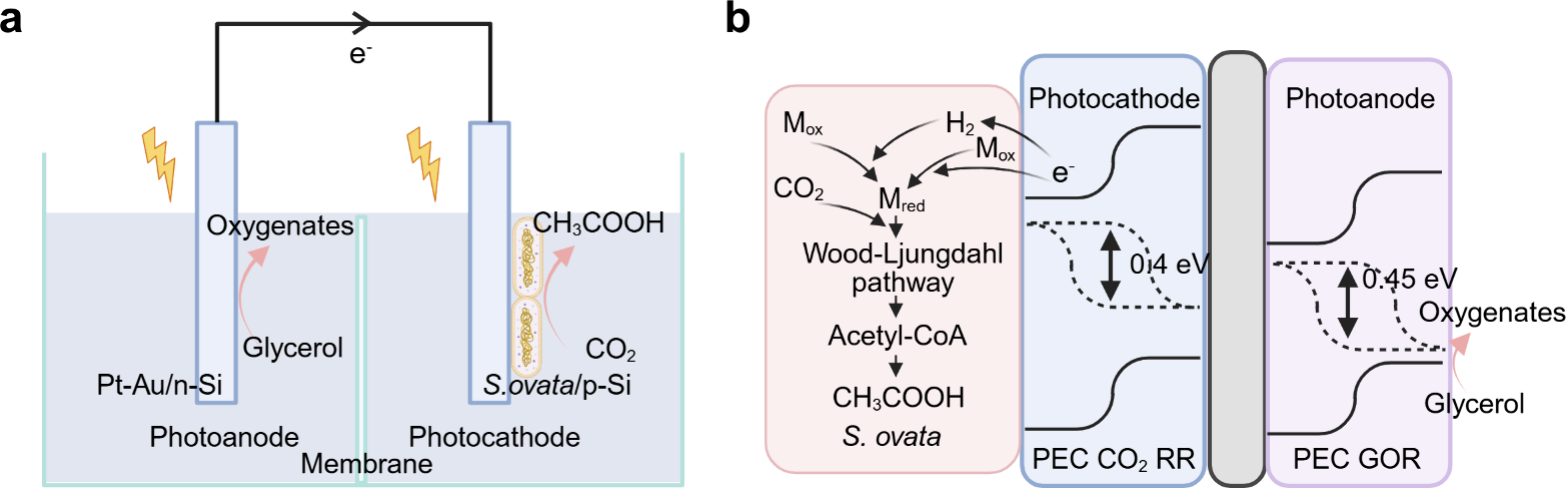
PEC device optimization for semiartificial photosynthesis. (a) Scheme of a red-light-powered silicon nanowire for simultaneous photoelectrochemical CO_2_ reduction and glycerol valorization. (b) Schematic energy diagram of a photochemical diode under red light irradiation. A photovoltage of 0.4 V was harvested at the *S. ovata*/p-Si photocathode and a photovoltage of 0.45 V was harvested at the Pt–Au/n-Si photoanode, simultaneously achieving CO_2_ reduction reaction (CO_2_ RR) and glycerol oxidation reaction (GOR) under red light irradiation without an external bias. Reproduced with permission from ref 389. Copyright 2024 Springer Nature.

Besides, the challenges facing photoelectrochemical semibiological photosynthesis systems include the differences in optimal conditions for the biotic and abiotic components of the device. One of the major differences in performance between the biotic and abiotic components is the conductivity and resistance of the electrolyte. The ionic conductivity of abiotic electrolytes are often >200 mS cm^–1^,^390,391^ with the resistance being <0.1 Ω cm^2^.^392^ Conversely, the ionic conductivity of biotic electrolytes is often around 10 mS cm^–1^,^390,391^ with the resistance being about 100 Ω cm^2^.^393,394^ This can account for the differences in current densities observed for the biotic and abiotic electrochemical systems. The abiotic CO_2_ electrolysis to syngas can have current densities of hundreds of mA cm^–2^,^395^ while the current density of cathodic microbial attachment only ranges from 1 to 10 mA cm^–2^.^396^ Thus, increasing the ionic conductivity of biotic electrolytes can help improve the performance of these devices.^397^ One way is to increase the salt concentration of the electrolyte. For example, Zhang et al. have shown how an electrolyte with seawater salinity can be used with microbes in an electrochemical device to drive the carbon dioxide reduction reaction.^398^ Moreover, increasing the salt concentration of the electrolyte could induce the microbes to form a biofilm,^399^ further enhancing the contact between the cathode and microbe. Once these challenges have been addressed, the field of semiartificial photosynthesis is expected to accomplish greater achievements.^400–402^

### 7.5 Future Outlooks

Microbe–semiconductor biohybrids represent a promising platform for sustainable solar-to-chemical conversion. Although significant progress has been made in recent years, realizing high-efficiency and scalable systems suitable for practical applications will require coordinated advances in materials design, biological engineering, interface characterization, and device integration.^33^

Integration of insights across scales is essential. While the mechanisms of interfacial electron transfer are increasingly well understood at the molecular level, translating this knowledge into predictive design strategies requires close coordination with mesoscale interface engineering. For example, although CdS nanoparticles may exhibit suitable conduction band alignment with microbial redox proteins such as MtrA, poor surface contact or nonuniform nanoparticle distribution can severely limit actual electron transfer. Likewise, intracellular delivery of QDs (e.g., g-C_3_N_4_ or InP/ZnSe into *E. coli* or *A. vinelandii*) can reduce transport distances and improve charge injection efficiency, but optimizing these systems depends on a detailed understanding of their intracellular localization, interaction with redox proteins, and potential cytotoxic effects. Bridging these molecular and structural considerations will be key to advancing rational, high-performance biohybrid designs.

Holistic materials–biology codesign will be central to the next phase of biohybrid development. The next generation of biohybrids must address current limitations in spectral utilization, biocompatibility, turnover rates, and long-term stability. Advances in material science should focus on developing nontoxic, broadband-absorbing semiconductors with extended lifetimes and high charge separation efficiency, moving the field beyond reliance on traditional CdS- or InP-based systems. In parallel, microbial engineering must move beyond native strains toward synthetic hosts optimized for electron uptake and product specificity. For instance, *E. coli*, a versatile model in synthetic biology, can be genetically engineered to express outer membrane electron conduits (e.g., the *S. oneidensis*-derived Mtr pathway) to enable direct electron uptake from semiconductors. Additional metabolic modules can be introduced to enhance catalytic efficiency, such as carbonic anhydrases for CO_2_ hydration or synthetic hydrogenases for H_2_ evolution. To broaden the product scope beyond typical solar fuels, more complex metabolic pathways should be incorporated, enabling the biosynthesis of high-value chemicals and materials. Crucially, host strains must also be engineered for improved tolerance to photoinduced oxidative stress and harsh electrochemical environments. Enhancing resistance to ROS and increasing tolerance to elevated light intensities will be essential for long-term operation in PEC devices. These adaptations will allow biohybrids to operate under more demanding and industrially relevant conditions, bringing the technology closer to practical implementation.

Theory-guided discovery will be increasingly significant in advancing the design of microbe–semiconductor biohybrid system. Machine learning models, trained using experimental data and descriptors from DFT, can help predict suitable combinations of semiconductors, microbial strains, and interface configurations. These tools can guide selection based on parameters such as band edge alignment, charge transport properties, interfacial compatibility, and microbial redox characteristics. When combined with high-throughput experimental screening and in situ characterization techniques, these predictive approaches can significantly accelerate the design cycle. For instance, computationally suggested microbe–material pairings can be tested for electron transfer efficiency and catalytic performance under relevant conditions. Techniques such as operando imaging techniques can then provide mechanistic feedback to improve model accuracy. By linking computational predictions with experimental validation, this integrated approach offers a more efficient route to understanding and optimizing complex biohybrid systems and may reveal design principles that are difficult to access through empirical methods alone.

Device-level innovation represents a critical frontier in advancing microbe–semiconductor biohybrids toward practical applications. Future systems must move beyond batch-mode and proof-of-concept experiments. Emerging directions include PEC cells that couple solar-driven oxidation and reduction reactions, solar-powered cascade systems, and modular photobioreactors designed to process industrially relevant inputs, such as flue gas or wastewater. Realizing these systems will require careful reactor engineering to optimize the photon flux, gas–liquid exchange, and spatial organization of microbial populations, all of which can significantly influence efficiency and stability. Although significant progress has been achieved at the laboratory scale, translating current semibiological photosynthesis systems to industrial applications remains challenging. These barriers include the limited scalability of finely engineered nanostructures and devices, insufficient long-term stability under illumination, and low solar-to-chemical conversion efficiency.

These challenges can be addressed with some useful strategies. From a materials perspective, there is an opportunity to develop more efficient and robust semiconductors that can operate under biologically compatible conditions and maintain long-term stability under continuous illumination. From a biological standpoint, key opportunities include developing microorganisms with improved compatibility with semiconductor interfaces as well as enhanced tolerance to light-induced stress and redox imbalance. Genetic and metabolic engineering can further optimize electron transfer pathways and metabolic networks to maximize electron utilization and improve solar-to-chemical conversion efficiency, while preserving cellular viability. At the device level, designing continuous-flow, bias-free, and fully integrated photobioreactors, as well as scalable modular devices, can offer promising directions. Ultimately, co-optimization and seamless integration of materials and biological components at the device level will be essential for translating solar-to-chemical conversion technologies toward real-world deployment.

Overall, we hope that the multidisciplinary and comprehensive understanding of microbe–semiconductor biohybrid systems and interfacial electron transfer presented here will provide new insights for the future development of semiartificial photosynthesis.

## Abbreviations

PEC: photoelectrochemical
*Cupriavidus necator*: *C. necator*
EET: extracellular electron transfer
PSI: photosystem I
PSII: photosystem II
NADP^+^: nicotinamide adenine dinucleotide phosphate
NADPH: reduced nicotinamide adenine dinucleotide phosphate
ATP: adenosine triphosphate
ADP: adenosine diphosphate
Fd: ferredoxin
PQ: plastoquinone
PC: plastocyanin
PS: photosystem
Pi: inorganic phosphate
RuBisco: ribulose-1,5-bisphosphate carboxylase/oxygenase
PGA: 3-phosphoglycerate
G3P: glyceraldehyde 3-phosphate
RuBP: ribulose 1,5-bisphosphate
UV: ultraviolet
IR: infrared
VB: valence band
CB: conduction band
STC: solar-to-chemical
CN_*x*_/Ni_2_P: carbon nitride/nickel phosphide
MES: microbial electrosynthesis
*S. ovata*: *Sporomusa ovata*
*M. thermoacetica*: *Moorella thermoacetica*
*G. sulfurreducens*: *Geobacter sulfurreducens*
EC: electrochemical
DET: direct electron transfer
MET: mediated electron transfer
*M. barkeri*: *Methanosarcina barkeri*
*R. eutropha*: *Ralstonia eutropha*
PV: photovoltaic
*C. ljungdahlii*: *Clostridium ljungdahlii*
EAMs: electroactive microorganisms
NR: neutral red
*S. oneidensis*: *Shewanella oneidensis*
Mtr: metal-reducing
c-Cyts: c-type cytochromes
CymA: c-type cytochrome A
FccA: fumarate reductase
OmcA: outer membrane cytochrome A
RF: riboflavin
FMN: flavin mononucleotide
*S. baltica*: *Shewanella baltica*
IM: inner membrane
Cbc: cytochrome bc
ImcH: inner membrane cytochrome H
MacA: membrane-associated cytochrome A
PpcA: periplasmic c-type cytochrome A
LET: long-range electron transfer
Hyd: [FeFe]-hydrogenase-based electron-bifurcating complexes
Stn: *Sporomusa* type ferredo-dependent transhydrogenase
Nfn: NADH-dependent ferredoxin:NADP^+^ oxidoreductase.
Ech: energy-converting hydrogenase
Rnf: Rhodobacter nitrogen fixation complex
MFCs: microbial fuel cells
rTCA: reductive tricarboxylic acid
rGly: reductive glycine
^•^OH: hydroxyl radicals
H_2_O_2_: hydrogen peroxide
^•^O_2_^–^: superoxide
QDs: quantum dots
PDI: perylene diimide derivative
PFP: poly­(fluorene-*co*-phenylene)
CDs: carbon dots
g-N-CDs: graphitic nitrogen doped carbon dots
MV: methyl viologen
AQDS: anthraquinone-2, 6-disulfonate
Pdots: polymer dots
RH16: *R. eutropha* H16
PHB: poly-3-hydroxybutyrate
*D. desulfuricans*: *Desulfovibrio desulfuricans*
2D: two-dimensional
RGO: reduced graphene oxide
PPy: polypyrrole
PDA: polydopamine
*R. capsulatus*: *Rhodobacter capsulatus*
*S. xiamenensis*: *Shewanella xiamenensis*
THPC: tetrakis­(hydroxymethyl)­phosphonium chloride
*A. vinelandii*: *Azotobacter vinelandii*
*B. subtilis*: *Bacillus subtilis*
*P. aeruginosa*: *Pseudomonas aeruginosa*
*P. fluorescens*: *Pseudomonas fluorescens*
PHAs: polyhydroxyalkanoates
EPS: extracellular polymer substrates
TRPL: time-resolved photoluminescence
TAS: transient absorption spectroscopy
EIS: electrochemical impedance spectrum
*X. autotrophicus*: *Xanthobacter autotrophicus*
*S. elongatus*: *Synechococcus elongatus*
AFM: atomic force microscopy
PCA: principal component analysis
CBB: Calvin–Benson–Bassham
OF: oligofluorene
*R. palustris*: *Rhodopseudomonas palustris*
PTP: polythiophene
COE: conjugated oligoelectrolyte
FRET: fluorescence resonance energy transfer
CNNS: carbon nitride nanosheets
*S. cerevisiae*: *Saccharomyces cerevisiae*
CDPCN: polymeric carbon nitrides
PLA: poly­(lactic acid)
DFT: density functional theory
MD: molecular dynamics
SiNW: silicon nanowire
